# Adverse events associated with peanut oral immunotherapy in children – a systematic review and meta-analysis

**DOI:** 10.1038/s41598-019-56961-3

**Published:** 2020-01-20

**Authors:** Luke E. Grzeskowiak, Billy Tao, Emma Knight, Sarah Cohen-Woods, Timothy Chataway

**Affiliations:** 1 Robinson Research Institute, Adelaide Medical School, University of Adelaide, Adelaide, SA Australia; 2 SA Pharmacy, SA Health, Adelaide, SA Australia; 3 Department of Paediatrics and Child Health, College of Medicine and Public Health, Flinders University of South Australia, Bedford Park, SA Australia; 4 School of Public Health, University of Adelaide, Adelaide, SA Australia; 5 College of Education, Psychology and Social Work, Flinders University of South Australia, Bedford Park, SA Australia; 6 Flinders Centre for Innovation in Cancer, Flinders University of South Australia, Bedford Park, SA Australia; 7 Proteomics Facility, College of Medicine and Public Health, Flinders University of South Australia, Bedford Park, SA Australia

**Keywords:** Paediatric research, Epidemiology

## Abstract

While peanut oral immunotherapy (POIT) represents a promising treatment for peanut allergies in children, safety concerns remain a common barrier to widespread adoption. We aimed to systematically assess available evidence to determine the risk and frequency of adverse events occurring during POIT, and examine study-level characteristics associated with their occurrence and severity. A systematic search of MEDLINE, EMBASE, and Web of Science was conducted through April 2019. Controlled and non-controlled studies evaluating POIT were eligible. Twenty-seven studies, involving 1488 subjects, were included. Adverse events to POIT were common and led to treatment discontinuation in 6.6% of children (95% CI 4.4–9.0; 27 studies, I^2^ = 48.7%). Adverse events requiring treatment with epinephrine occurred among 7.6% (4.5–11.4; 26 studies, I^2^ = 75.5%) of participants, at a rate of 2.0 per 10,000 doses (0.8–3.7; 15 studies, I^2^ = 64.4). Use of a rush treatment phase and targeting a higher maintenance dose were associated with a higher risk and frequency of epinephrine use, while using co-treatments in addition to POIT was associated with a lower risk of treatment discontinuation due to adverse events. While adverse events to POIT are common, this study provides promising explorative evidence that certain modifications to existing treatment protocols could significantly improve treatment outcomes.

## Introduction

Peanut allergy is the leading cause of food-related allergic reactions for children in Western countries, affecting 1–3% of children^1–5^. While a small percentage of children grow out of their peanut allergy, the only currently recognised and supported treatment approach is allergen-avoidance and use of rescue medications for managing allergic reactions^6^. Avoidance, however, can be difficult because peanuts are widely present in many foods, and there is also the risk of contamination during manufacturing processes. In addition, labelling can be inadequate or misinterpreted by families and caregivers^7^. As such, accidental ingestion leading to reactions are common even under supposedly strict avoidance^8–10^, representing a significant burden on children and their families^11^.

As such, there has been significant interest in developing approaches towards the prevention and treatment of peanut allergies. Oral immunotherapy (OIT) has recently emerged as an effective treatment in desensitising children with a variety of food allergies. While recent systematic reviews have demonstrated the effectiveness of POIT in achieving the immunological goal of desensitisation to peanut allergy, they have raised significant concerns regarding potential risks associated with treatment^12,13^. This has led to caution regarding the adoption of POIT in clinical practice outside of the trial setting. These previous reviews, however, were restricted to randomised controlled trials, omitting a large body of evidence from non-controlled studies that can provide greater evidence surrounding likely real-world outcomes associated with POIT in children. In light of this, we sought to quantify the risk of adverse events occurring during POIT for peanut allergy in allergic children, and examine study and patient level characteristics associated with their occurrence.

## Methods

### Search methods and study selection

This systematic review and meta-analysis was performed in accordance with the preferred reporting items for systematic reviews and meta-analyses (PRISMA) guidelines (S1 Table)^14^. We searched three electronic databases from inception to 9^th^ April 2019: Ovid MEDLINE, EMBASE, and Web of Science. Medical subject headings (e.g. MeSH headings) and free word combinations using Boolean logic of the following search items were used: ‘peanut’ AND ‘immunotherapy’ (S1 Appendix). Previous reviews, bibliographies of published trials, and cross references were also searched. No language restrictions were applied.

### Study selection

Eligible studies included prospective controlled or non-controlled studies involving the administration of POIT in children less than 18 years of age that reported on safety and/or efficacy outcomes. Studies were not eligible for inclusion if they were only published in abstract form. Two independent reviewers (LG and BT) screened the titles and abstracts of identified studies. Disagreements were resolved through consensus or consultation with a third independent reviewer (TC).

### Data extraction and outcomes of interest

Two reviewers (LG and BT) utilized a standardized data extraction sheet to independently extract the following data: study characteristics, patient characteristics, and treatment outcome measures. Disagreements were resolved through consensus or consultation with a third independent reviewer (TC). We did not contact the authors of eligible studies for additional data.

The primary outcome was adverse events resulting in treatment discontinuation. Secondary outcomes included: adverse events requiring any treatment, adverse events requiring treatment with epinephrine, ability to reach target maintenance dose, and passing supervised oral food challenge following OIT. For the outcome of passing supervised oral food challenge, this had to occur immediately post-intervention. Studies where the supervised oral food challenge only occurred following a period of treatment discontinuation (i.e. to evaluate sustained unresponsiveness) did not contribute data to this outcome analysis. Where available, we extracted outcomes on adverse events occurring during each treatment phase including: rush, build-up, or maintenance. Study quality was not formally evaluated due to an absence of validated tools for evaluating risk of bias in the context of this type of systematic review. However, as a key marker of study quality and identified source of potential reporting bias, for all outcomes we stratified studies according to whether subjects were blinded to POIT or not.

### Data analysis

Each outcome was analysed according to intention-to-treat (ITT). For dichotomous outcomes (e.g. treatment discontinuation) we utilised the total number of subjects who initially received the intervention as the denominator. For outcomes that could occur more than once in the same patient (e.g. number of adverse events) we utilised the total number of doses received as the denominator. Binary outcome data were first transformed via the Freeman-Tukey double arcsine transformation (and then back transformed where possible to show estimates and confidence intervals as percentages), ensuring studies reporting percentages of 0 or 100% were included in the meta-analysis^15^. Transformations also ensured that reported pooled proportions did not fall outside of the valid range (0 to 100%). We pooled summary measures using the *‘metaprop_one’* package in STATA 14 (StataCorp LP, College Station, TX) with 95% confidence intervals calculated using the Wilson method^16^. Univariate meta-regressions of the effects of continuous study-level factors at baseline were performed using the *‘metafor’* and *‘metan’* packages in R (version 3.2.1, The R Foundation, Vienna, Austria)^17^. For all summary estimates we specified a random-effects model using the method of DerSimonian and Laird, with the estimate of heterogeneity being taken from the inverse-variance fixed-effect model. Between-study heterogeneity was quantified by the I^2^ statistic, with sources of heterogeneity explored through subgroup comparisons or meta-regression. Bias relating to study effect was assessed with funnel plots and Egger’s asymmetry test^18^.

### Subgroup comparisons

For each of the outcomes, a range of subgroup comparisons were undertaken. Pre-specified subgroup comparisons included treatment related factors including use of a rush phase, co-treatment with additional study medication (e.g. omalizumab), and blinding to treatment allocation. Following the publication of a recent meta-analysis on POIT^12^ we added additional subgroup comparisons including the target maintenance dose, and use of entry oral food challenge (OFC) to determine eligibility. Further, patient related factors included child age, baseline peanut specific IgE (psIgE) and Skin Prick Test (SPT) values, and proportion of children with co-morbidities such as asthma, allergic rhinitis, eczema, or other food allergies.

## Results

### Study search

A total of 2694 titles were identified, which led to 33 articles being analysed for full-text review (S1 Figure). After full-text review, 27 published studies involving 1,488 children receiving POIT met the inclusion criteria^19–45^.

### Included studies

Table 1 details the characteristics of all the studies included in this review. Among the 27 included studies, 12 (44%) were randomised controlled trials. Of the 12 RCTs, comparator arms included randomisation to placebo (n = 6), peanut avoidance (n = 3), sublingual OIT (n = 1), omalizumab (n = 1), or different POIT maintenance doses (n = 1). A co-treatment used in conjunction with POIT was employed by 8 (30%) studies (S2 Table). The majority of included studies (n = 20; 74%) incorporated an OFC as part of the eligibility criteria. Seventeen (63%) studies incorporated a rush phase as part of their treatment protocol, typically involving the rapid escalation of doses over 1-day. The target maintenance dose ranged from 125 mg to 5000 mg peanut protein daily. No significant differences in POIT protocols were evident between controlled and non-controlled studies (S3 Table).

**Table 1:** Characteristics of included peanut oral immunotherapy studies.

Study/Year [Country]	RCT	Blinded OIT	Comparator	Co-Treatment	Entry OFC Type	Rush Phase	Peanut OIT Dose (mg peanut protein)		Exit OFC	Subjects Receiving OIT
							Starting	Target Maintenance		
Anagnostou 2011 [UK]	No	No	None	None	DBPCFC	No	0.5	800	Open	22
Anagnostou 2014 [UK]	Yes	No	Peanut Avoidance	None	DBPCFC	No	2	800	DBPCFC	94
Bird 2015 [USA]	No	No	None	None	DBPCFC	No	Variable	2000	DBPCFC	11
Bird 2018 [USA]	Yes	Yes	Placebo	None	DBPCFC	Yes	0.5 to 6	300	DBPCFC	29
Blumchen 2010 [Germany]	No	No	None	None	DBPCFC	Yes	Variable	125	DBPCFC*	23
Blumchen 2019 [Germany]	Yes	Yes	Placebo	None	Open	No	Variable	125 or 250	Open	31
Fauquert 2018 [France]	Yes	Yes	Placebo	None	DBPCFC	No	2	400	DBPCFC	21
Hofmann 2009 [USA]	No	No	None	None	None	Yes	0.1 to 50	300	None	28
Howe 2019 [USA]	Yes	No	Symptoms as side effects (n = 24) or symptoms as positive signals (n = 26)	Antihistamine	None	No	1.3	240	None	50
Jones 2009 [USA]	No	No	None	None	None	Yes	0.1 to 50	300	Open	39
Kukkonen 2017 [Finland]	No	No	Peanut Avoidance	Antihistamine	DBPCFC	No	0.1	800	DBPCFC	39
MacGinnitie 2017 [USA]	Yes	No	Omalizumab + Oral OIT [n = 27] Vs. Oral OIT alone [n = 8]	Omalizumab	DBPCFC	Yes	0.5 to 250	2000	Open	35
Nachshon 2018 [Israel]	No	No	None	None	Open	Yes	Variable	1200 or 3000	Open	145
Nagakura 2018a [Japan]	No	No	None	Antihistamine & Montelukast	DBPCFC	Yes	Variable	795	OFC*	22
Nagakura 2018b [Japan]	No	No	None	Antihistamine	Open	Yes	Variable	133	OFC*	24
Narisety 2015 [USA]	Yes	No	Oral Vs. Sublingual OIT	None	Open	Yes	0.1 to 6	2000	Open	11
Nozawa 2014 [Japan]	No	No	None	None	DBPCFC	Yes	Variable	1750 or 3500	None	18
PALISADE 2018 [North America & Europe]	Yes	Yes	Placebo	None	DBPCFC	Yes	0.5 to 6	300	DBPCFC	372
Reier-Nilsen 2019 [Norway]	Yes	No	Peanut Avoidance	None	DBPCFC	No	5 or 1	5000	None	57
Schneider 2013 [USA]	No	No	None	Omalizumab	DBPCFC	Yes	0.05 to 250	4000	DBPCFC	13
Tang 2015 [Australia]	Yes	Yes	Placebo	Probiotic^†^	None	Yes	0.1 to 12	2000	DBPCFC	31
Tao 2017 [Australia]	No	No	None	None	Open	No	Boiled	2500	Open	14
Varshney 2011 [USA]	Yes	Yes	Placebo	None	None	Yes	0.1 to 6	4000	DBPCFC	19
Vickery 2017 [USA]	Yes	No	300 mg [n = 20] Vs. 3000 mg [17] maintenance dose	None	Open	Yes	0.05 to 3	300 vs. 3000	DBPCFC	37
Wasserman 2019 [USA]	No	No	None	None	None	Yes	0.001 to 10 mg/0.002 to 2.05 mg	3000	None	270
Yu 2012 [USA]	No	No	None	None	None	Yes	Variable	4000	None	24
Zhong 2019 [Singapore]	No	No	None	Probiotic^†^	Open	No	0.5	3000	Open	9

Abbreviations: RCT, randomised controlled trial; OIT, oral immunotherapy; OFC, oral food challenge; DBPCFC, double blind placebo controlled food challenge.

*OFC completed after 2 weeks of peanut avoidance.

^†^Lactobacillus rhamnosus.

Substantial heterogeneity was evident with respect to study population (S4 Table). Median age was 8.4 years and ranged from 4.8 to 14.5 years. Median psIgE ranged from 9.6 to 229 (median 67.5), while SPT ranged from 7 to 17.6 (median 11).

### Risk of adverse events resulting in treatment discontinuation

**Figure 1:**
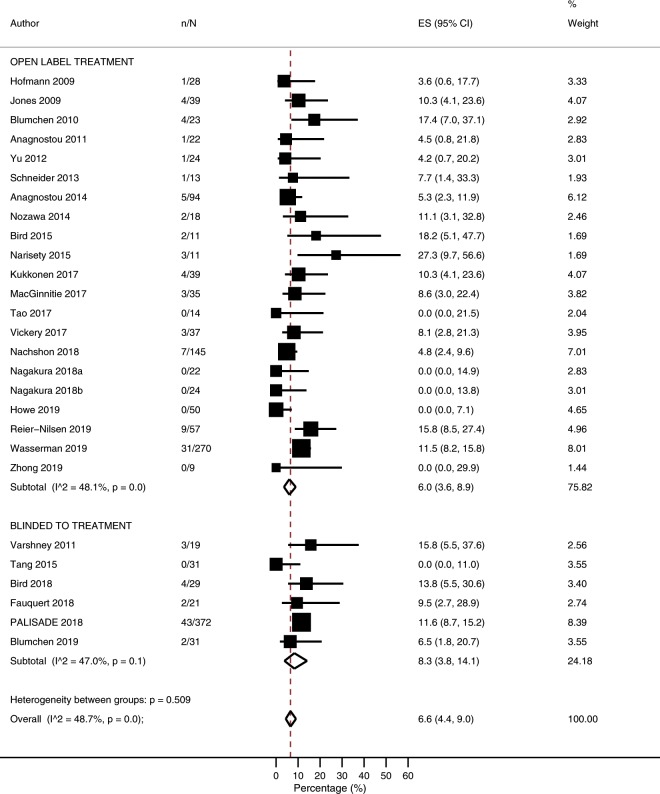
Proportion of participants experiencing adverse events leading to treatment discontinuation.

The overall risk of treatment discontinuation due to adverse events was 6.6% (95% CI 4.4–9.0%; I^2^ = 48.7%; N = 27 studies, Fig. 1), with no evidence of publication bias relating to small study effects (p = 0.352, S2 Figure). Risk of treatment discontinuation due to adverse events was lower among studies which included co-treatment alongside POIT (1.4%; 0–5.2%) compared with studies which used POIT alone (8.5%; 6.5–10.8%; p = 0.003) (Table 2*,* S3 Figure). When stratified according to co-treatment type, a lower risk of treatment discontinuation due to adverse events was noted among studies that administered probiotics or antihistamines, but not omalizumab (Table 2*,* S3 Figure). Following univariate meta-regression, a higher risk of treatment discontinuation due to adverse events was associated with higher baseline peanut specific psIgE (p = 0.0059), and a higher percentage of children with asthma (p = 0.0402) (S5 Table*,* S4 Figure).

**Table 2:** Proportion of participants experiencing adverse events leading to treatment discontinuation according to study level characteristics.

Comparison	Studies	% (95% CI)	I^2^	p value
Overall	27	6.6 (4.4–9.0)	48.7	NA
**Rush Phase**				
Yes	17	7.3 (4.7–10.3)	47.2	0.532
No	10	5.3 (1.8–10.1)	50.1	
**Co-Treatment**				
No	20	8.5 (6.5–10.8)	17.7	0.003
Yes	8	1.4 (0–5.2)	39.5	
*Antihistamine Alone*	*3*	*10.3 (4.1–23.6)*	NA	
*Antihistamine* + *Montelukast*	*1*	*0.0 (0.0–14.9)*	NA	
*Omalizumab*	*2*	*7.2 (0.5–14.9)*	NA	
*Probiotic*	*3*	*0.0 (0.0–3.9)*	NA	
**Maintenance Dose** (**mg/day**)				
<1000	14	5.6 (2.8–9.1)	53.8	0.328
≥1000	12	7.7 (4.1–12.1)	50.4	
**Entry OFC**				
DBPCFC	13	9.2 (6.8–12.0)	7.8	0.086
Open	7	3.8 (0.7–8.4)	31.9	
None	7	4.8 (0.9–10.8)	71.6	
**Baseline psIgE** (**kU/L**)				
<60	10	2.2 (0.2–5.7)	41.0	<0.001
≥60	14	10.0 (7.8–12.5)	0.00	
**Baseline SPT** (**mm**)				
<12	11	7.7 (5.1–10.7)	36.9	0.422
≥12	9	4.0 (0.3–10.0)	46.3	

Abbreviations: psIgE, peanut specific IgE; SPT, skin prick test; NA, not applicable.

### Adverse events requiring treatment with medications

**Figure 2:**
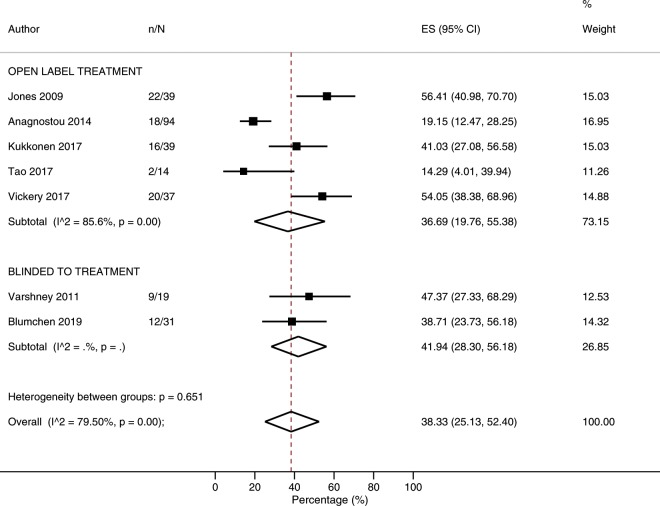
Proportion of participants experiencing adverse events requiring treatment.

The overall risk of an adverse event requiring treatment was 38.3% (25.1–52.4%; I^2^ = 79.5%; N = 7 studies, Fig. 2). In subgroup analyses, risk of an adverse event requiring treatment was higher among studies which included a rush phase (53.7%; 43.4–63.9) compared with studies which commenced with a slow build-up phase (28.1%; 15.7–42.4%; p = 0.005) (Table 3*,* S5 Figure), while frequency of adverse events requiring treatment was lower among studies which included co-treatment alongside POIT (9.2 per 10,000 doses; 3.3–17.9) compared with studies which used POIT alone (19.4 per 10,000 doses; 17.3–21.6; p = 0.027) (S6 Table*,* S6 Figure). Following univariate meta-regression, increasing median age was associated with reduced risk of adverse events requiring treatment (p < 0.001) (S7 Table*,* S7 Figure), but did not appear to influence the frequency (S8 Table*,* S8 Figure).

**Table 3:** Risk of different types of adverse events according to study characteristics.

Comparison	Risk of Adverse Event Requiring Treatment				Risk of Adverse Event Requiring Epinephrine			
	Studies	% (95% CI)	I^2^	p value	Studies	% (95% CI)	I^2^	p value
**Overall**								
	7	38.3 (25.1–52.4)	79.5	NA	26	7.6 (4.5–11.4)	75.5	NA
**Rush Phase**								
Yes	3	53.7 (43.4–63.9)	NA	0.005	17	11.6 (8.1–15.6)	57.8	0.001
No	4	28.1 (15.7–42.4)	68.9		9	2.3 (0.1–6.1)	53.2	
**Co-Treatment**								
Yes	1	41 (27.1–56.6)	NA	NA	7	6.6 (1.9–13.2)	52.7	0.677
No	6	37.8 (22.4–54.5)	82.6		20	8.0 (4.3–12.5)	77.4	
**Intervention Blinding**								
Yes	2	41.9 (28.3–56.2)	NA	0.651	6	7.3 (2.5–13.7)	59.4	0.825
No	5	36.7 (19.8–55.4)	85.6		20	7.9 (3.9–12.8)	78.8	
**Maintenance Dose** (**mg/day**)								
<1000	5	40.5 (24.6–57.5)	82.7	0.880	13	4.0 (1.1–8.2)	74.8	0.001
≥1000	3	38.0 (16.2–62.3)	NA		14	13.7 (9.6–18.3)	37.7	
**Entry OFC**								
DBPCFC	2	24.9 (17.8–32.8)	NA	0.001	13	6.8 (3.2–11.4)	63.7	0.784
Open	3	37.1 (17.7–58.6)	NA		6	6.1 (0.2–16.7)	79.1	
None	2	53.5 (40.3–66.5)	NA		7	10.3 (3.2–20.2)	82.8	
**Baseline psIgE** (**kU/L**)								
<60	3	28.6 (7.9–55.1)	NA	0.215	10	2.8 (0.4–6.5)	43.7	0.018
≥60	4	46.1 (37.3–55.0)	0		14	9.1 (5.7–13.2)	46.0	
**Baseline SPT** (**mm**)								
<12	5	38.6 (24.0–54.2)	79.2	NA	11	7.1 (3.3–12.1)	76.4	0.548
≥12	1	14.3 (4.0–39.9)	NA		8	8.3 (3.3–14.9)	31.4	

Abbreviations: OFC, oral food challenge; DBPCFC, double blind placebo controlled food challenge; psIgE, peanut specific IgE; SPT, skin prick test.

The risk of an adverse event requiring treatment was slightly higher across rush (41.5%; 23.1–61.0%: I^2^ = 72.0%, N = 4 studies), and build-up (43.7%; 33.3–54.5%: I^2^ = 0%, N = 3 studies) treatment phases compared with maintenance (20.9%; 9.7–34.7%: I^2^ = NA%, N = 2 studies) phase (S9 Figure). Similarly, the frequency of an adverse event requiring treatment appeared highest during the rush (116.7/1000 doses; 50.7–204.6/1000 doses: I^2^ = 93.9%, N = 4 studies) phase, compared with build-up (31.3/1000 doses; 2.5–88.5/1000 doses: I^2^ = 99.0%, N = 3 studies) and maintenance (18.2/1000 doses; 10.6–27.8/1000 doses: I^2^ = 97.9%, N = 9 studies) treatment phases, but these findings were subject to substantial heterogeneity (S10 Figure).

### Adverse events requiring treatment with epinephrine

**Figure 3:**
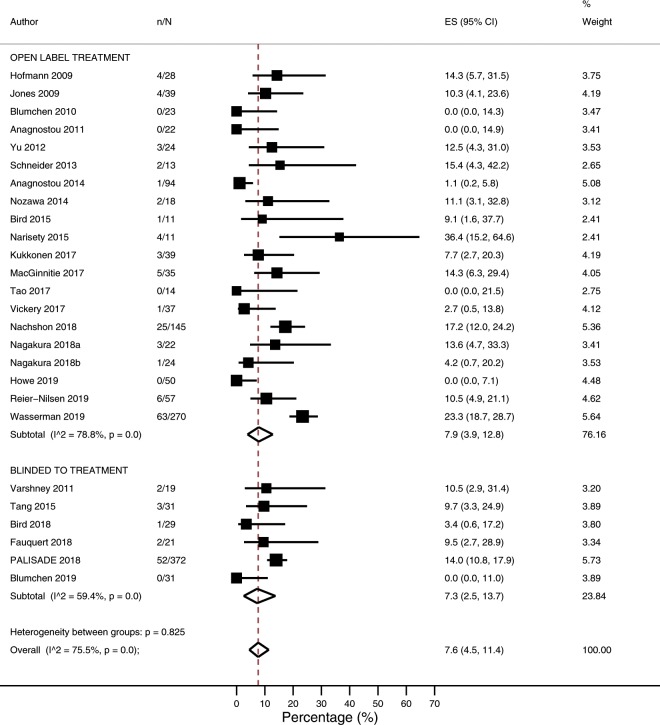
Proportion of participants experiencing adverse events requiring treatment with epinephrine.

The overall risk of an adverse event requiring treatment with epinephrine was 7.6% (4.5–11.4 I^2^ = 75.5; N = 26 studies, Fig. 3), while the overall frequency of adverse events requiring treatment with epinephrine was 0.20 per 10,000 doses (0.08–0.37; I^2^ = 64.4%; N = 15 studies). In subgroup analyses, risk of an adverse event requiring treatment with epinephrine was higher among studies which included a rush phase (11.6%; 8.1–15.6%) compared with studies which commenced with a slow build-up phase (2.3%; 0.1–6.1%; p = 0.001) and was higher among studies employing a target maintenance dose ≥1000 mg (13.7%; 9.6–18.3%) compared with <1000 mg (4.0%; 1.1–8.2%; p = 0.001) (Table 3*,* S11 Figure). Similar findings were evident regarding frequency of adverse events requiring treatment with epinephrine (S6 Table, S12 Figure). Following univariate meta-regression, an increased risk of adverse events requiring treatment with epinephrine was associated with increasing median baseline peanut specific IgE (p = 0.0247) (S7 Table*,* S13 Figure), while a higher frequency of adverse events requiring treatment with epinephrine was associated with a higher target maintenance dose and higher baseline SPT (p = 0.0243) (S8 Table*,* S14 Figure).

The risk of an adverse event requiring treatment with epinephrine appeared similar across rush (4.1%; 0.7–9.3%: I^2^ = 51.3%, N = 9 studies), build-up (3.2%; 0.0–9.5%: I^2^ = 84.8%, N = 13 studies) and maintenance (3.2%; 0.9–6.4%: I^2^ = 53.9%, N = 15 studies) treatment phases (S9 Figure). In contrast, the frequency of an adverse event requiring treatment with epinephrine was highest during the rush (2.63/1000 doses; 0.00–11.73/1000 doses: I^2^ = 64.7%, N = 3 studies) treatment phase, compared with build-up (0.00/1000 doses; 0.00–0.20/1000 doses: I^2^ = 0%, N = 7 studies) and maintenance (0.09/1000 doses; 0.00–0.34/1000 doses: I^2^ = 37.1%, N = 7 studies) treatment phases (S10 Figure).

### Likelihood of reaching target maintenance dose

**Figure 4:**
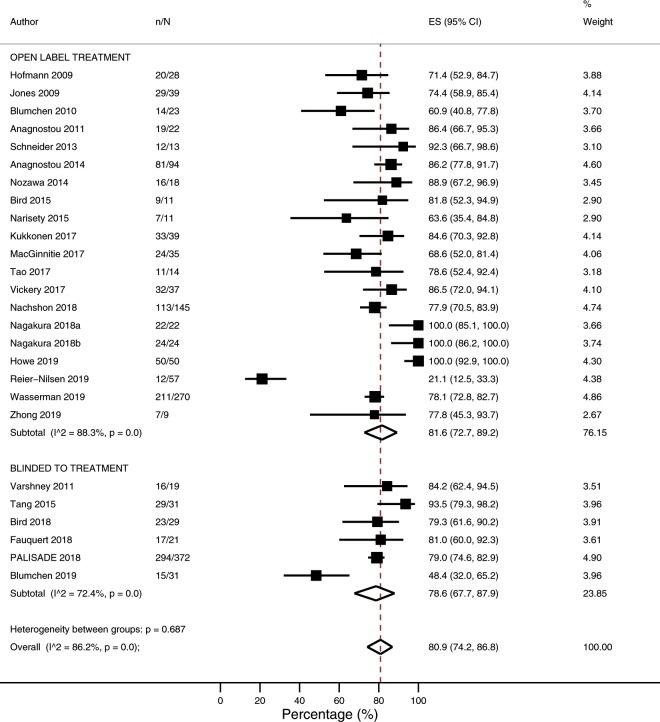
Proportion of participants able to reach target maintenance dose.

The overall likelihood of reaching target maintenance dose was 80.9% (95% CI 74.2–86.8%; I^2^ = 86.2%; N = 26 studies, Fig. 4). In subgroup analyses, the proportion reaching target maintenance dose was higher among studies which included co-treatment alongside POIT (95.0%; 87.6–99.6%) compared with studies which used POIT alone (72.7%; 64.7–80.0%; p = < 0.001) (S9 Table*,* S15 Figure). When stratified according to co-treatment type, an increased likelihood of reaching the target maintenance dose was noted irrespective of the type of co-treatment administered (S9 Table*,* S15 Figure). Following univariate meta-regression, a low likelihood of reaching the target maintenance dose was associated with a higher target maintenance dose (p = 0.0088) (S10 Table*,* S16 Figure).

### Likelihood of passing supervised exit OFC

**Figure 5:**
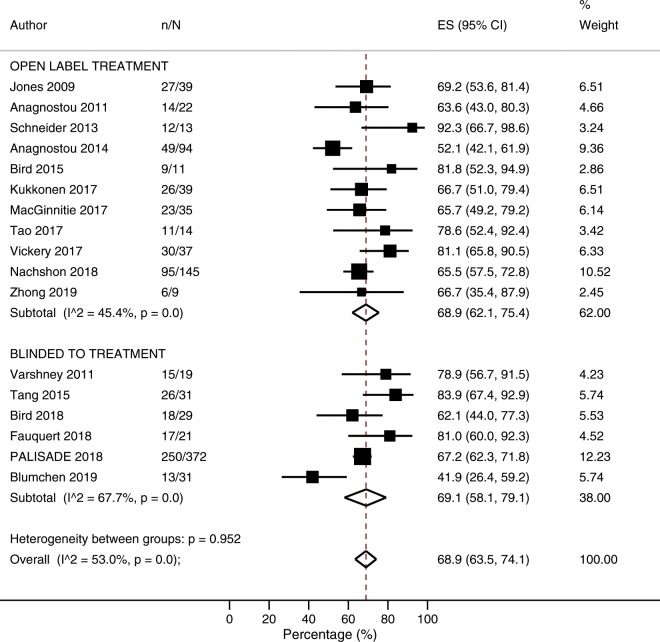
Proportion of participants able to complete peanut oral immunotherapy and pass supervised exit oral food challenge.

The overall likelihood of reaching the end of the study and passing a supervised exit oral food challenge was 68.9% (63.5–74.1%; I^2^ = 53.0%; N = 17 studies, Fig. 5). In subgroup analyses, the likelihood of passing a supervised food challenge was higher among studies which included co-treatment alongside POIT (78.7%; 68.8–87.3%) compared with studies which used POIT alone (65.6%; 58.5–72.3%; p = 0.035, S9 Table*,* S17 Figure). When stratified according to co-treatment type, an increased likelihood of passing a supervised food challenge was noted among studies that administered probiotics or omalizumab, but not antihistamines (S9 Table*,* S17 Figure). Following univariate meta-regression, a higher likelihood of passing a supervised food challenge was associated with higher baseline SPT (p = 0.0058) (S10 Table*,* S18 Figure). In a sensitivity per-protocol analysis, the overall likelihood of passing a supervised oral food challenge and the end of POIT treatment among those able to tolerate the maintenance dose was 88.8% (83.2–93.5%; I^2^ = 68.2) (S19 Figure).

## Discussion

This systematic review and meta-analysis of almost 1,500 peanut allergic patients who received POIT among 27 controlled and non-controlled studies quantifies the risk and frequency of serious adverse events associated with treatment. While POIT appears effective in achieving the immunological goal of desensitisation in large proportion of subjects, serious adverse events lead to treatment failure in 1 in 15 subjects. Further, potentially life-threatening reactions requiring treatment with epinephrine are experienced by approximately 1 in 13 subjects throughout all stages of treatment. While the risk of potentially life-threatening reactions appeared similar across treatment phases, the frequency of epinephrine use was low once subjects reached the long-term treatment phase. Most notably, we identified modifiable treatment protocol related factors such as the elimination of a rush phase, aiming for a lower target maintenance dose, or use of co-treatments in addition to POIT, that could substantially improve the safety and efficacy of treatment regimens and warrant evaluation in future clinical trials.

Our study findings significantly expand upon those of the recent meta-analysis published by Chu *et al*.^12^, through including an additional 15 studies and data on more than twice the number of participants receiving POIT. Despite differences in inclusion of controlled and non-controlled studies, Chu *et al*. observed an absolute risk of adverse events resulting in treatment discontinuation of 6.1% (2.9–12.9) compared to our observed risk of 6.6% (4.4–9.0%). Similarly, our observed risk of epinephrine use of 7.6% (4.5–11.4%) was similar to the risk of 8.2% (4.7–14.2%) reported by Chu *et al*. A major point of difference to the previous meta-analysis by Chu *et al*. was the extraction of data pertaining to the frequency of adverse events. Examination of these data revealed that, while the risk of serious adverse events to POIT may appear high at up to 39.4%, the frequency with which they occur is quite low, with 11.3 per 1,000 doses (5.4–19.5) resulting in adverse events requiring medication therapy and 0.2 per 1,000 doses (0.08–0.37) resulting in epinephrine use. Lastly, in their summary of findings table Chu *et al*. report an observed anticipated absolute treatment effect of passing a supervised exit OFC following POIT of 39.7% (21.8–72.3%), based on the corresponding risk ratio of 12.42 (6.82–22.61) generated from their meta-analysis^12^. This substantially differs from the reported prevalence of passing a supervised exit OFC following POIT of 56% (n = 320/574)^12^, based on the crude data included in their-meta-analysis. A second issue is their inclusion of an unpublished study (NCT01324401 [PNOIT]; 2018) that performed the exit OFC one month after treatment avoidance. This study provided the lowest risk estimate for passing a supervised exit OFC (RR 3.30; 0.60–18.23) and therefore lowered the overall risk estimate, but was not eligible for inclusion as it was evaluating sustained unresponsiveness. In contrast, our meta-analysis demonstrated a higher overall pooled likelihood of passing a supervised exit OFC of 69.1% (58.1–79.1%) among blinded RCTs and 68.9% (62.1–75.4%) among open-label studies. Determining the true expected treatment effect should be the focus of future studies as this is likely to be a key factor influencing decision making regarding POIT.

Despite investigating more than 10 study-level factors in sub-group analyses, Chu *et al*. identified that only the requirement for failing an entry OFC as an entry criterion was associated with an increased risk of anaphylaxis during POIT^12^. The inability to detect additional associations is likely the result of inadequate statistical power. This is in contrast to the novel findings of our meta-analysis where use of an initial rush phase was consistently associated with an increased risk of serious adverse events, while aiming for a higher target maintenance dose was associated with an increased risk for epinephrine use. Further, use of co-treatment in addition to POIT was associated with a reduction in adverse events resulting in treatment discontinuation.

Only three RCTs have been undertaken to examine the effects of different approaches to POIT. These include different target maintenance doses (300 mg or 3000 mg peanut protein daily)^42^, use of co-treatment (omalizumab vs. placebo)^30^, or changing psychological mindset regarding symptoms associated with POIT^27^. While these studies, which recruiting between 35 to 50 patients, provided some promising evidence that targeting a lower target maintenance dose, utilising a co-treatment, or providing a psychological intervention may reduce the risk and frequency of adverse events, they were insufficiently powered to detect any clinically significant differences. Notably, we were able to overcome this limitation of relying on single studies by pooling data across studies in the largest meta-analysis undertaken on POIT to date.

Few previous studies have attempted to evaluate patient-level factors associated with the risk of adverse events during POIT, including three trials using individual patient data^37,43,46^ and one meta-analysis^12^. Two studies examined differences in patient characteristics according to frequency of overall adverse events or epinephrine use^43,46^. Findings have been largely inconsistent, with one study demonstrating that rhinitis, asthma, and baseline SPT were all associated with an increased risk of adverse events^46^, while the other demonstrated that higher baseline psIgE was associated with an increased risk of epinephrine use^43^. In the meta-analysis by Chu *et al*., only increasing age was associated with an increased risk of serious adverse events^12^. We observed an increased risk of adverse events among studies with a higher proportion of participants with rhinitis, while a higher proportion of participants with eczema and higher median age were associated with increased risk of adverse events requiring treatment. Further, our meta-analysis provides evidence suggesting that both higher baseline psIgE and SPT may increase the risk of adverse events during POIT, but each of these findings requires clarification in further studies.

Strengths of our meta-analysis include the thorough literature search, inclusion of data from controlled and non-controlled studies, and use of appropriate statistical methods for pooling prevalence data and undertaking meta-regression analyses. Our study also has some limitations. Because this is a study-level analysis, it is not possible to make inferences regarding which individual patients are at higher risk of adverse events related to POIT. Confidence intervals for pooling risk estimates include both between-study and within-study variations, and should be interpreted cautiously. Future studies pooling individual patient data could overcome such limitations. A high level of heterogeneity was observed across some study outcomes, which we attempted to take into account using random effects models, and explored through performing subgroup comparisons. An additional limitation was the incomplete reporting of data on some outcomes or study-level characteristics across studies. For example, less than half of included studies separated adverse event data according to treatment phase. Further, definitions of adverse events varied across studies. Some studies reported adverse events regardless of severity, while others restricted reporting to those considered to be moderate or severe. Greater efforts should be made to standardise reporting of adverse events in future clinical trials to facilitate pooling of data. Lastly, in some situations the study protocol was modified part way during the study^31,35,43^, with outcomes data reported for participants overall, rather than separated according to use of different treatment protocols.

While the risk of severe adverse reactions requiring treatment with epinephrine might appear high at 7.6%, this must be balanced against the benefits of POIT in providing protection against accidental peanut ingestion. A longitudinal study of a population of children who had developed peanut allergy before the age of 4 years revealed that, of the children with initial non-life-threatening reactions, 44% had at least one potentially life-threatening reaction during follow-up^47^. Based on this meta-analysis, once children reached the long-term maintenance phase of POIT, the risk of experiencing a significant adverse event requiring treatment with epinephrine was 3.2%, while the frequency was extremely low at 9 episodes per 100,000 doses.

Our findings are novel and identify modifiable study protocol related factors that can guide development of future treatment protocols that are safer and more effective. Given the current evidence that OIT is superior to placebo in achieving the immunological goal of desensitisation, future clinical trials should be focused on improving treatment protocols with the aim of improving safety, while maintaining high levels of efficacy. While expense and administration-related challenges associated with the use of omalizumab as a co-treatment may prohibit widespread adoption in clinical practice, we observed evidence that lower cost co-treatments such as use of oral antihistamines or probiotics were also associated with improved treatment-related outcomes. The specific value of these co-treatments should be evaluated in appropriate clinical trials. Further, in situations where POIT is currently offered to patients, our study provides significant information regarding not only the risk, but also frequency, of adverse events and related outcomes.

Lastly, an interesting observation relating to various treatment protocols was the inclusion of various recommendations made regarding dosing restrictions aiming to minimise the risk of potential adverse events. For example, some studies recommend avoiding exercise within 3 hours of dosing^36^, avoiding a hot shower or bath for up to 4 hours following dosing^22^, or avoiding dosing during episodes of illness such as respiratory infections^41^, or during menstruation^22^. Notably, no studies evaluated patient adherence to such treatment recommendations. Further, reporting of potential attenuating risk factors at the time of adverse event occurrence is very limited and inconsistent across studies. This means that based on included studies it is not possible to evaluate whether adherence to such treatment recommendations actually alters treatment outcomes. Given the lack of high-quality data regarding the relationship between such factors and the occurrence of adverse events, as well as underlying biological mechanisms, such recommendations should be the focus of future research.

In conclusion, while the majority of children undergoing POIT are able to be effectively desensitised and are protected from accidental peanut ingestion, this study provides promising evidence that certain modifications to existing treatment protocols could significantly improve treatment safety and efficacy. In particular, modifications to POIT treatment protocols, such as the avoidance of a rush phase, lower target maintenance doses, or use of co-treatments, warrant appropriate evaluation in future clinical trials, as well as further studies identifying those at greatest risk of experiencing serious adverse events.

## Supplementary information

Supplemental Files.

Dataset 1.

## Data Availability

All data generated and analysed during the study are included in this published article.
