# Supplementary material for: Adverse events associated with peanut oral immunotherapy in children – a systematic review and meta-analysis
Source: Sci Rep. 2020 Jan 20;10:659. doi: 10.1038/s41598-019-56961-3 (PMC6971009; doi:10.1038/s41598-019-56961-3)
Supplement: Supplementary file 1 — Supplemental Files. [file 41598_2019_56961_MOESM1_ESM.docx]

**Supplemental Files:**

**Title: Adverse events associated with peanut oral immunotherapy in children – a systematic review and meta-analysis**

**Authors: Grzeskowiak LE, Tao B, Knight E, Cohen-Woods S, Chataway T**

Contents

[S1 Table. PRISMA checklist for systematic reviews and meta-analyses 3](#_Toc17552364)

[S1 Appendix. Summary of search strategy 5](#_Toc17552365)

[S1 Figure. PRISMA search flow diagram 6](#_Toc17552366)

[S2 Figure. Funnel plots 7](#_Toc17552367)

[S3 Figure. Forest plots - Adverse Event Causing Treatment Discontinuation 11](#_Toc17552368)

[S4 Figure. Meta-regression bubble plots - Adverse Event Causing Treatment Discontinuation 16](#_Toc17552369)

[S5 Figure. Forest plots - Risk of Adverse Events Requiring Treatment 17](#_Toc17552370)

[S6 Figure. Forest plots - Frequency of Adverse Events Requiring Treatment 21](#_Toc17552371)

[S7 Figure. Meta-regression bubble plots - risk of adverse events requiring medication treatment 25](#_Toc17552372)

[S8 Figure. Meta-regression bubble plots - Frequency of Adverse Events Requiring Medication Treatment 26](#_Toc17552373)

[S9 Figure. Risk of Adverse Events According to Treatment Phase 27](#_Toc17552374)

[S10 Figure. Frequency of Adverse Events According to Treatment Phase 28](#_Toc17552375)

[28](#_Toc17552376)

[S11 Figure. Forest plots - Risk of Adverse Events Requiring Treatment with Epinephrine 29](#_Toc17552377)

[S12 Figure. Forest plots - Frequency of Adverse Events Requiring Treatment with Epinephrine 35](#_Toc17552378)

[S13 Figure. Meta-regression bubble plots - Risk of Adverse Events Requiring Treatment with Epinephrine 39](#_Toc17552379)

[S14 Figure. Meta-regression bubble plots - Frequency of Adverse Events Requiring Treatment with Epinephrine 40](#_Toc17552380)

[S15 Figure. Forest plots - Able to Reach Target Maintenance Dose 41](#_Toc17552381)

[S16 Figure. Meta-regression bubble plots - Able to Reach Target Maintenance Dose 45](#_Toc17552382)

[S17 Figure. Forest plots - Pass Supervised Exit Oral Food Challenge 46](#_Toc17552383)

[S18 Figure. Meta-regression bubble plots - Completion of Supervised Oral Food Challenge 50](#_Toc17552384)

[S19 Figure. Forest plot for successful completion of supervised exit oral food challenge according to per-protocol analysis 51](#_Toc17552385)

[S2 Table. Summary of characteristics of included studies 52](#_Toc17552386)

[S3 Table. Comparison of characteristics between controlled and non-controlled studies 53](#_Toc17552387)

[S4 Table. Additional characteristics of included peanut oral immunotherapy studies 54](#_Toc17552388)

[S5 Table. Meta-regression of the risk of adverse event causing treatment discontinuation according to study level characteristics 56](#_Toc17552389)

[S6 Table. Frequency of different types of adverse events according to study characteristics 57](#_Toc17552390)

[S7 Table. Meta-regression of the risk of different types of adverse events according to study level characteristics 58](#_Toc17552391)

[S8 Table. Meta-regression of the frequency of different types of adverse events according to study level characteristics 59](#_Toc17552392)

[S9 Table. Pooled proportion of participants able to reach the target maintenance dose or pass supervised exit oral food challenge according to study characteristics 60](#_Toc17552393)

[S10 Table. Meta-regression of the pooled proportion of participants able to reach the target maintenance dose or pass supervised exit oral food challenge according to study level characteristics 61](#_Toc17552394)

| S1 Table. PRISMA checklist for systematic reviews and meta-analyses | | | | |
| --- | --- | --- | --- | --- |
| **Section/topic** | **#** | **Checklist item** | **Reported in manuscript section/paragraph number** | |
| **TITLE** | | | |  |
| Title | 1 | Identify the report as a systematic review, meta-analysis, or both. | Title | |
| **ABSTRACT** | | | |  |
| Structured summary | 2 | Provide a structured summary including, as applicable: background; objectives; data sources; study eligibility criteria, participants, and interventions; study appraisal and synthesis methods; results; limitations; conclusions and implications of key findings; systematic review registration number. | Abstract | |
| **INTRODUCTION** | | | |  |
| Rationale | 3 | Describe the rationale for the review in the context of what is already known. | Introduction/1-4 | |
| Objectives | 4 | Provide an explicit statement of questions being addressed with reference to participants, interventions, comparisons, outcomes, and study design (PICOS). | Introduction/4 | |
| **METHODS** | | | |  |
| Protocol and registration | 5 | Indicate if a review protocol exists, if and where it can be accessed (e.g., Web address), and, if available, provide registration information including registration number. | Methods/1 | |
| Eligibility criteria | 6 | Specify study characteristics (e.g., PICOS, length of follow-up) and report characteristics (e.g., years considered, language, publication status) used as criteria for eligibility, giving rationale. | Methods/2 | |
| Information sources | 7 | Describe all information sources (e.g., databases with dates of coverage, contact with study authors to identify additional studies) in the search and date last searched. | Methods/1 | |
| Search | 8 | Present full electronic search strategy for at least one database, including any limits used, such that it could be repeated. | S1 Appendix | |
| Study selection | 9 | State the process for selecting studies (i.e., screening, eligibility, included in systematic review, and, if applicable, included in the meta-analysis). | Methods/2 | |
| Data collection process | 10 | Describe method of data extraction from reports (e.g., piloted forms, independently, in duplicate) and any processes for obtaining and confirming data from investigators. | Methods/3-4 | |
| Data items | 11 | List and define all variables for which data were sought (e.g., PICOS, funding sources) and any assumptions and simplifications made. | Methods/4 | |
| Risk of bias in individual studies | 12 | Describe methods used for assessing risk of bias of individual studies (including specification of whether this was done at the study or outcome level), and how this information is to be used in any data synthesis. | Methods/4 | |
| Summary measures | 13 | State the principal summary measures (e.g., risk ratio, difference in means). | Methods/5 | |
| Synthesis of results | 14 | Describe the methods of handling data and combining results of studies, if done, including measures of consistency (e.g., I^2^) for each meta-analysis. |  | |

| Risk of bias across studies | 15 | Specify any assessment of risk of bias that may affect the cumulative evidence (e.g., publication bias, selective reporting within studies). | Methods/5 | |
| --- | --- | --- | --- | --- |
| Additional analyses | 16 | Describe methods of additional analyses (e.g., sensitivity or subgroup analyses, meta-regression), if done, indicating which were pre-specified. | Methods/6 | |
| **RESULTS** | | | |  |
| Study selection | 17 | Give numbers of studies screened, assessed for eligibility, and included in the review, with reasons for exclusions at each stage, ideally with a flow diagram. | Results/1; S1 Figure | |
| Study characteristics | 18 | For each study, present characteristics for which data were extracted (e.g., study size, PICOS, follow-up period) and provide the citations. | Table 1; S4 Table | |
| Risk of bias within studies | 19 | Present data on risk of bias of each study and, if available, any outcome level assessment (see item 12). | Not reported individually | |
| Results of individual studies | 20 | For all outcomes considered (benefits or harms), present, for each study: (a) simple summary data for each intervention group (b) effect estimates and confidence intervals, ideally with a forest plot. | Fig 1-4; S3, 5, 7, 9, 10, 13, 14, 17, 19 | |
| Synthesis of results | 21 | Present results of each meta-analysis done, including confidence intervals and measures of consistency. | Fig 1-4; S3, 5, 7, 9, 10, 13, 14, 17, 19 | |
| Risk of bias across studies | 22 | Present results of any assessment of risk of bias across studies (see Item 15). | Fig 1-4; S3, 5, 7, 9, 10, 13, 14, 17, 19 | |
| Additional analysis | 23 | Give results of additional analyses, if done (e.g., sensitivity or subgroup analyses, meta-regression [see Item 16]). | Results/ Table S5, 6, 7, 8, 9, 10, 11 | |
| **DISCUSSION** | | | |  |
| Summary of evidence | 24 | Summarize the main findings including the strength of evidence for each main outcome; consider their relevance to key groups (e.g., healthcare providers, users, and policy makers). | Discussion/1 | |
| Limitations | 25 | Discuss limitations at study and outcome level (e.g., risk of bias), and at review-level (e.g., incomplete retrieval of identified research, reporting bias). | Discussion/ 10-11 | |
| Conclusions | 26 | Provide a general interpretation of the results in the context of other evidence, and implications for future research. | Conclusion/1 | |
| **FUNDING** | | | |  |
| Funding | 27 | Describe sources of funding for the systematic review and other support (e.g., supply of data); role of funders for the systematic review. |  | |

*From:*  Moher D, Liberati A, Tetzlaff J, Altman DG, The PRISMA Group (2009). Preferred Reporting Items for Systematic Reviews and Meta-Analyses: The PRISMA Statement. PLoS Med 6(7): e1000097. doi:10.1371/journal.pmed1000097

S1 Appendix. Summary of search strategy

**MEDLINE**

1. Peanut Hypersensitivity/
2. Peanut allerg*.mp
3. Peanut-allergic.mp
4. Peanut.mp
5. 1 OR 2 OR 3 OR 4
6. Desensitization, immunologic/
7. Desensiti*.mp
8. Immunotherapy/
9. Immunotherapy.mp
10. 6 OR 7 OR 8 OR 9
11. 5 AND 10

**EMBASE**

1. ‘peanut hypersensitivity’/exp OR ‘peanut allerg*’ OR ‘peanut-allergic’ OR ‘peanut’/exp AND [embase]/lim
2. ‘desensiti*’/exp OR ‘immunotherapy’ AND [embase]/lim
3. #1 AND #2 AND [humans]/lim

**Web of Science**

TS=(“peanut hypersensitivity” OR “peanut allerg*” OR “peanut-allergic” OR peanut) AND TS=("immunotherapy” OR "desensiti*")

S1 Figure. PRISMA search flow diagram

| **Identification** | 2694 records were identified from MEDLINE (352), EMBASE (1157), WEB OF SCIENCE (1185).  1736 records after duplicates removed |
| --- | --- |
| **Screening** | 1736 titles screened  Removed: 1680 on abstract-review, and 23 identified as abstracts of articles already included.  33 titles retrieved for full text review  6 articles excluded for:   - Duplication of data reported elsewhere (4) - Review article (1) - Study protocol (1) |
| **Eligibility** | 27 articles were found eligible |
| **Included** | 27 included in the meta-analysis |

S2 Figure. Funnel plots

**(A) Risk of treatment discontinuation due to adverse events**

Random-Effects Model:

Eggers Test

p=0.3524

**(B) Risk of adverse events requiring treatment with medications**

Random-Effects Model:

Eggers Test

p=0.3398

**(C) Risk of adverse events requiring treatment with epinephrine**

Random-Effects Model:

Eggers Test

p=0.015

**(D) Frequency of doses resulting in treatment with medications**

Random-Effects Model:

Eggers Test

p=0.0457

**(E) Frequency of doses resulting in treatment with epinephrine**

Random-Effects Model:

Eggers Test

p=0.1489

**(F) Likelihood of reaching target maintenance dose**

Random-Effects Model:

Eggers Test

p=0.7811

**(G) Likelihood of reaching the end of the treatment protocol and passing a supervised oral food challenge**

Random-Effects Model:

Eggers Test

p=0.2619

S3 Figure. Forest plots - Adverse Event Causing Treatment Discontinuation

1. Rush Phase


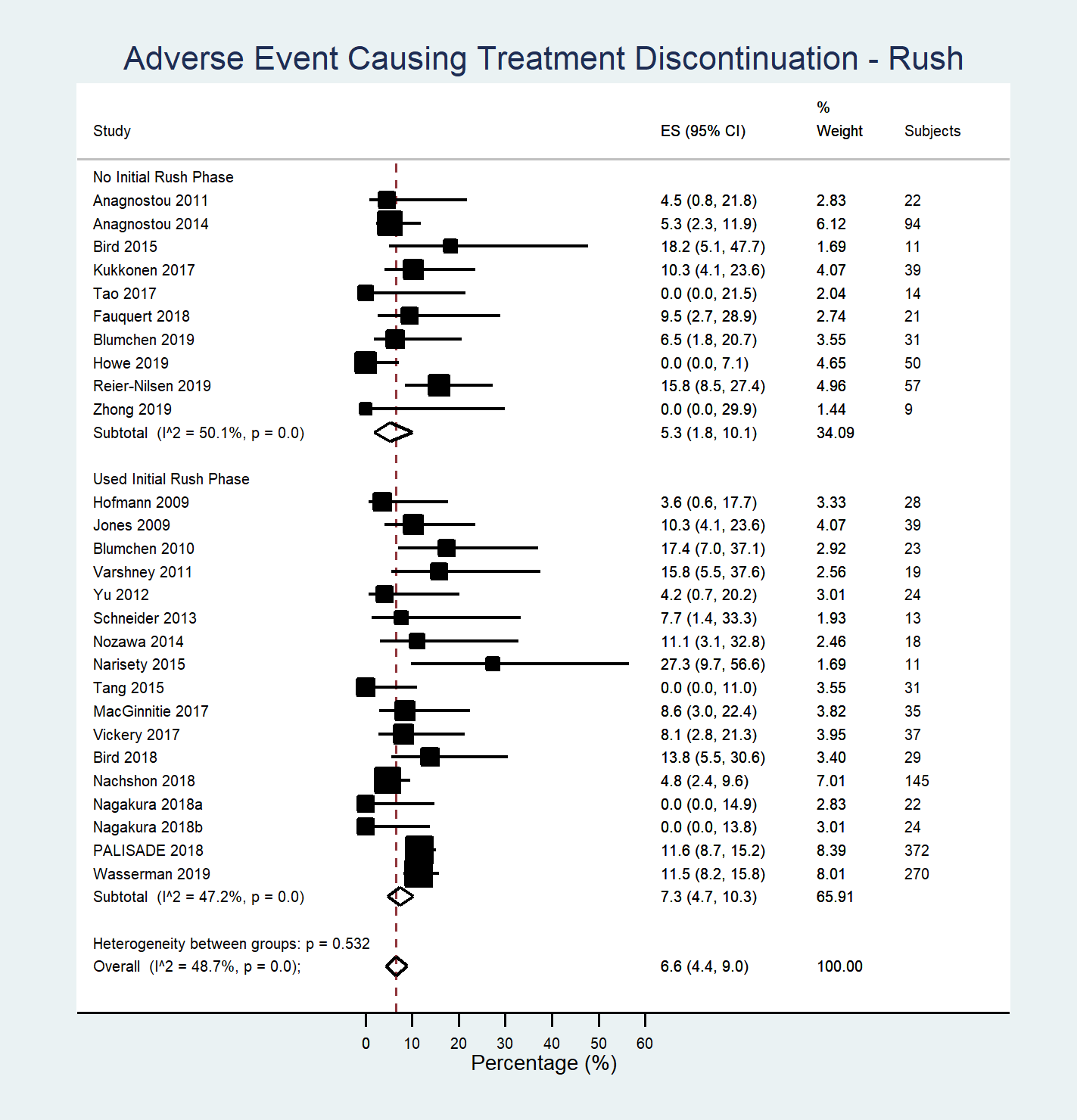


1. Co-Treatment


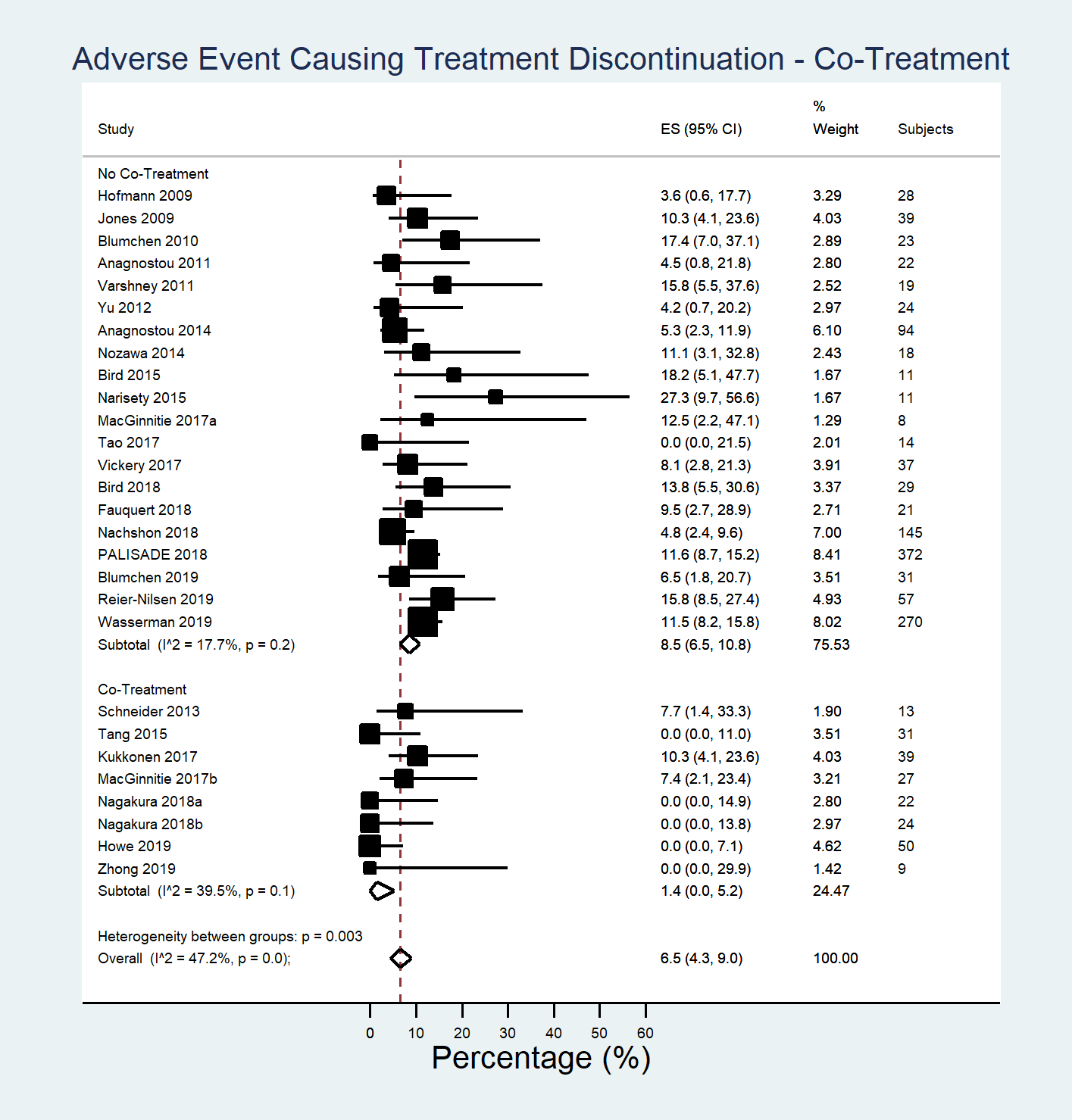


1.
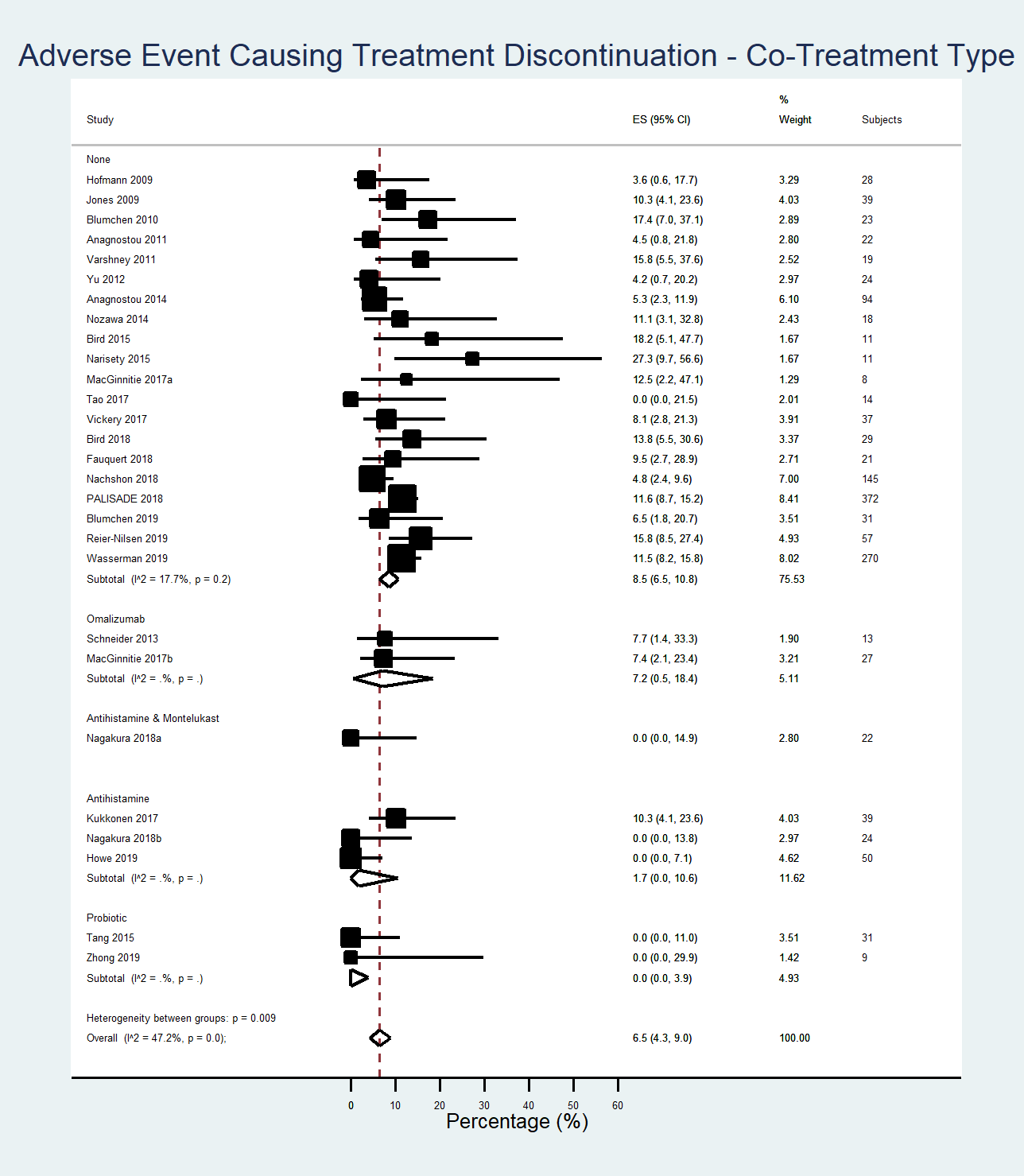
Co-Treatment Type
2. Target Maintenance Dose


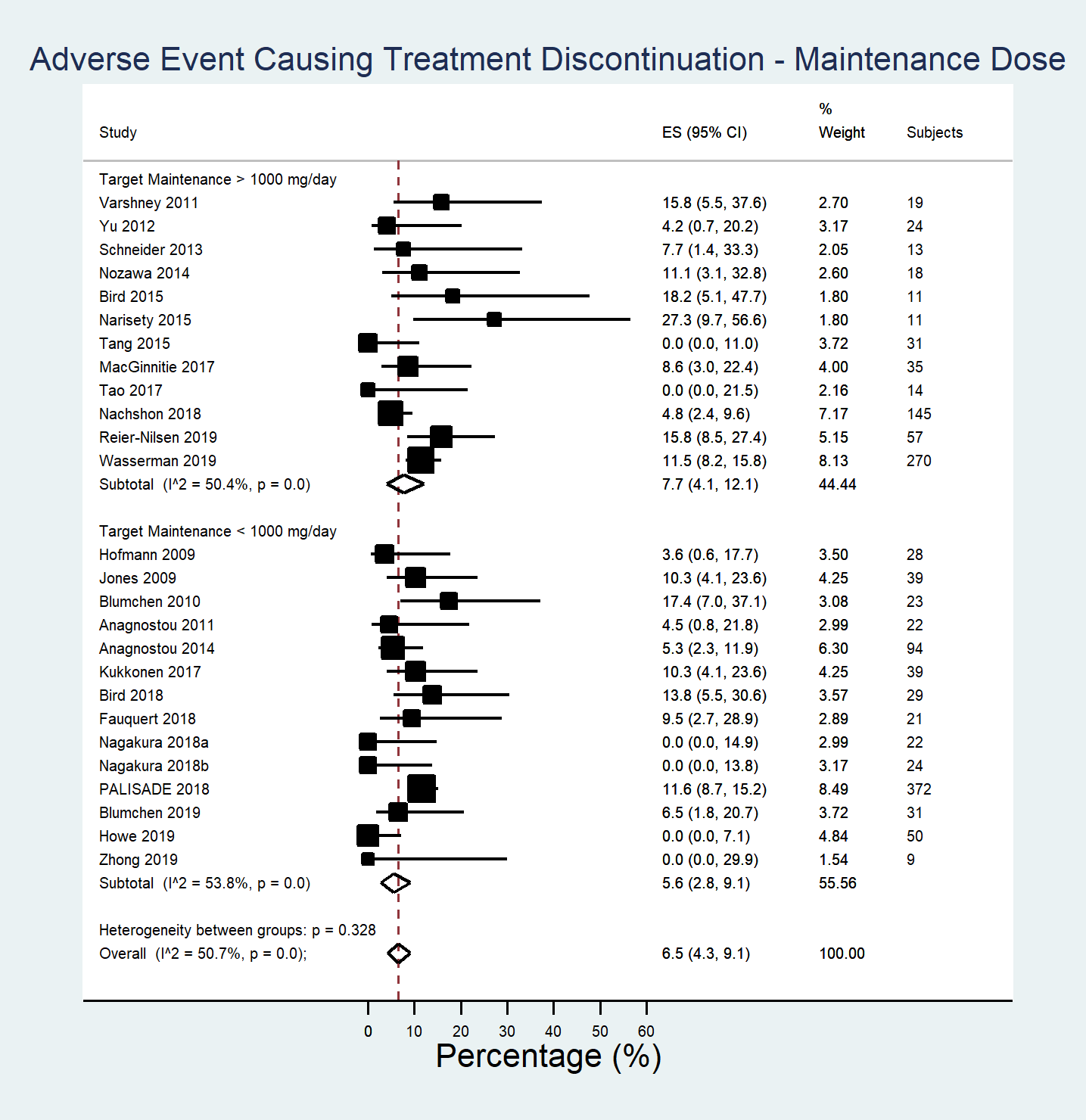


1. Entry Oral Food Challenge


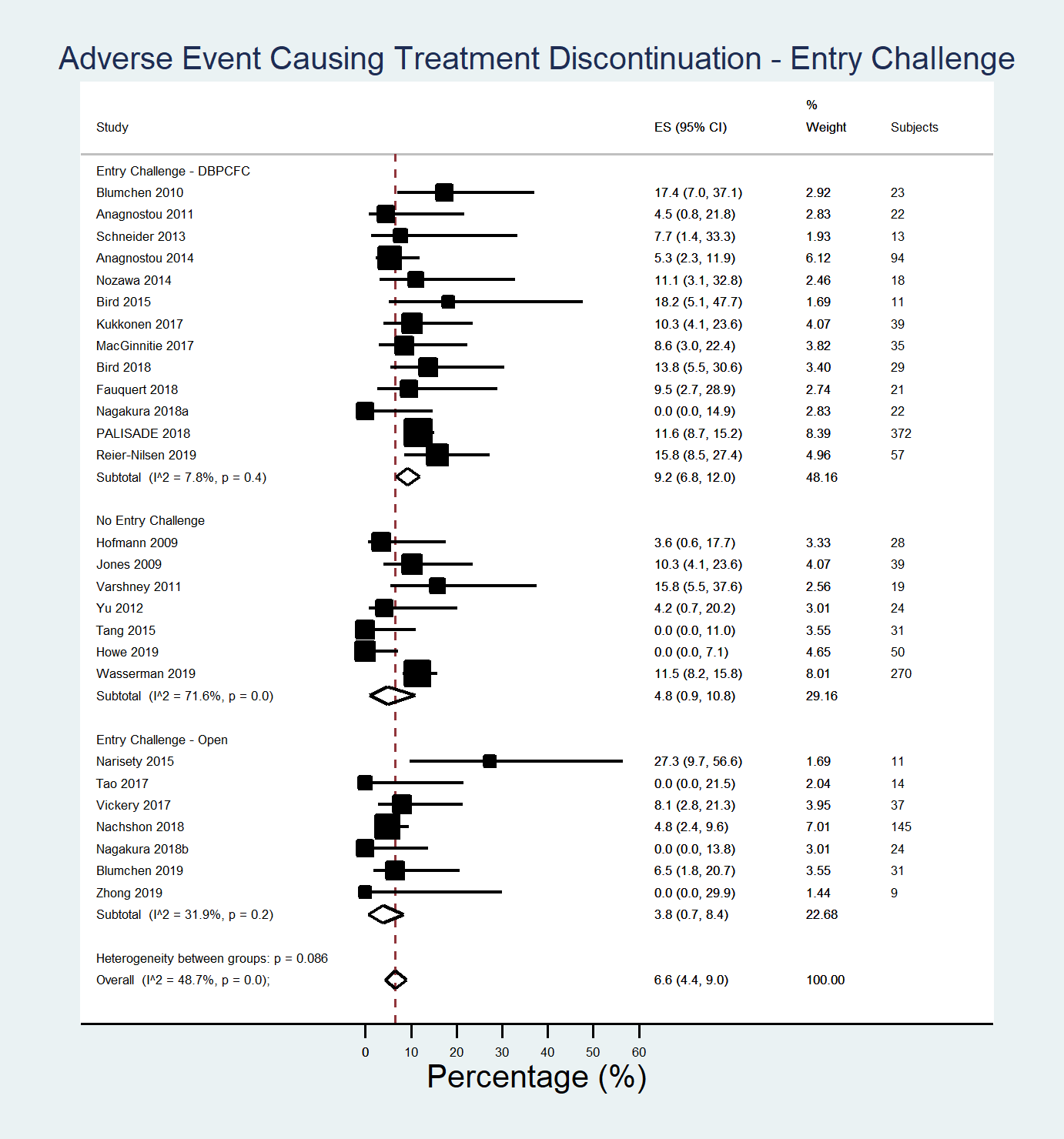


1. Treatment Blinding

1. Baseline Peanut Specific IgE (median)


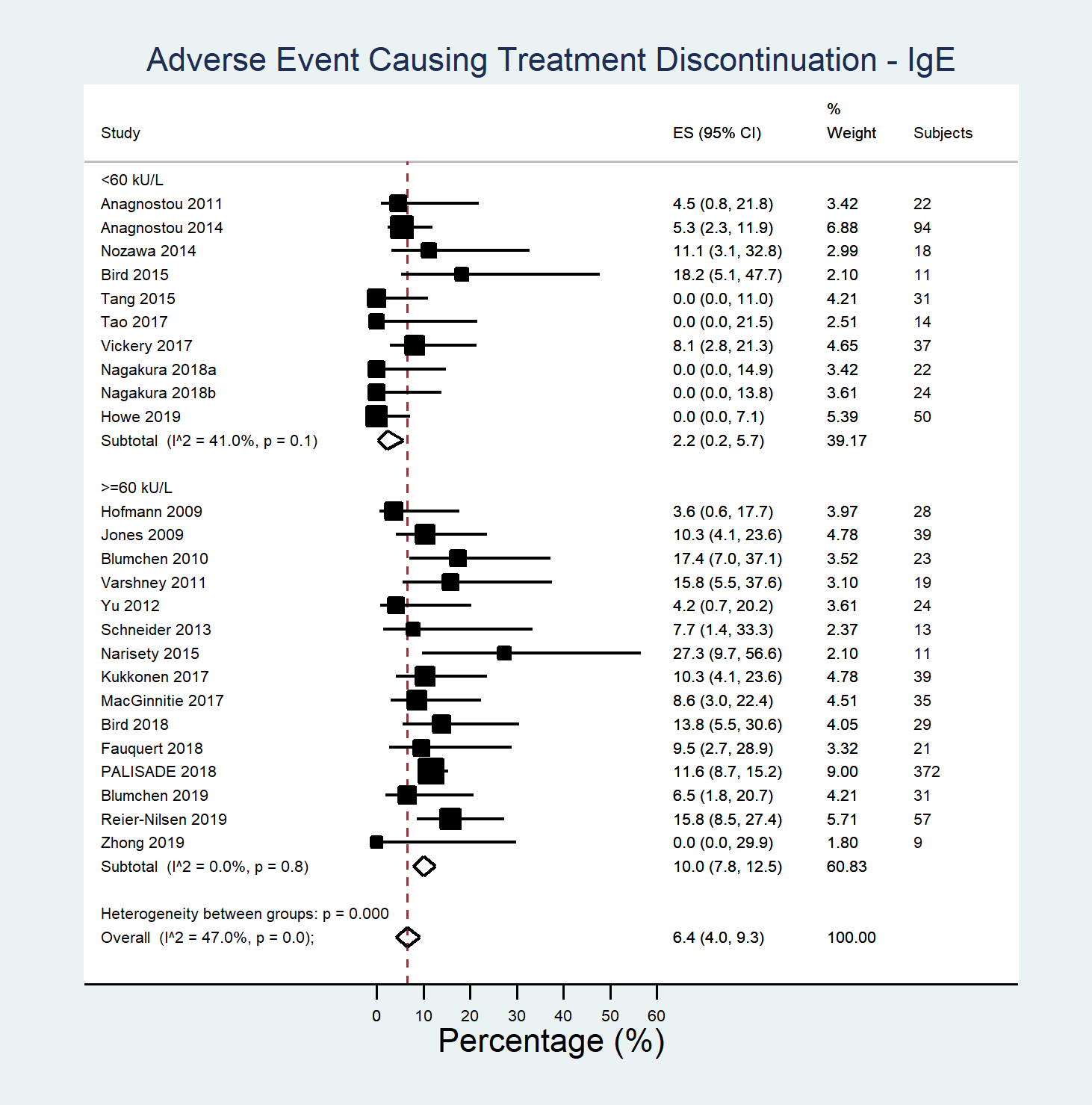


1. Baseline SPT (median)


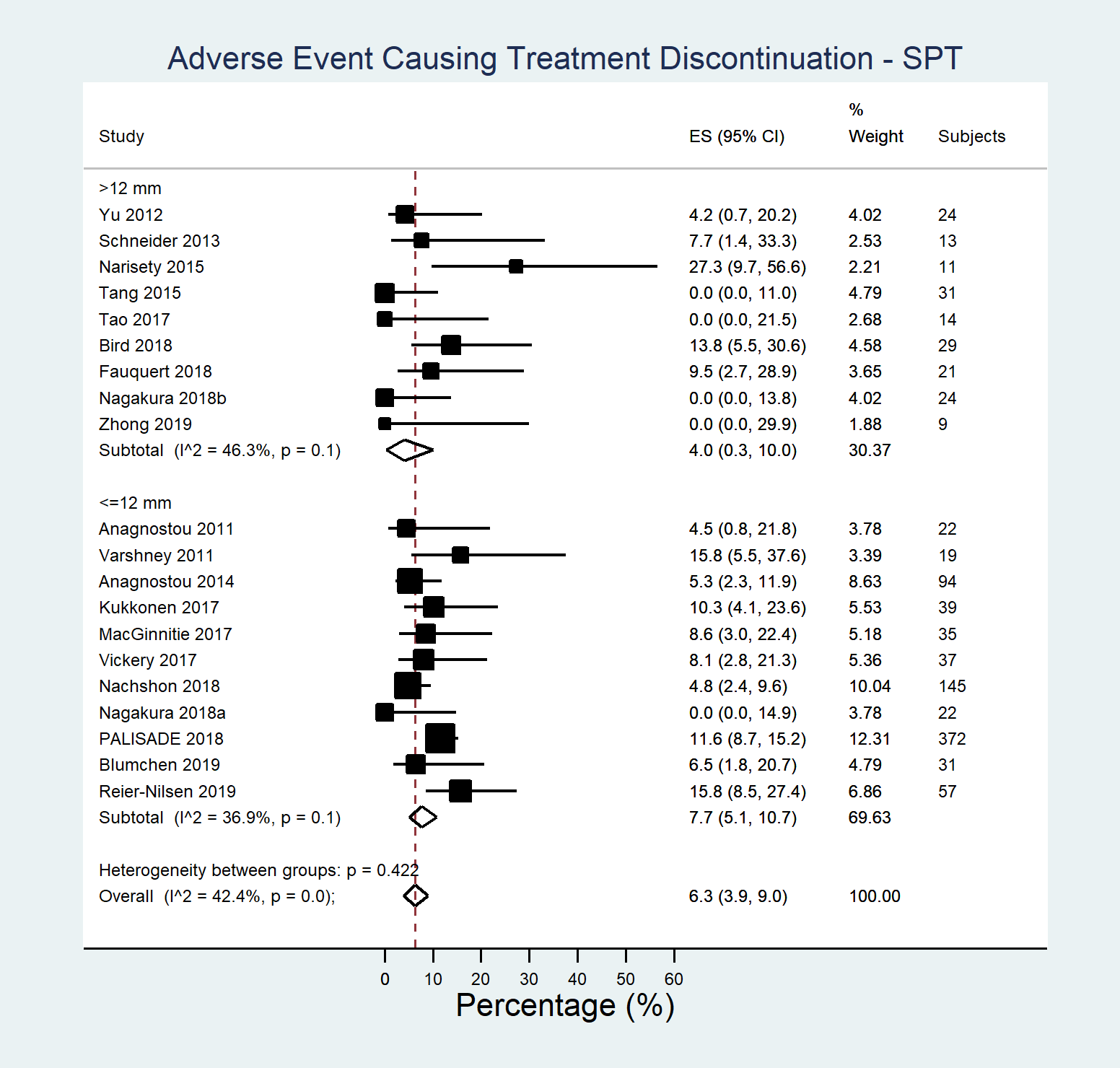


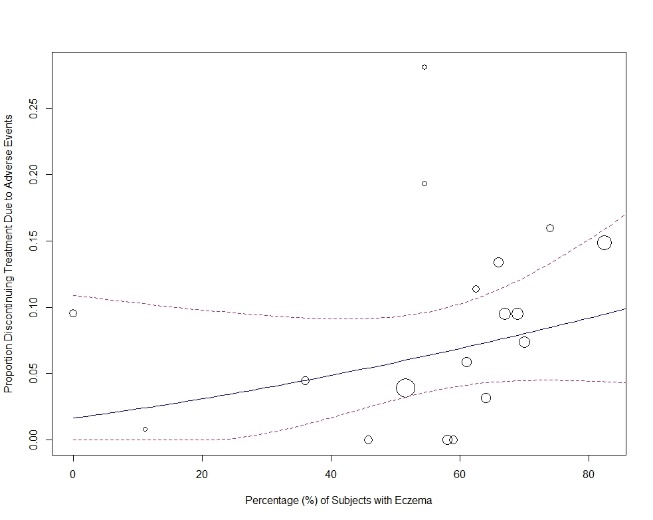

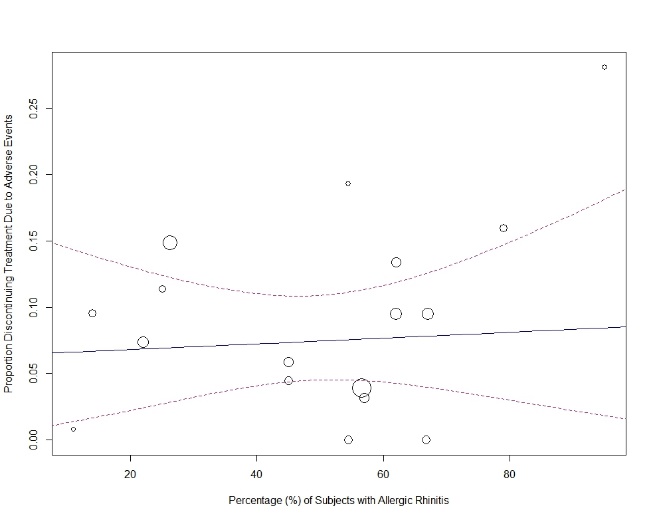

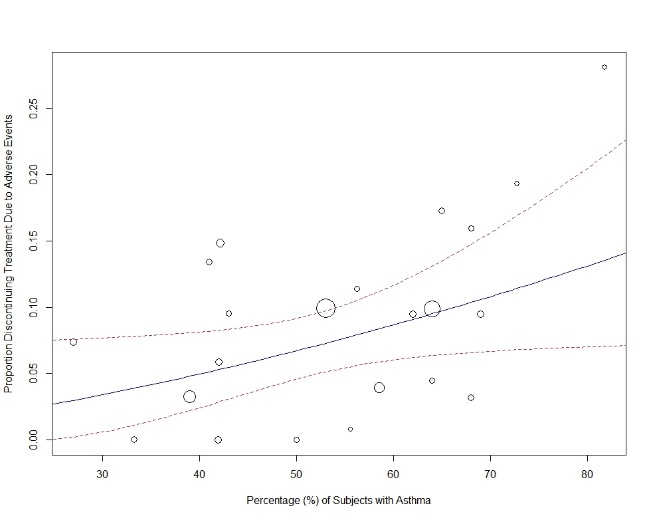

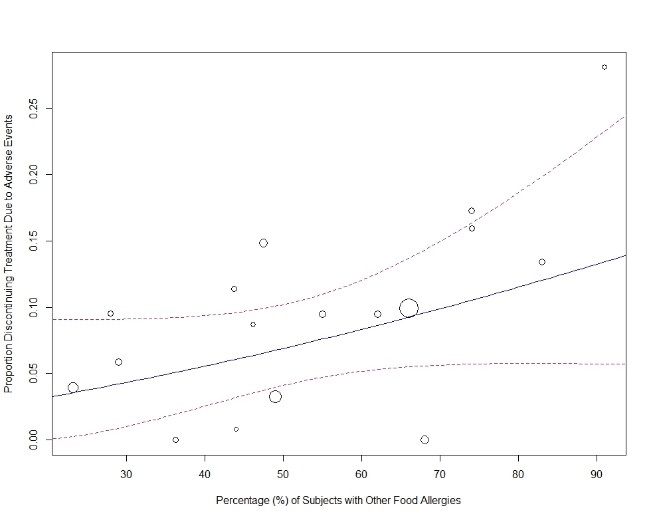

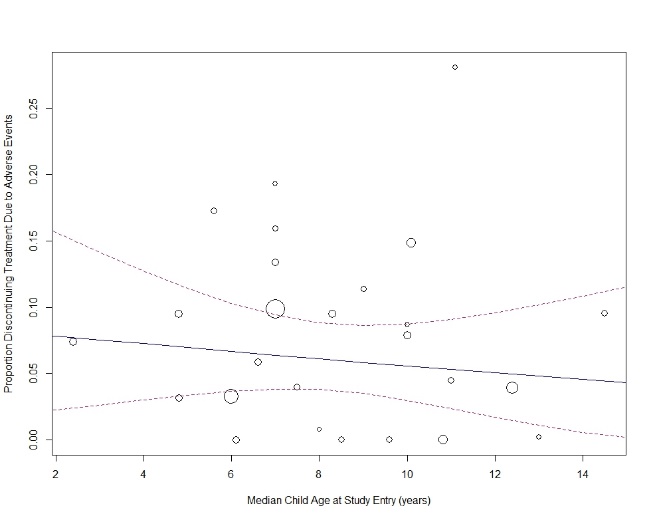

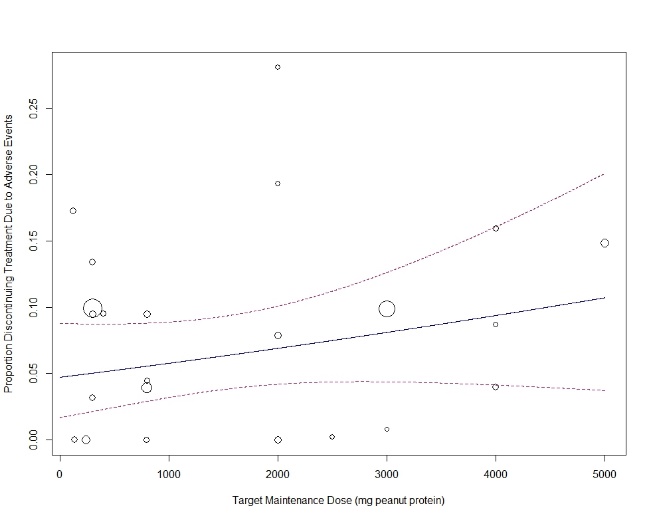

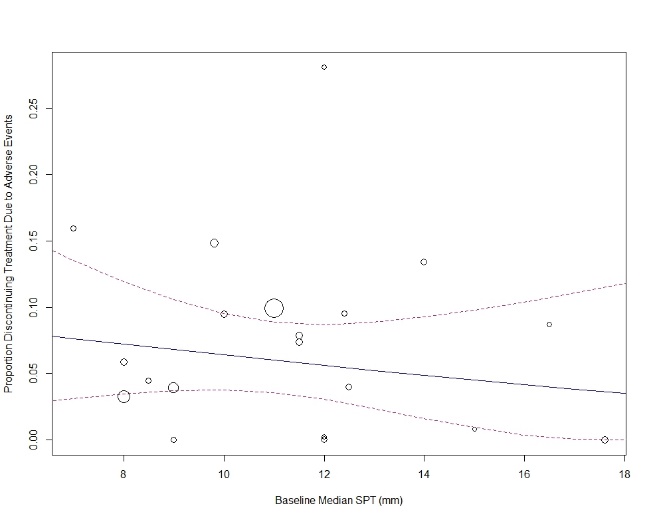

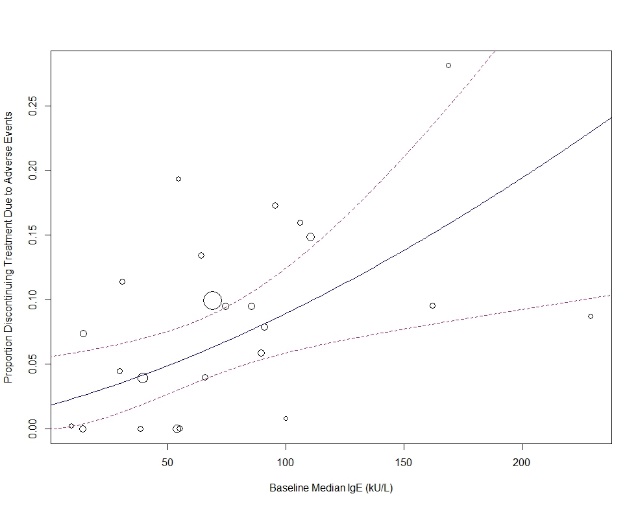
S4 Figure. Meta-regression bubble plots - Adverse Event Causing Treatment Discontinuation

**p=0.0059**

p=0.4557

p=0.5544

p=0.2245

**p=0.0402**

p=0.2044

p=0.7889

p=0.0989

S5 Figure. Forest plots - Risk of Adverse Events Requiring Treatment


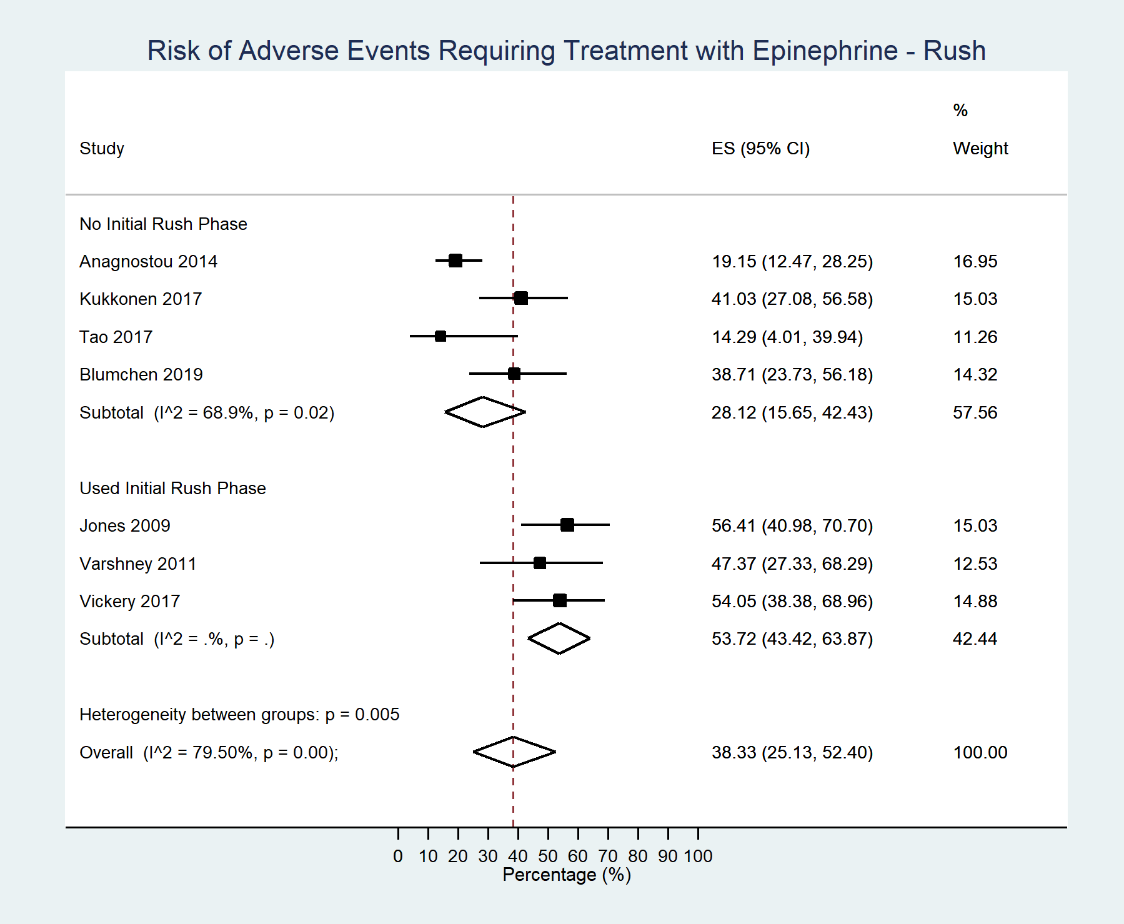
(A) Rush Phase


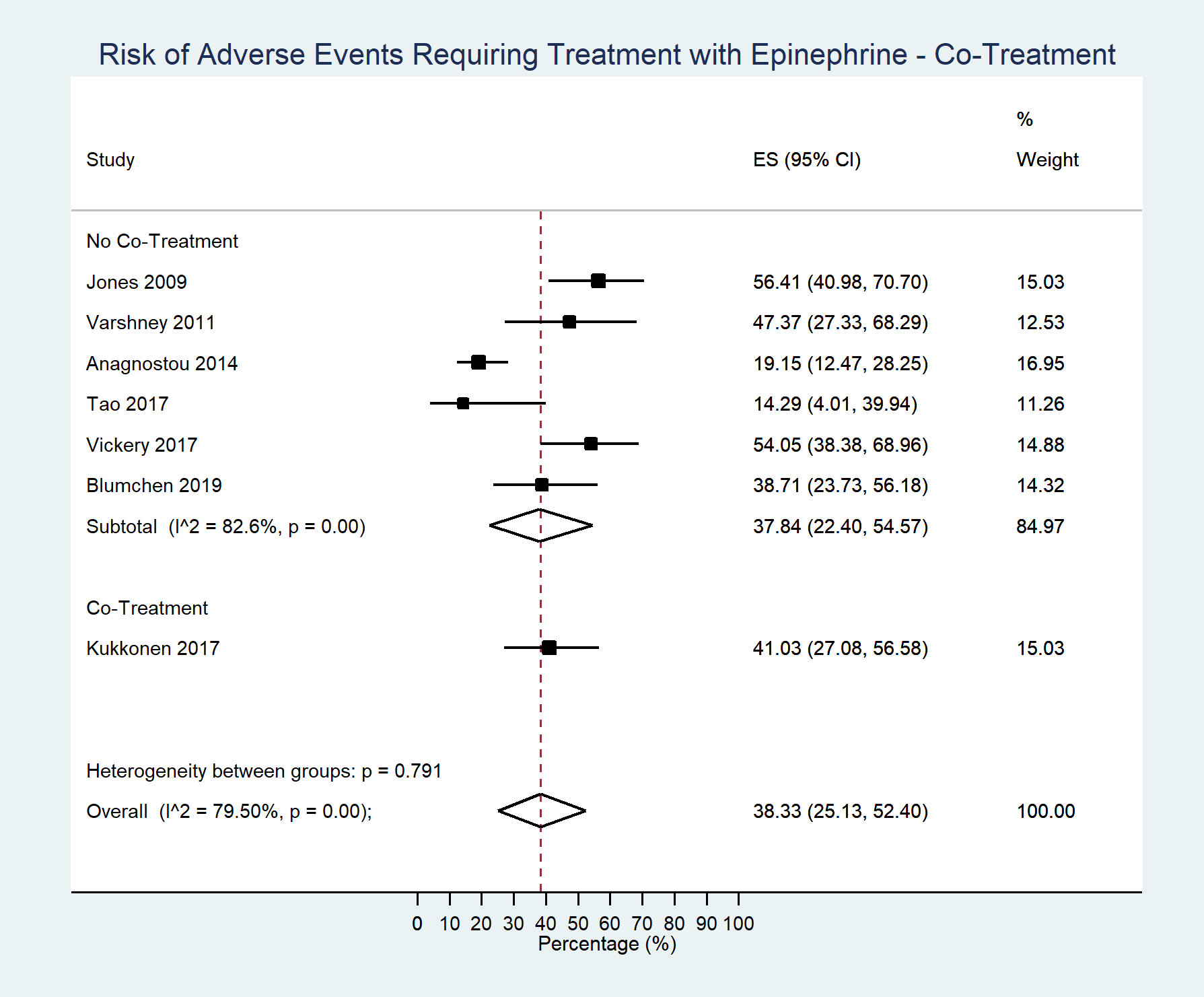
(B) Co-Treatment


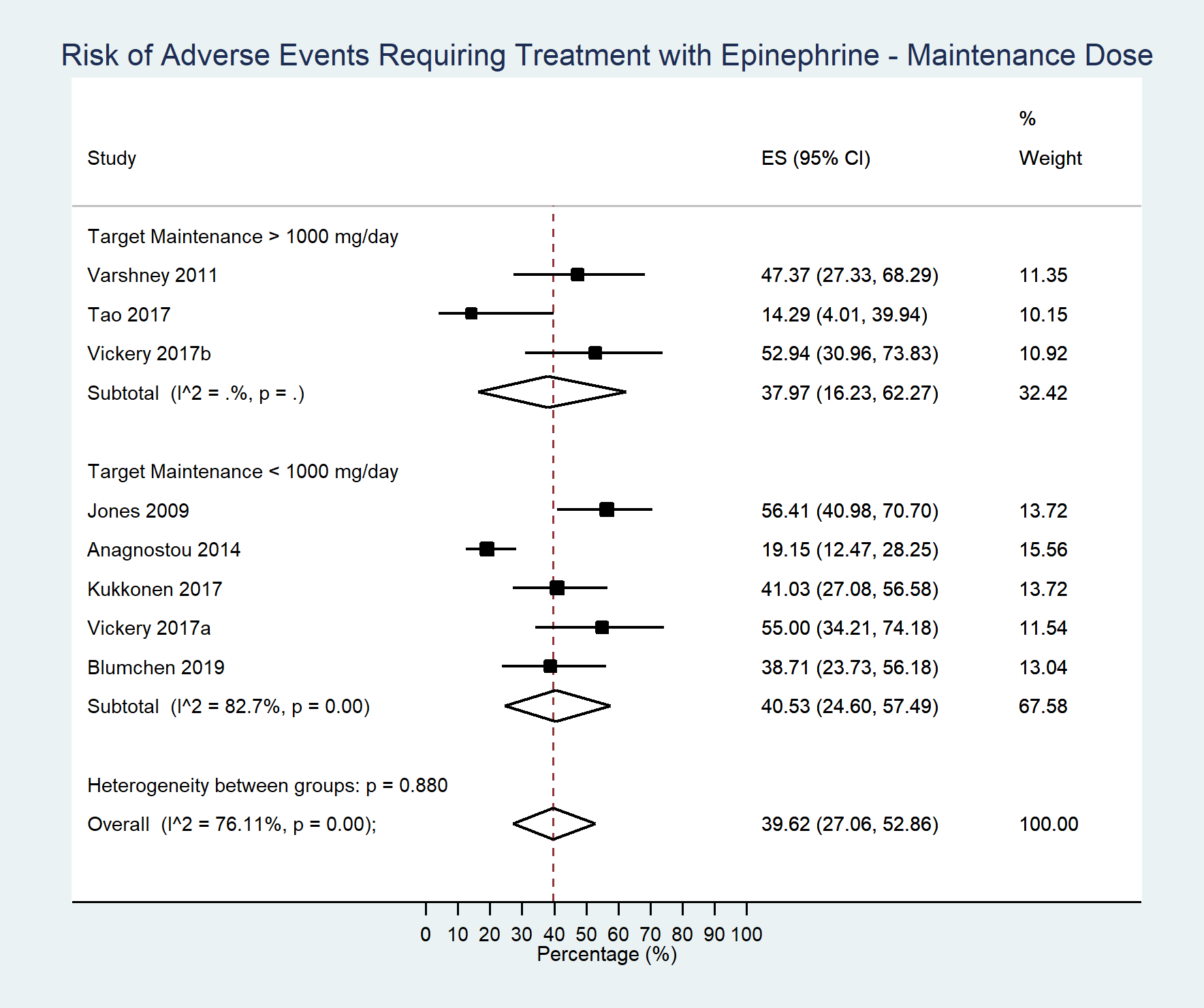
(C) Target Maintenance Dose


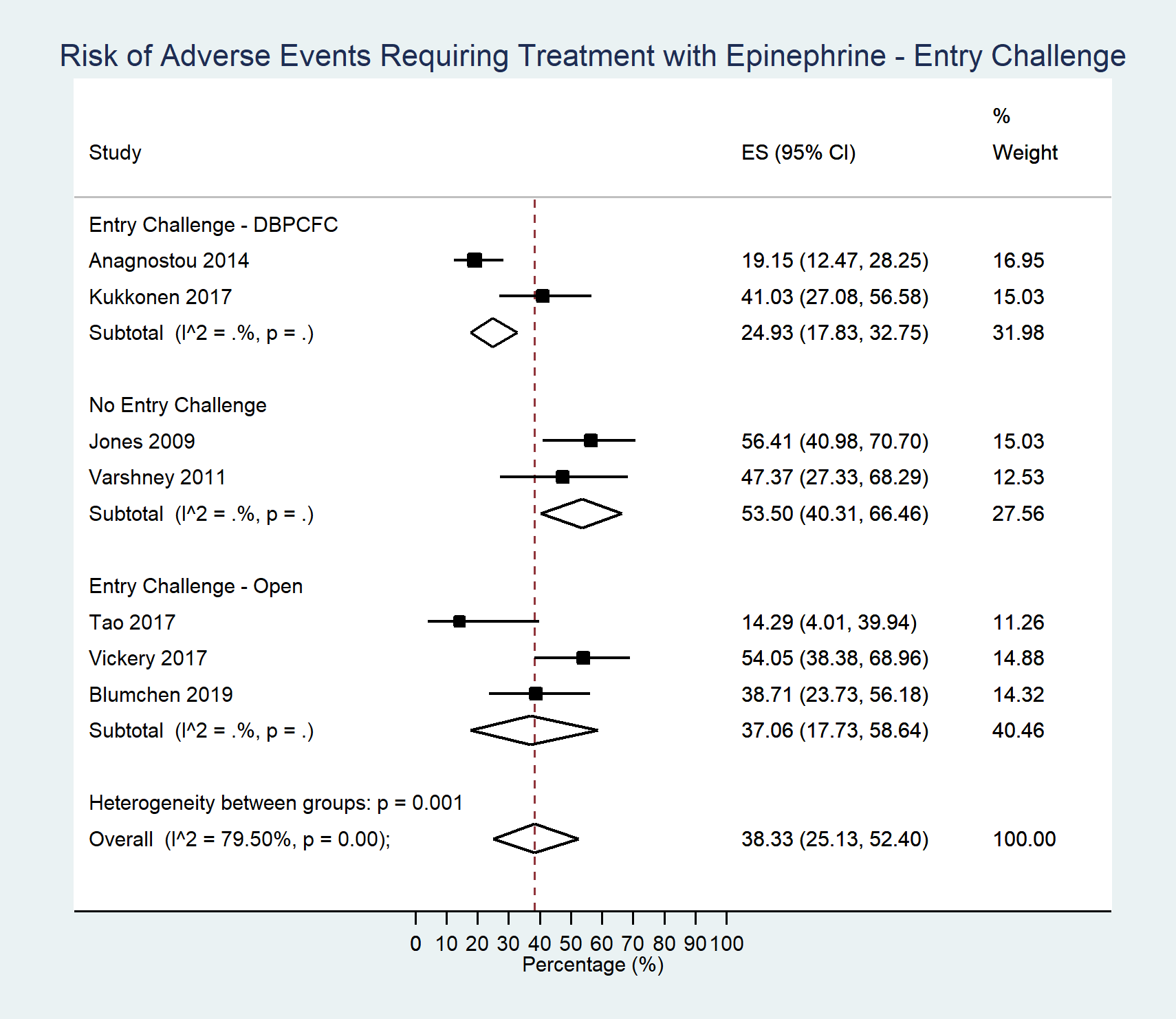
(D) Entry Oral Food Challenge


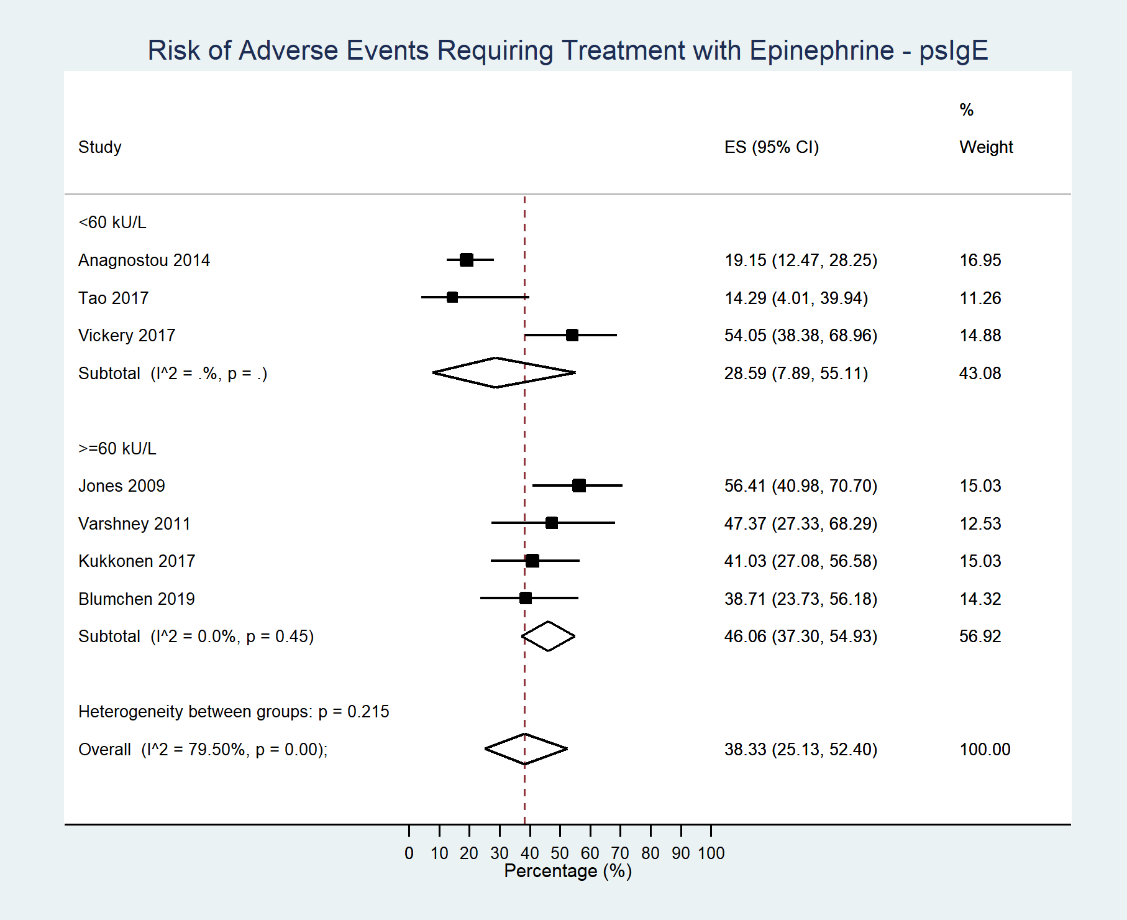
(E) Baseline Peanut Specific IgE (median)


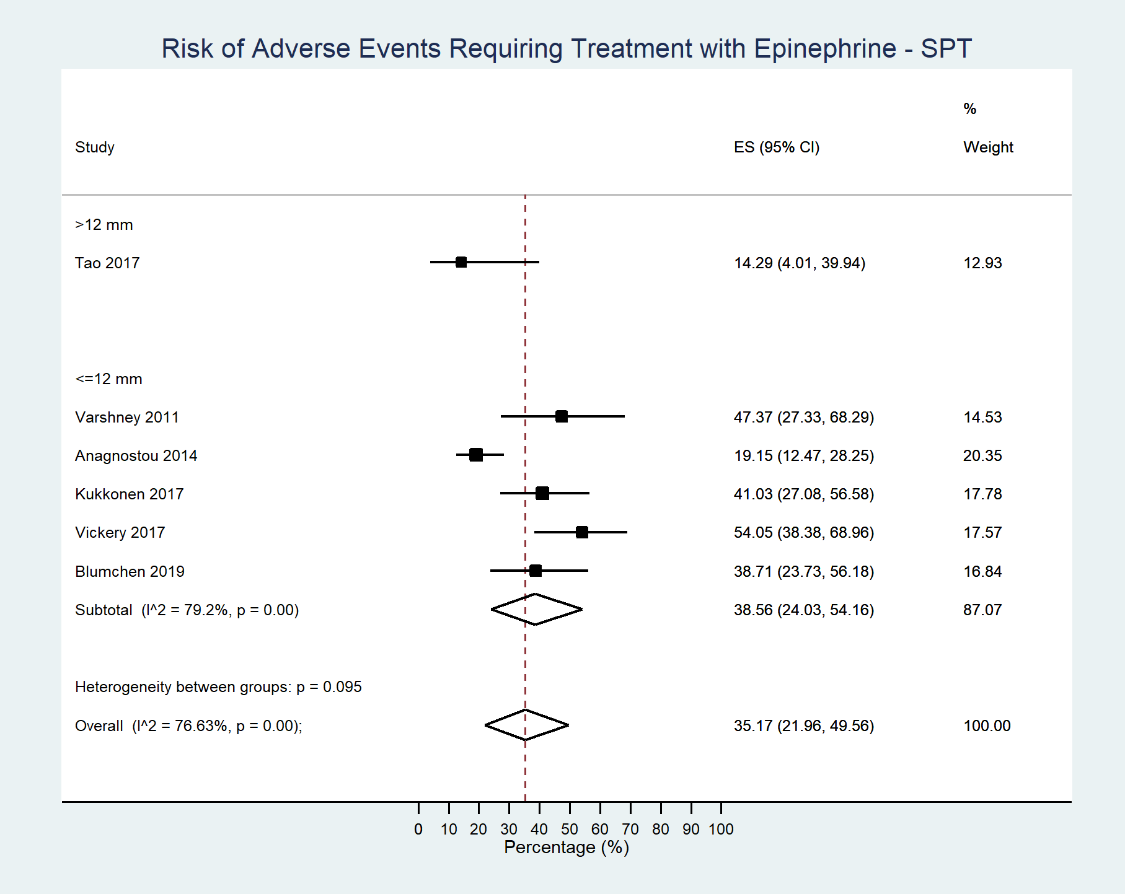
(F) Baseline SPT (median)

S6 Figure. Forest plots - Frequency of Adverse Events Requiring Treatment

(A) Rush Phase
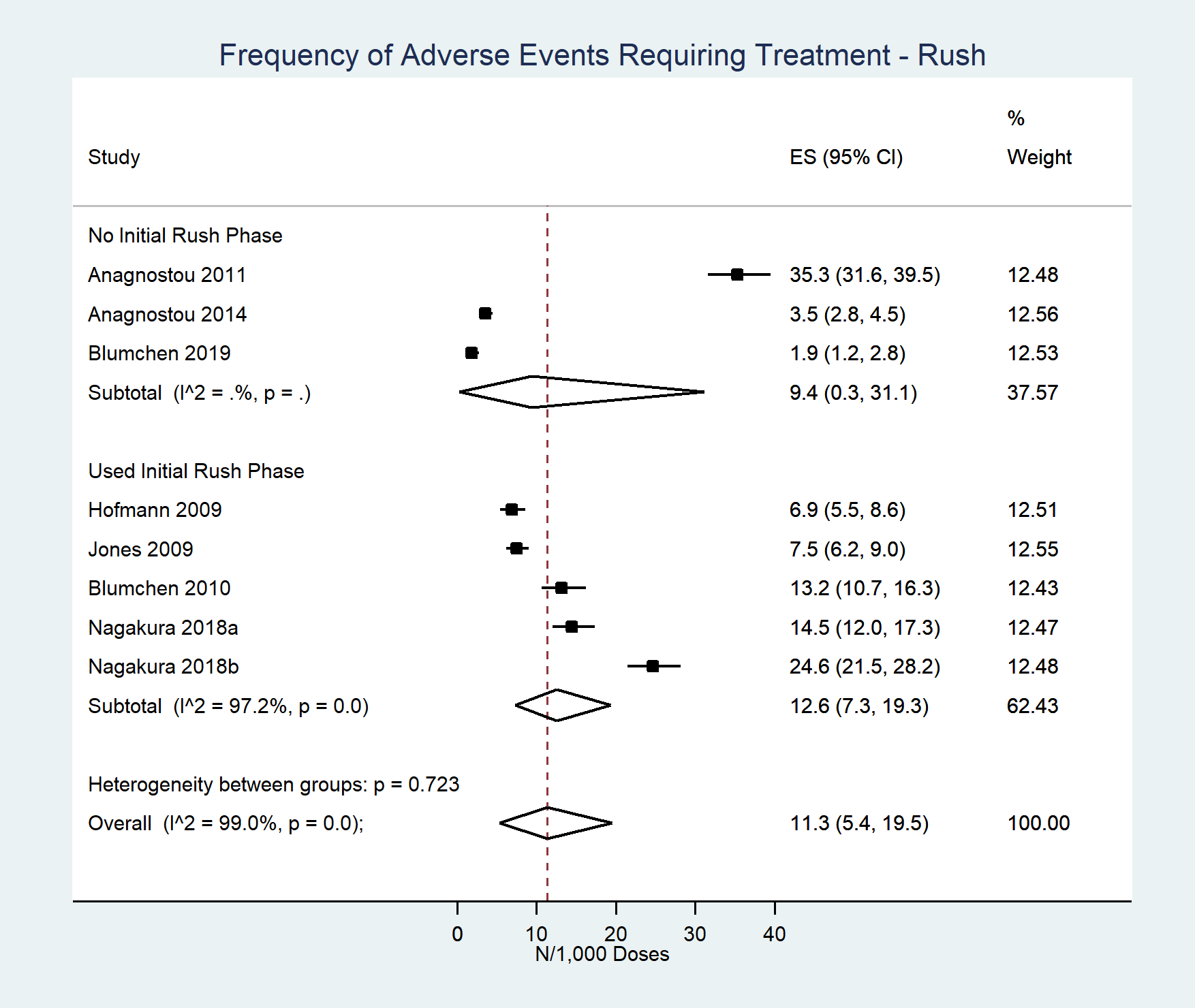


(B) Co-Treatment


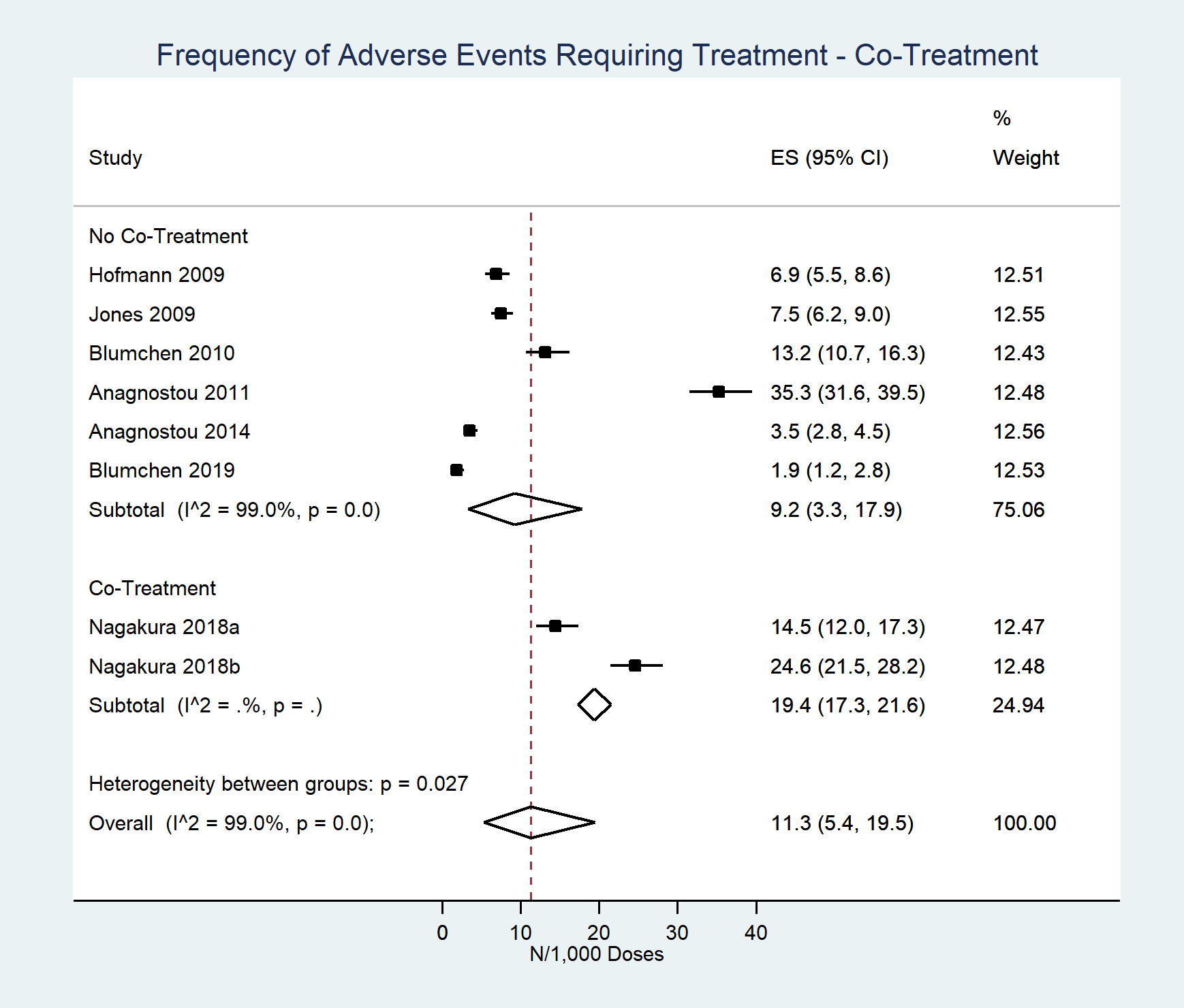


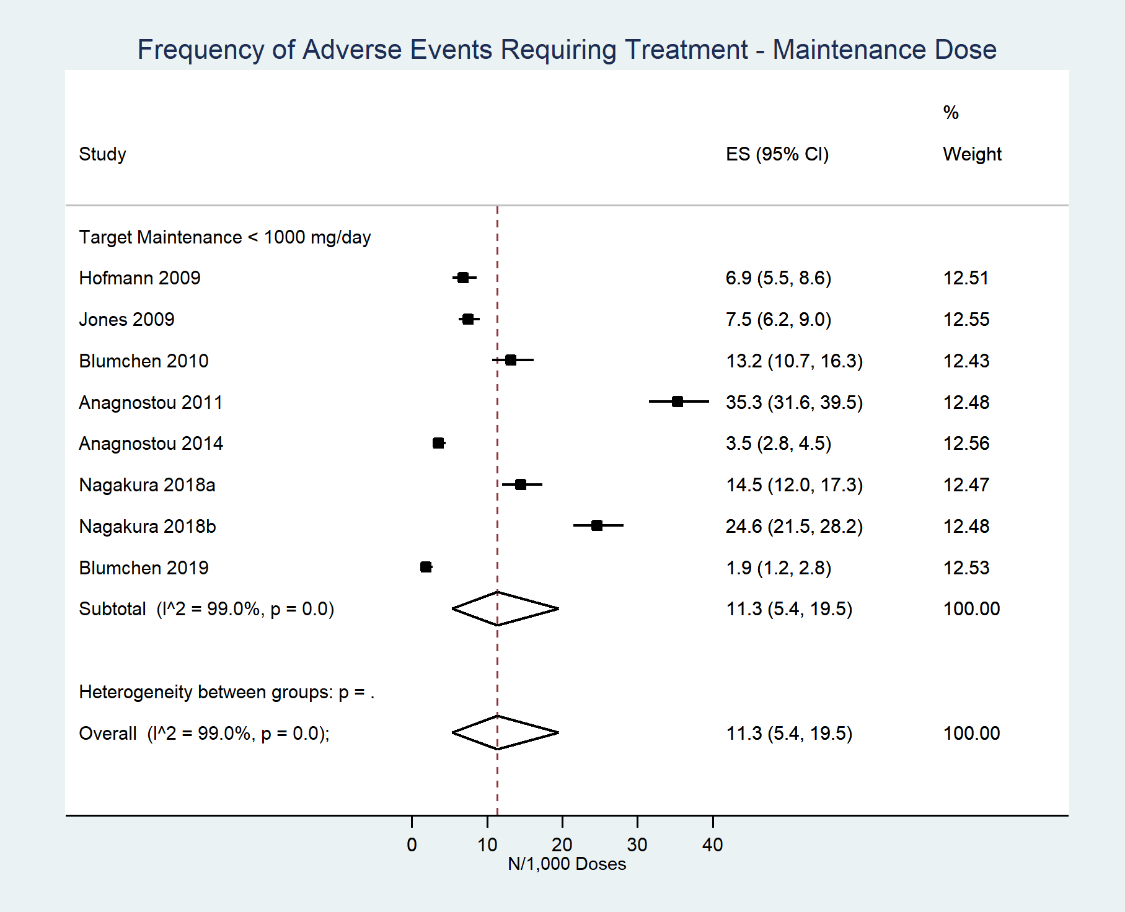
(C) Target Maintenance Dose

(D) Entry Oral Food Challenge


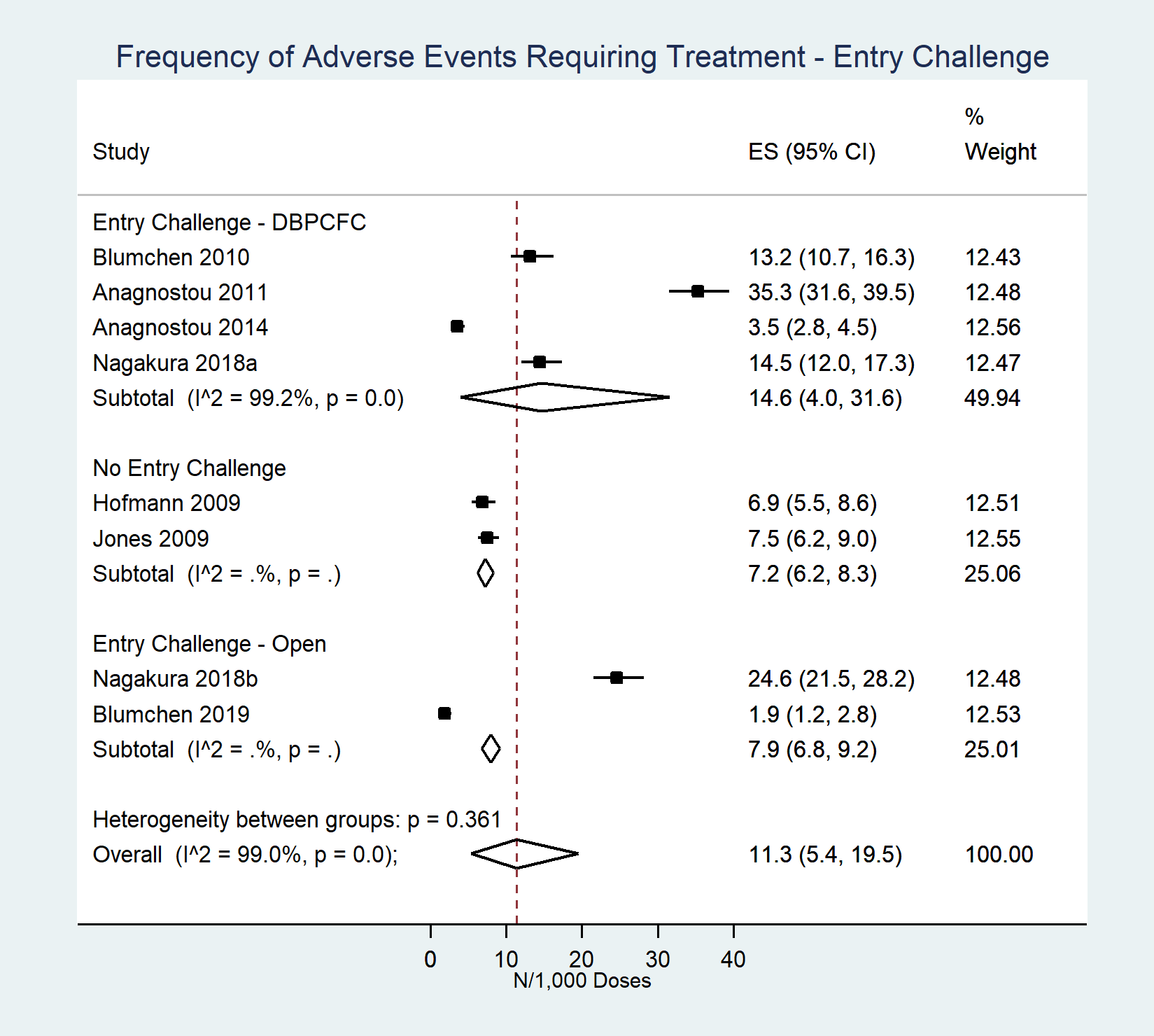


(E) Baseline Peanut Specific IgE (median)


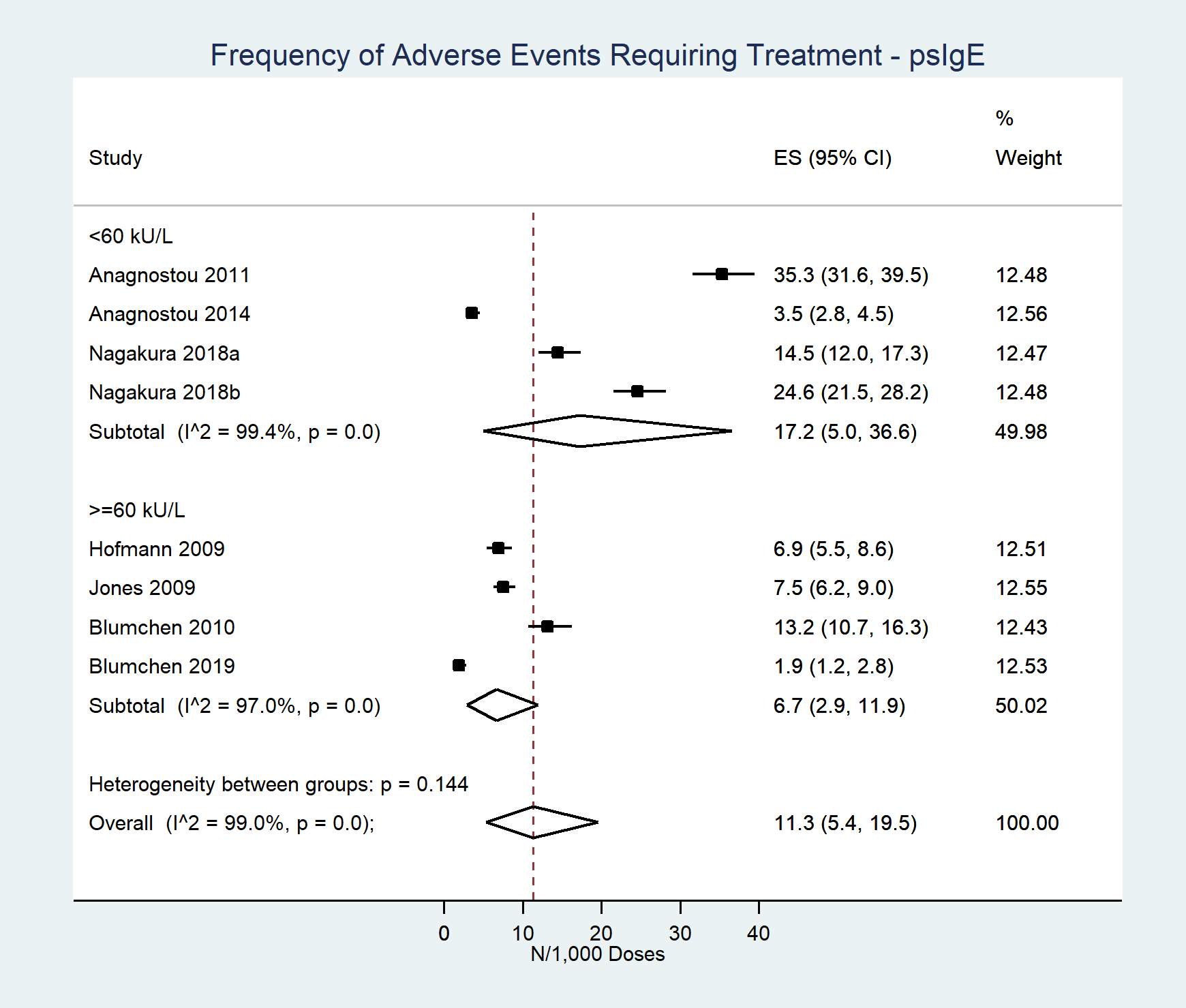


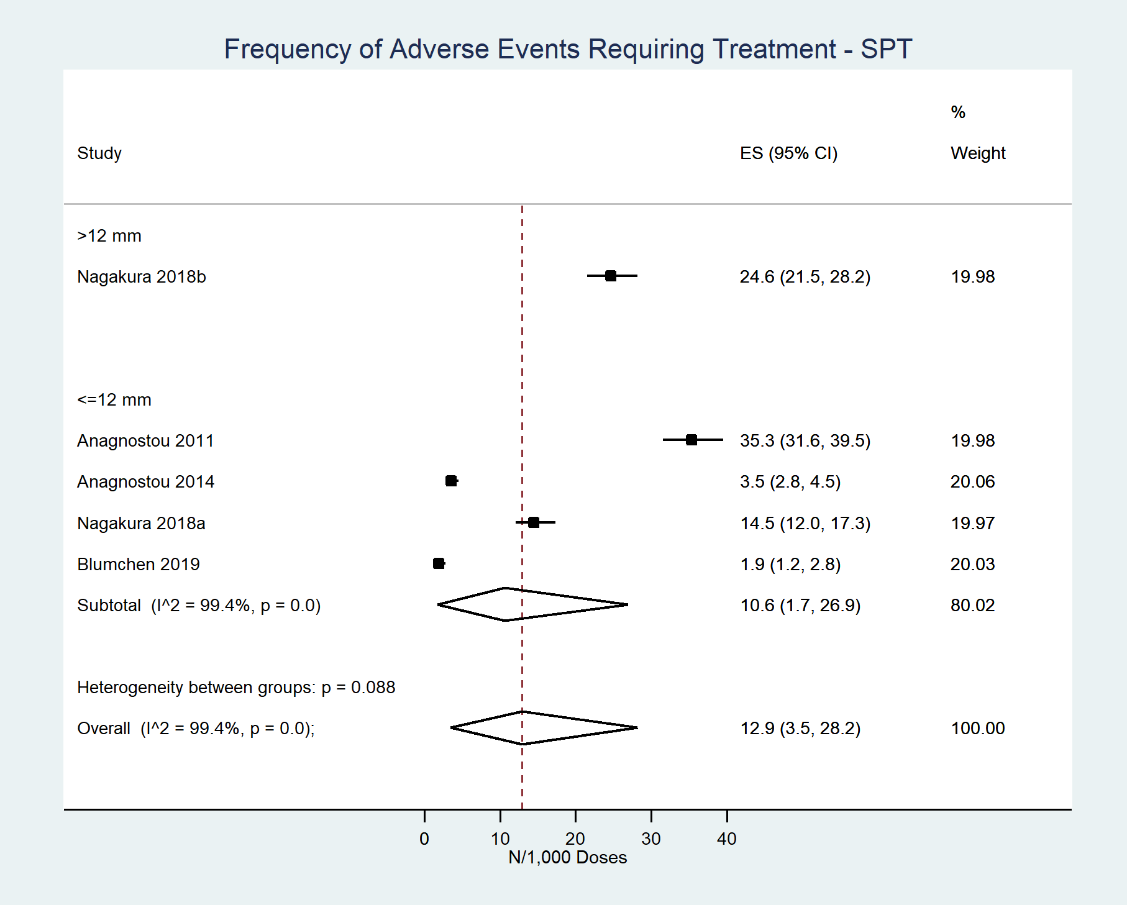
(F) Baseline SPT (median)

S7 Figure. Meta-regression bubble plots - risk of adverse events requiring medication treatment


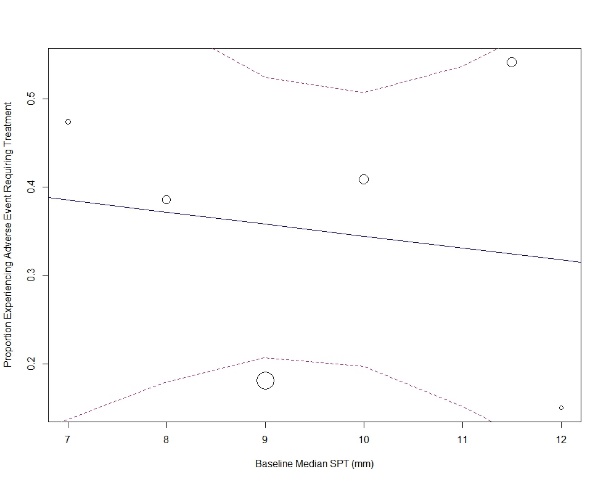

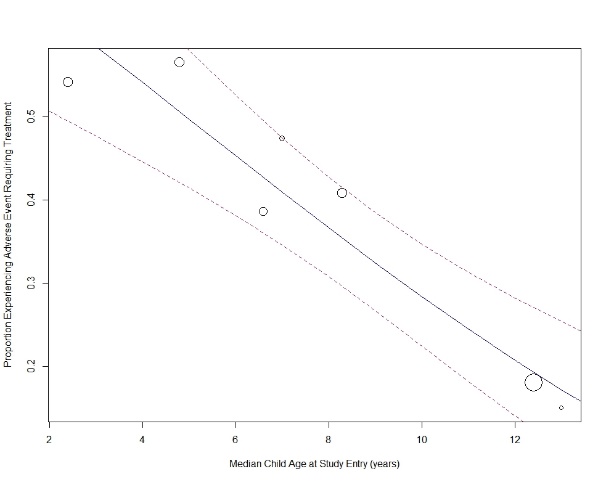

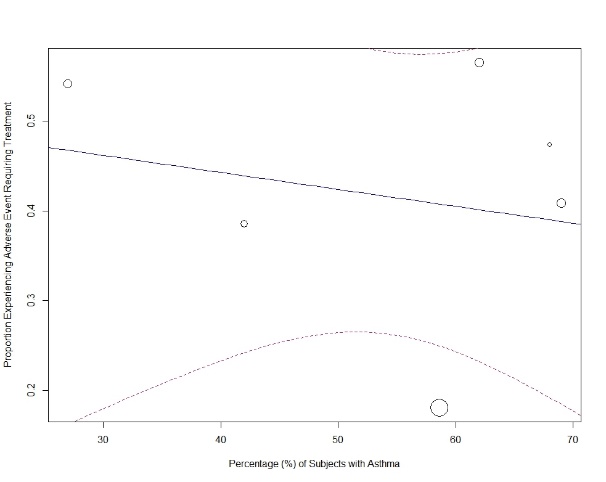

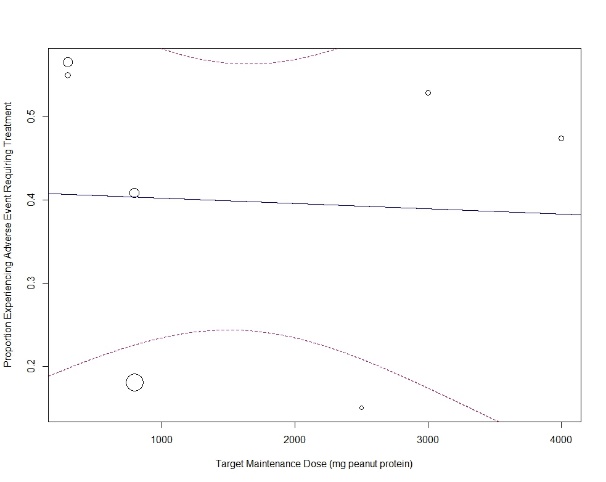

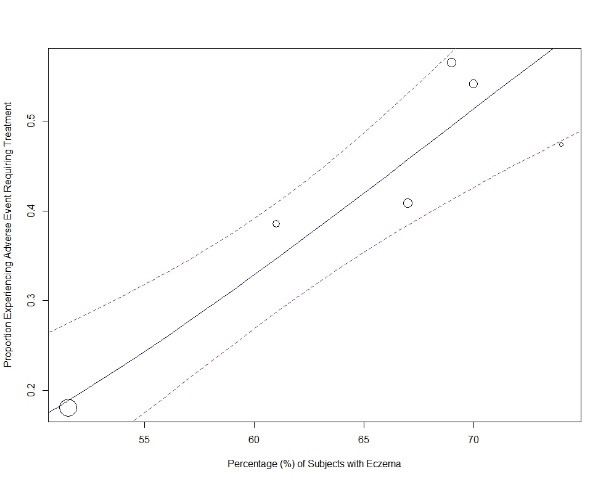

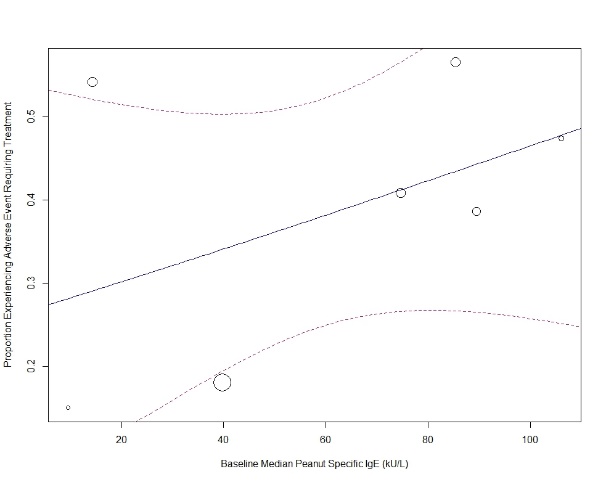

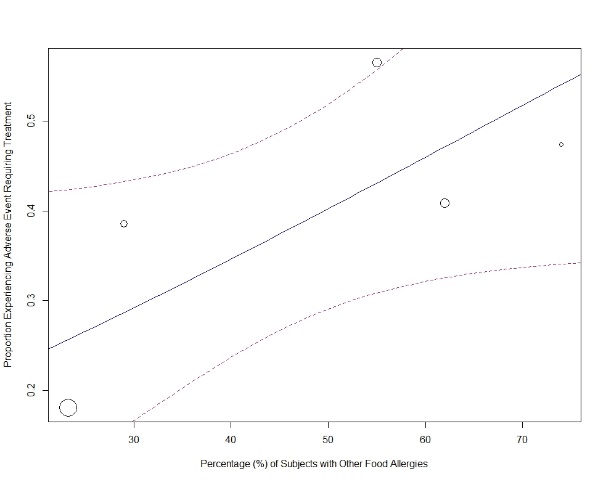

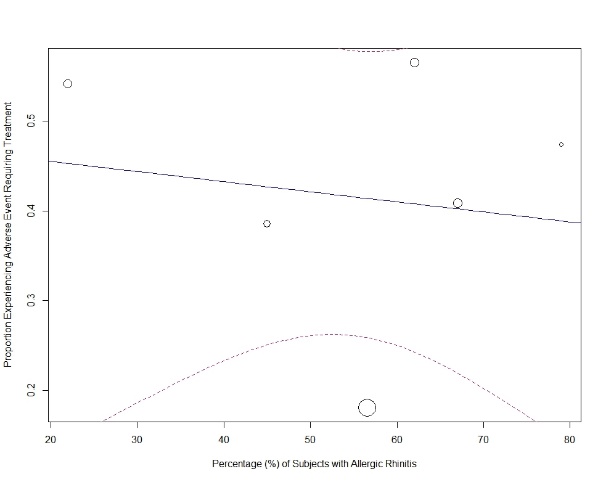


p=0.3172

p=0.7650

p=0.9172

**P<0.001**

p=0.0562

p=0.7275

p=0.8085

**P<0.001**

S8 Figure. Meta-regression bubble plots - Frequency of Adverse Events Requiring Medication Treatment

p=0.4021

p=0.2366


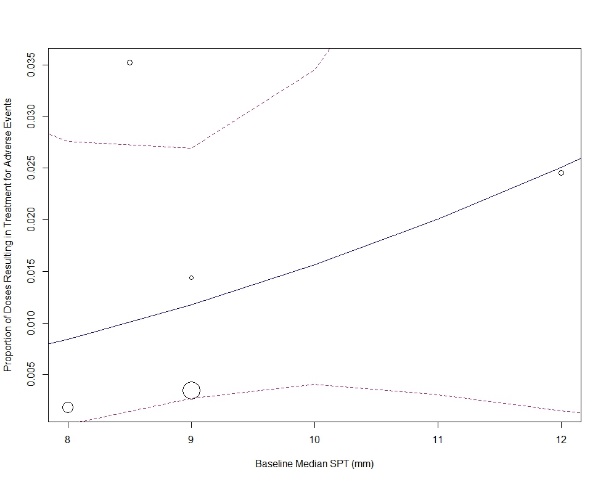

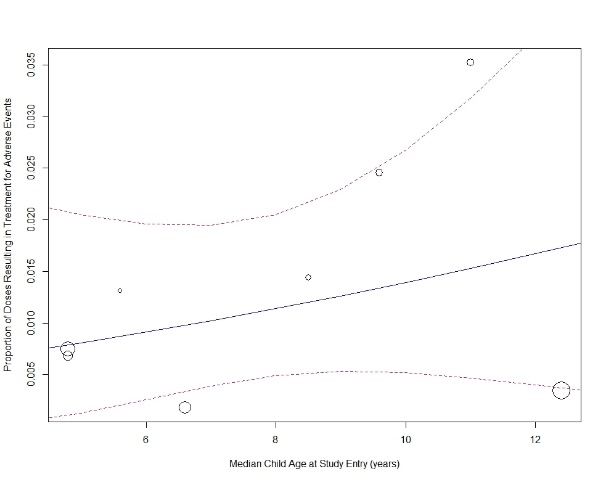

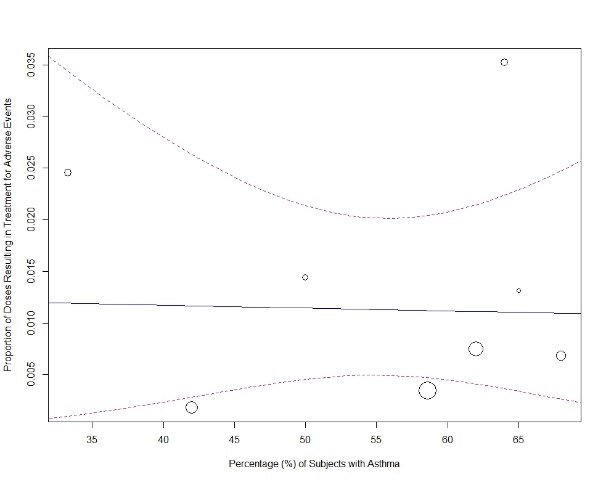

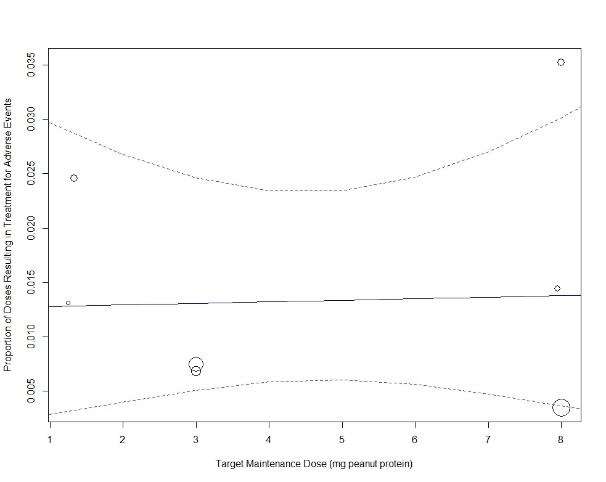

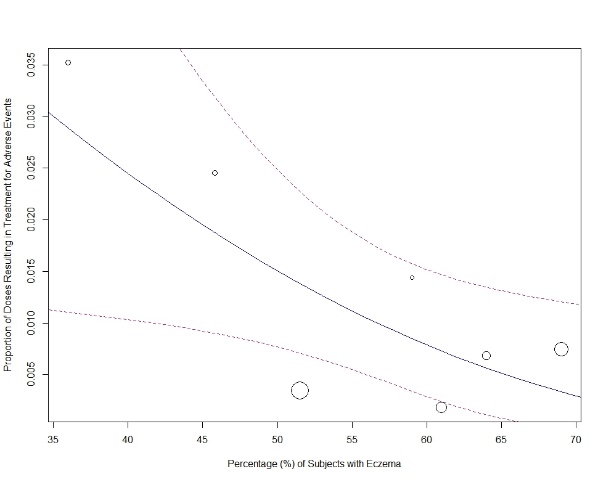

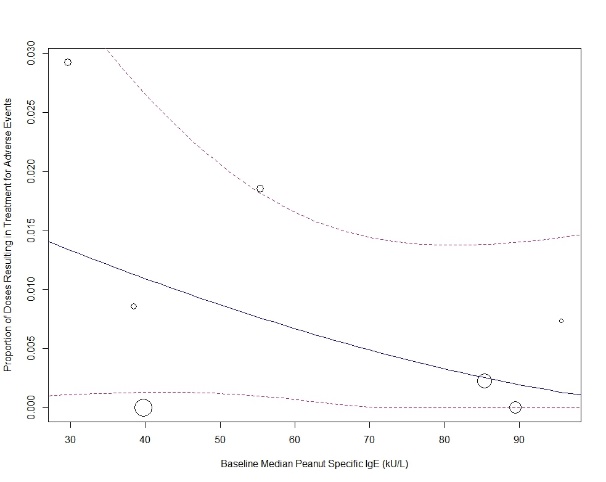

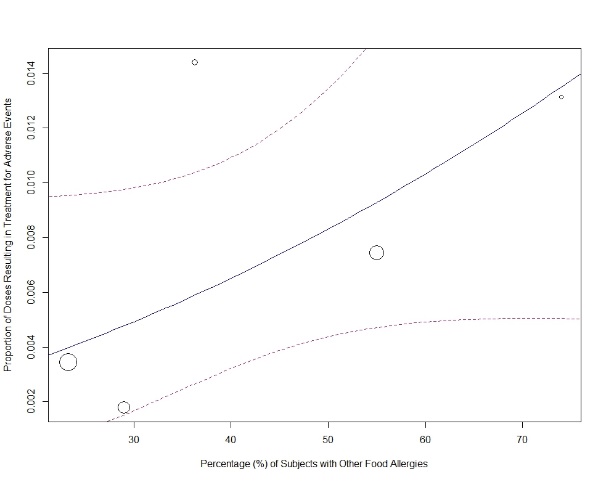

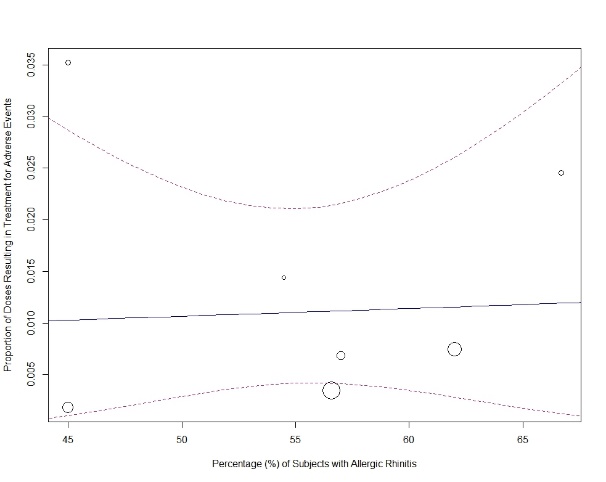


p=0.9250

p=0.4096

**p=0.0248**

p=0.9343

p=0.8960

p=0.1042

S9 Figure. Risk of Adverse Events According to Treatment Phase


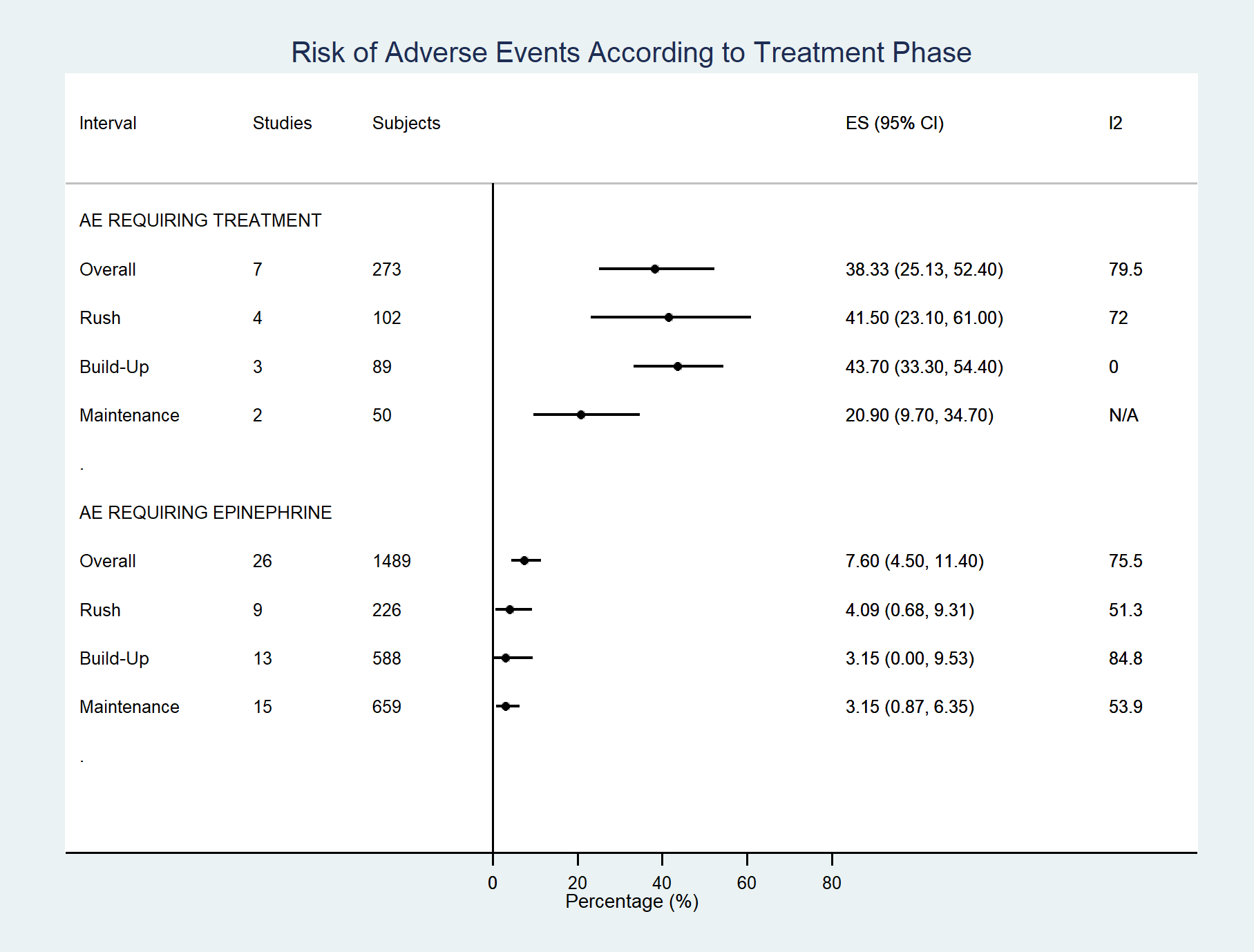


# **S10 Figure. Frequency of Adverse Events According to Treatment Phase**


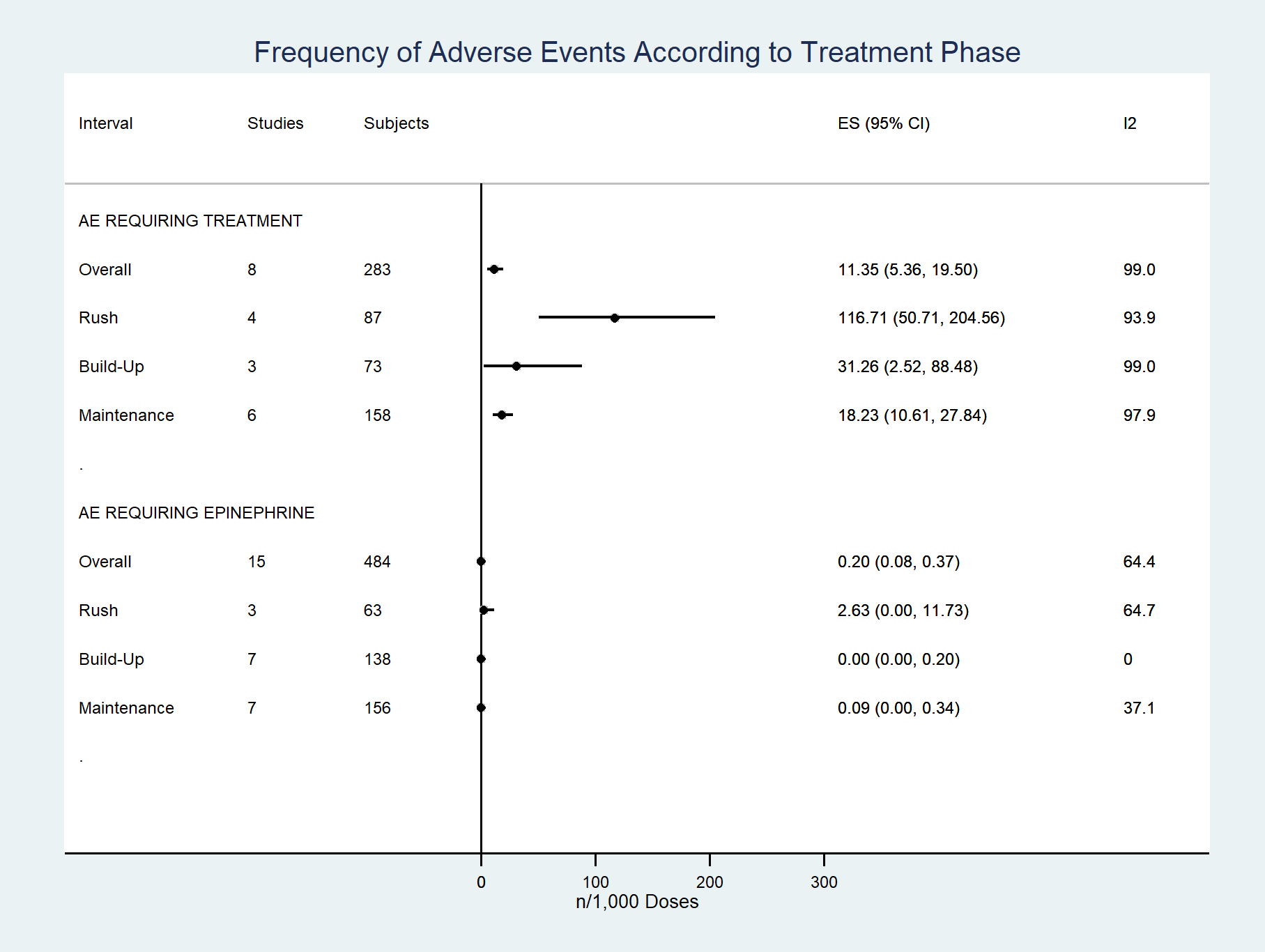


S11 Figure. Forest plots - Risk of Adverse Events Requiring Treatment with Epinephrine


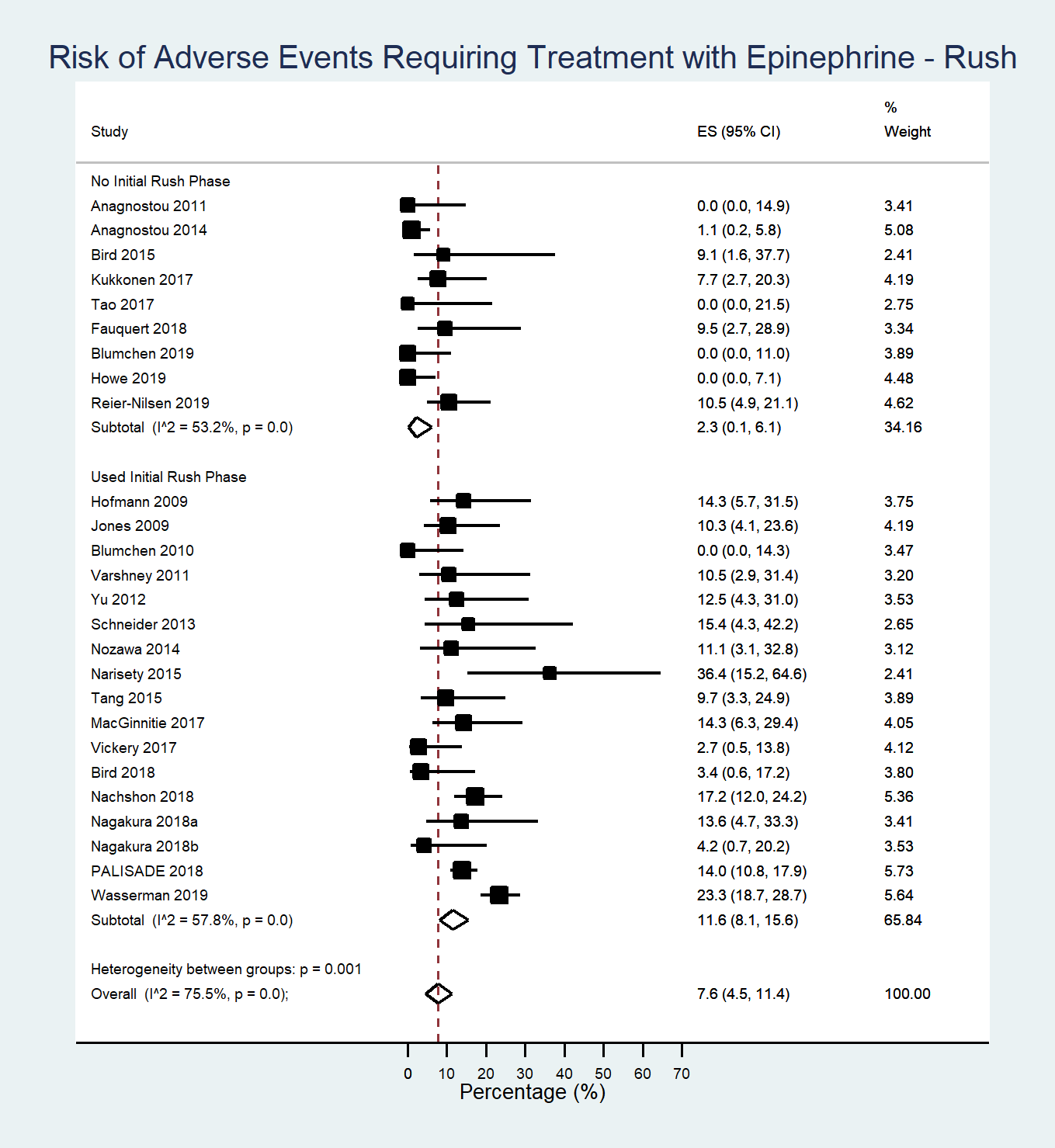
(A) Rush Phase


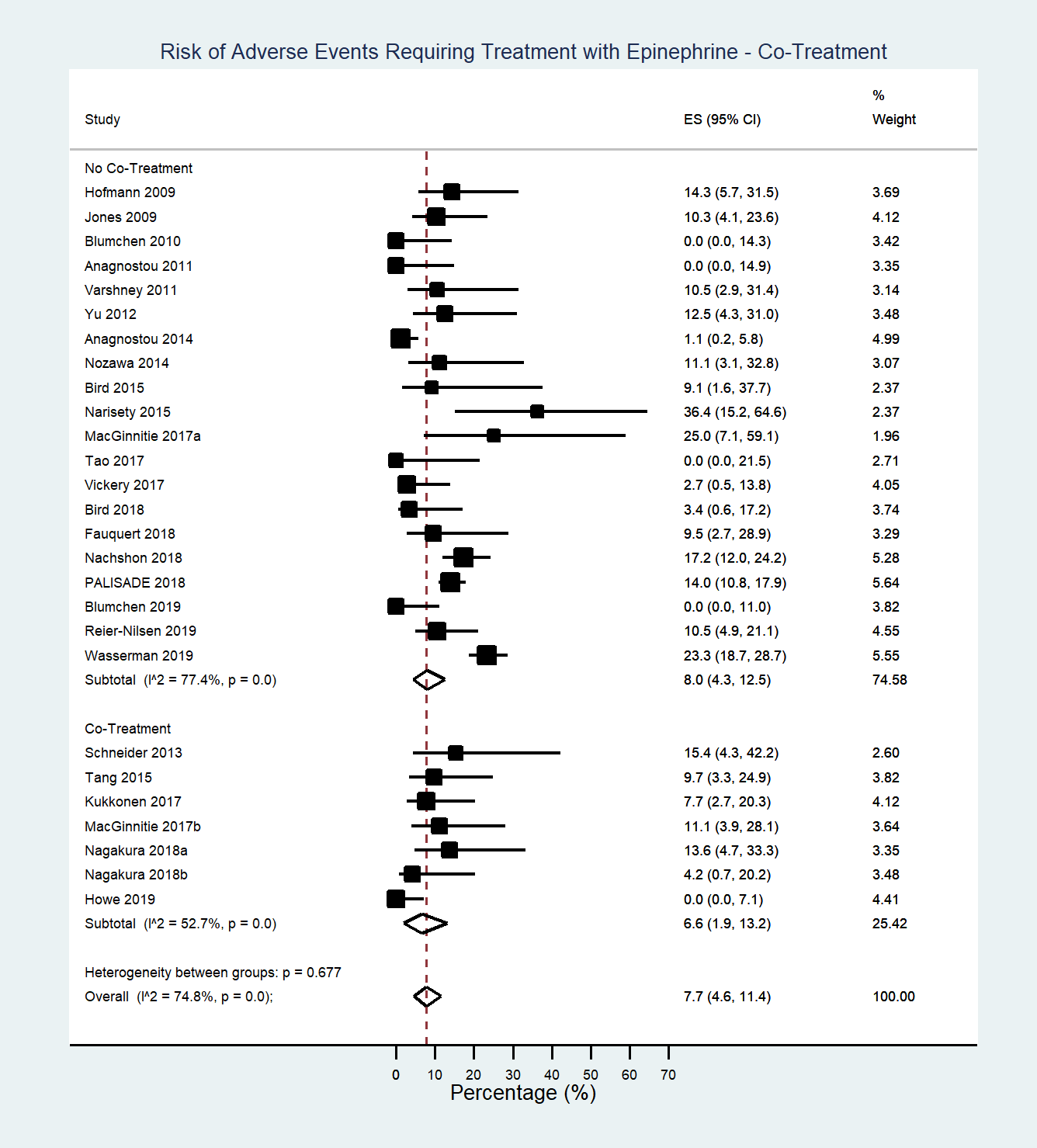
(B) Co-Treatment


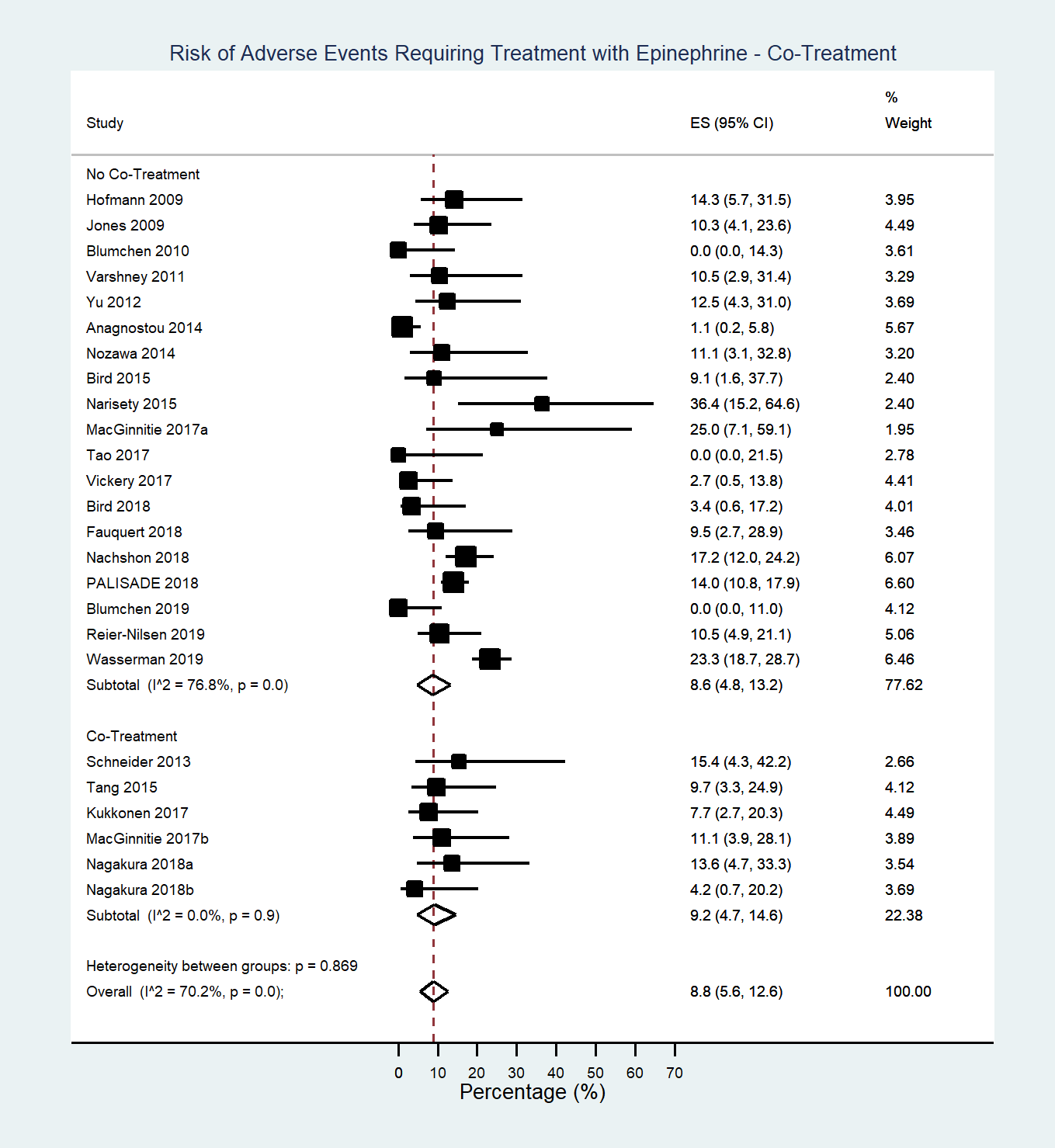


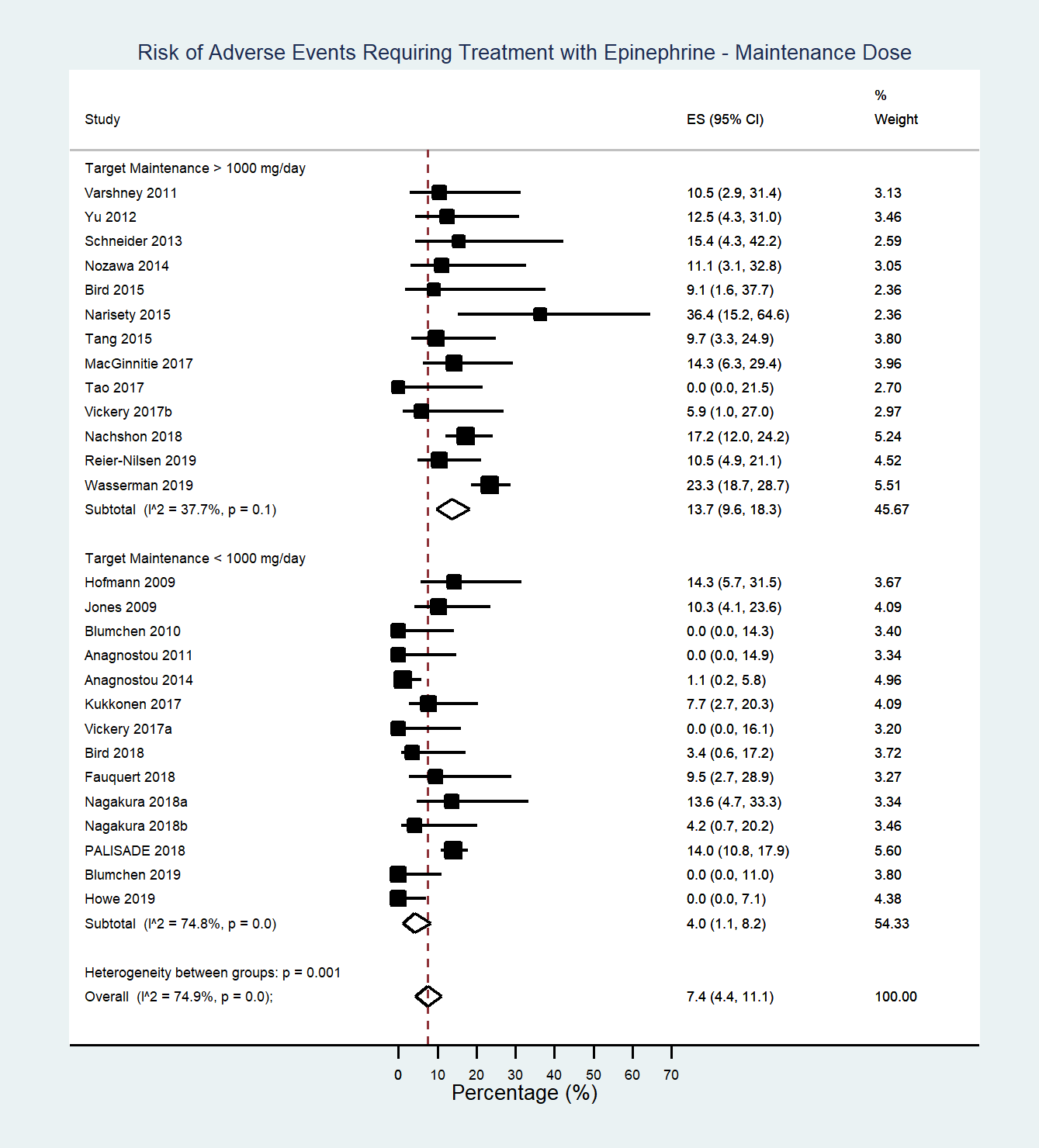
(C) Target Maintenance Dose


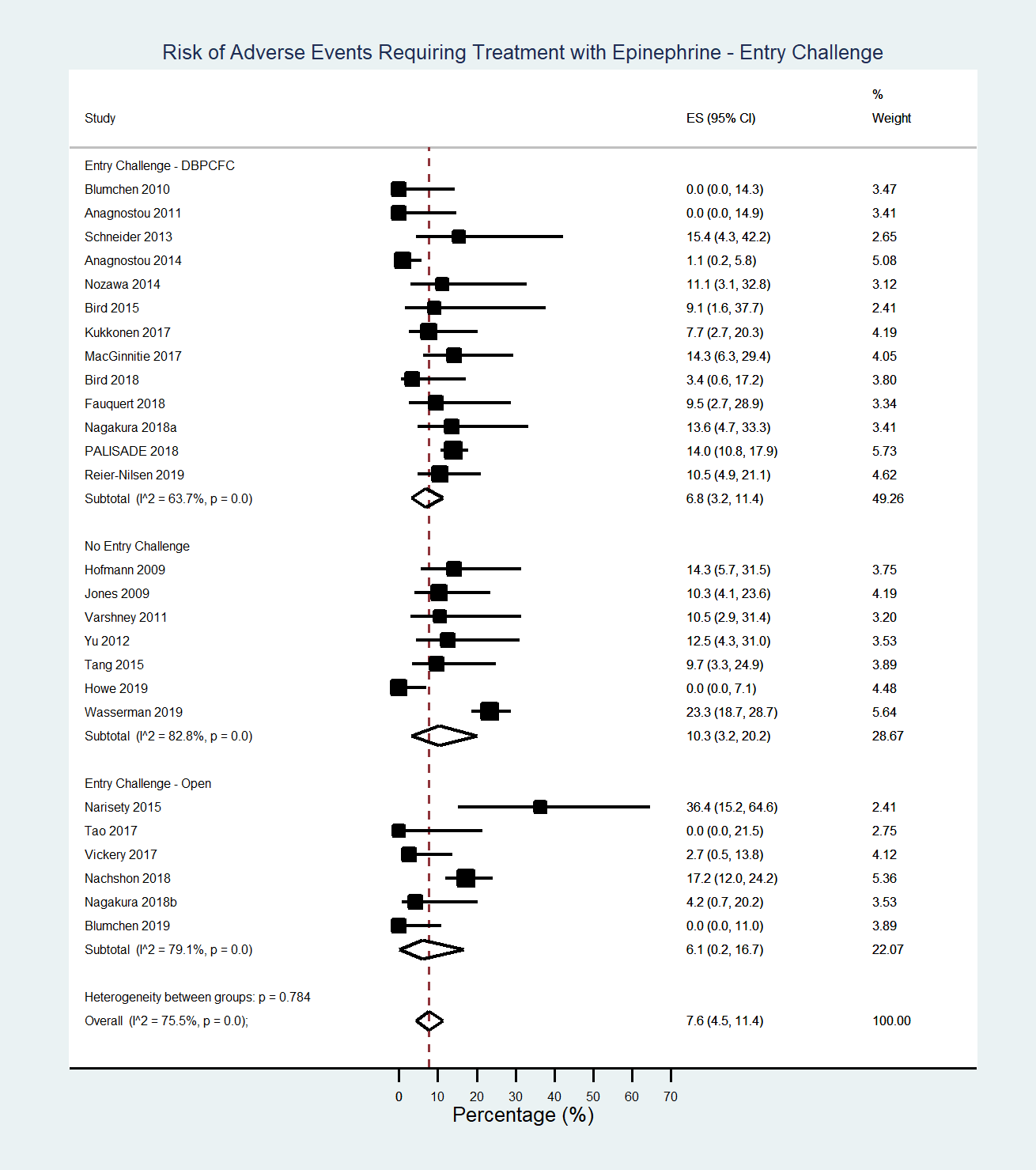
(D) Entry Oral Food Challenge


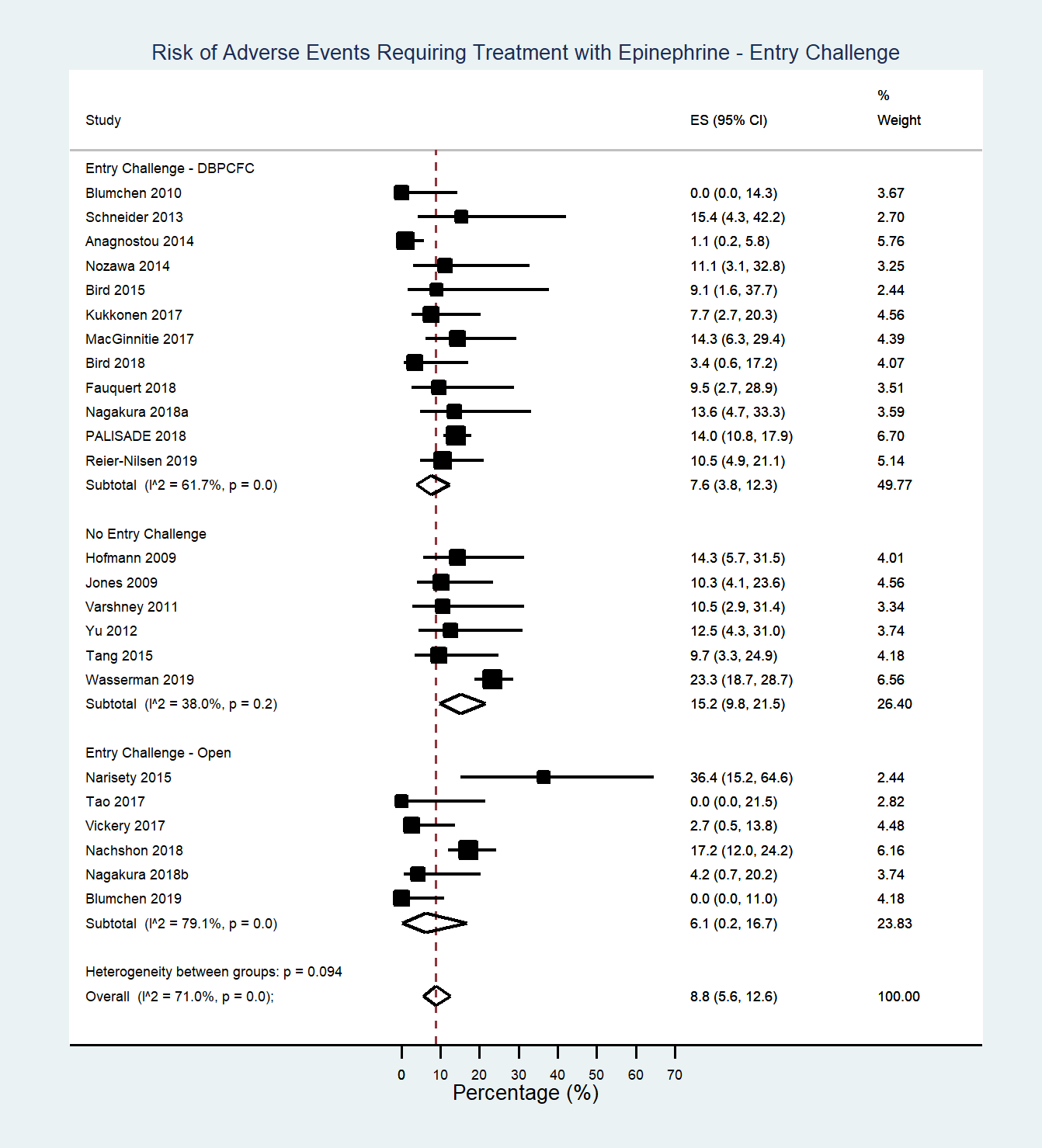


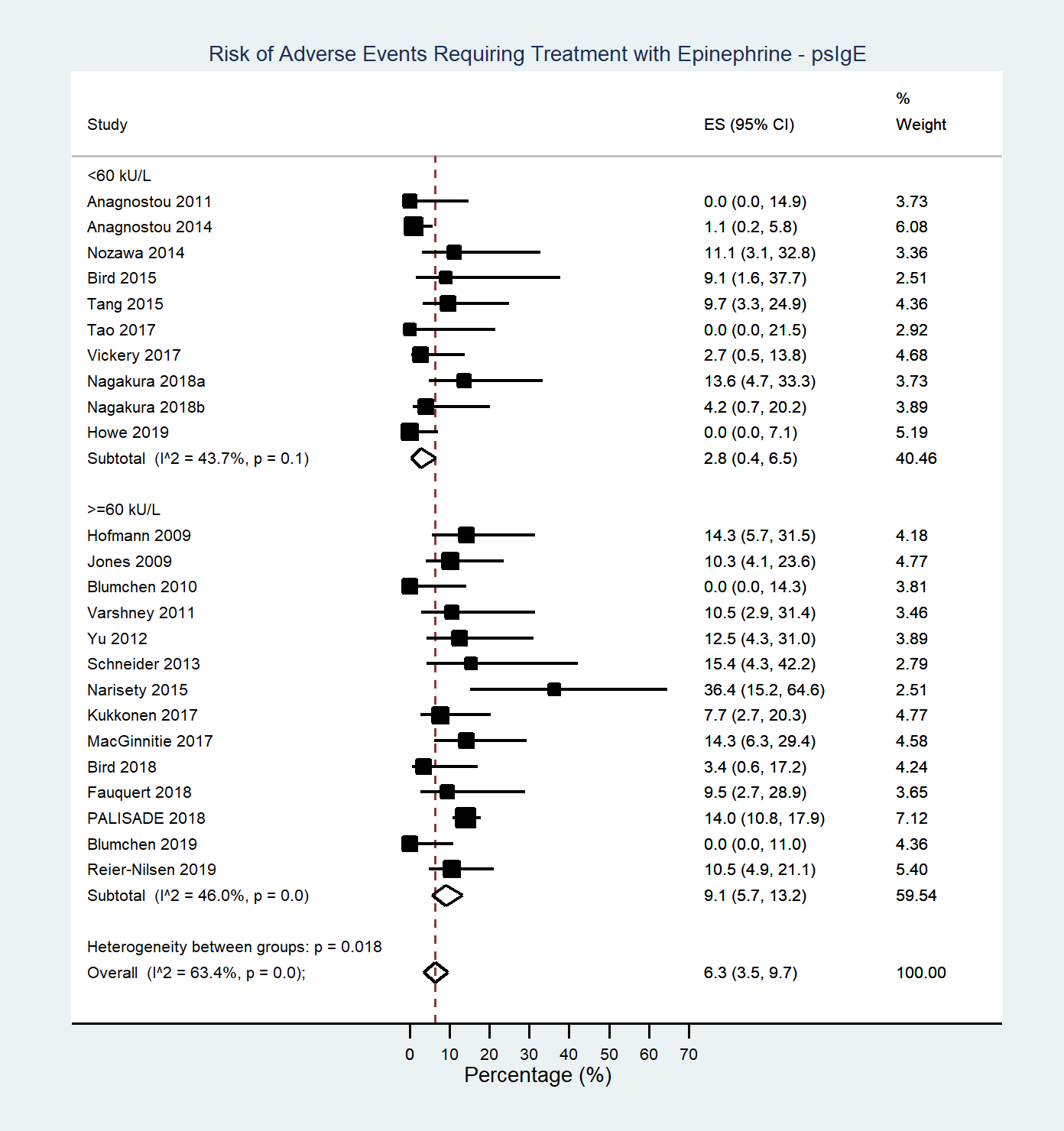
(F) Baseline Peanut Specific IgE (median)


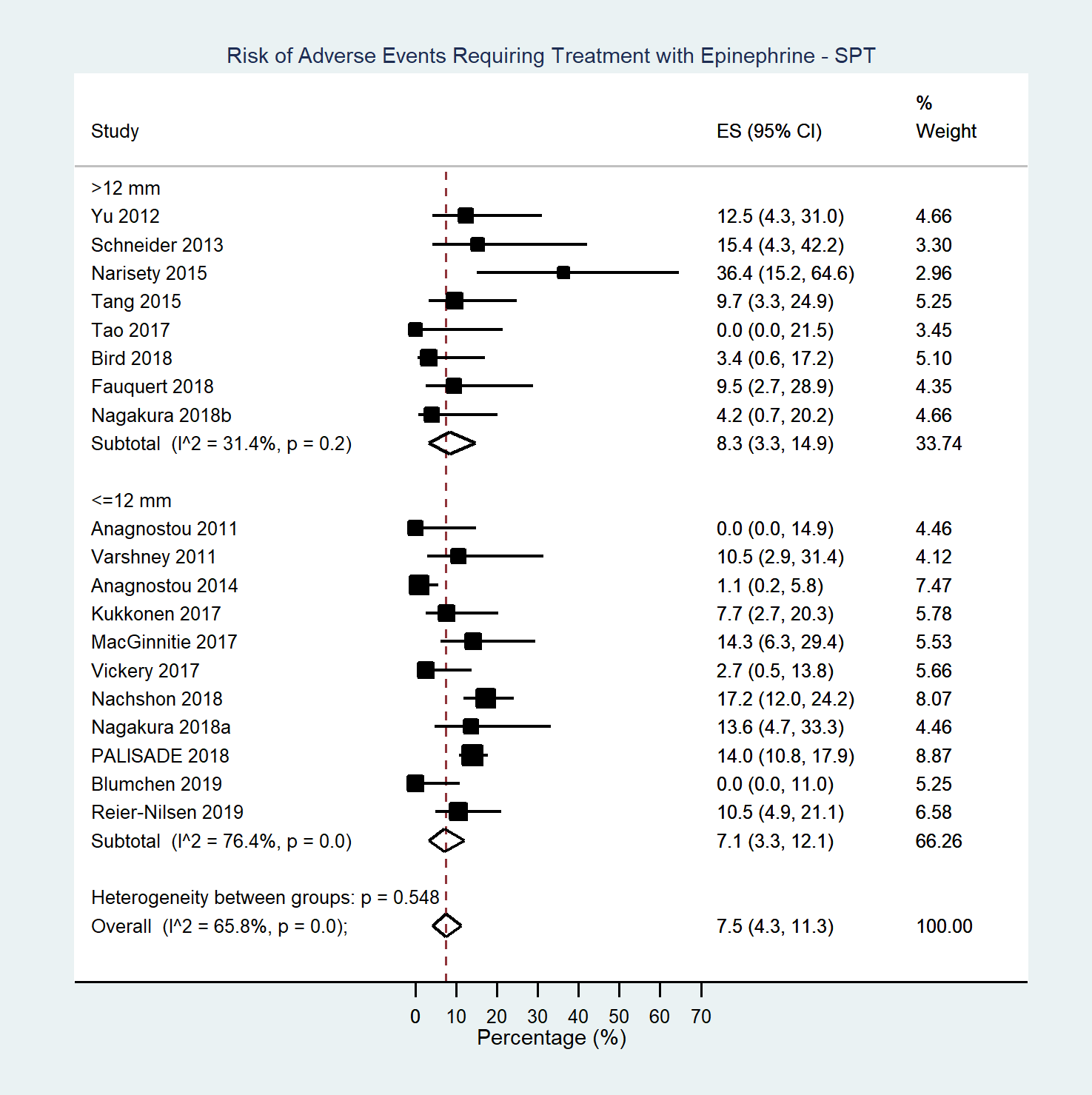
(G) Baseline SPT (median)

S12 Figure. Forest plots - Frequency of Adverse Events Requiring Treatment with Epinephrine

(A) Rush Phase


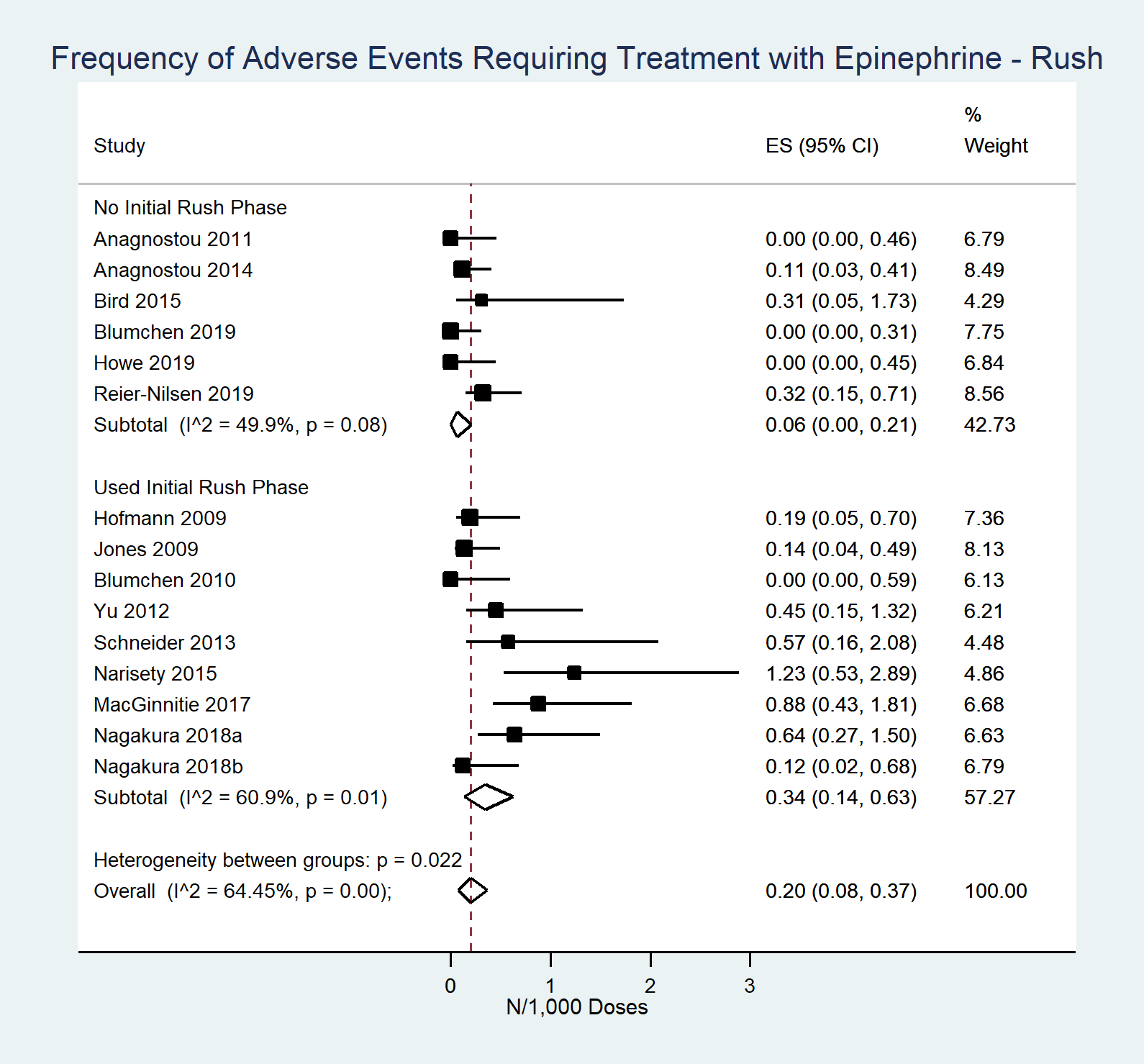

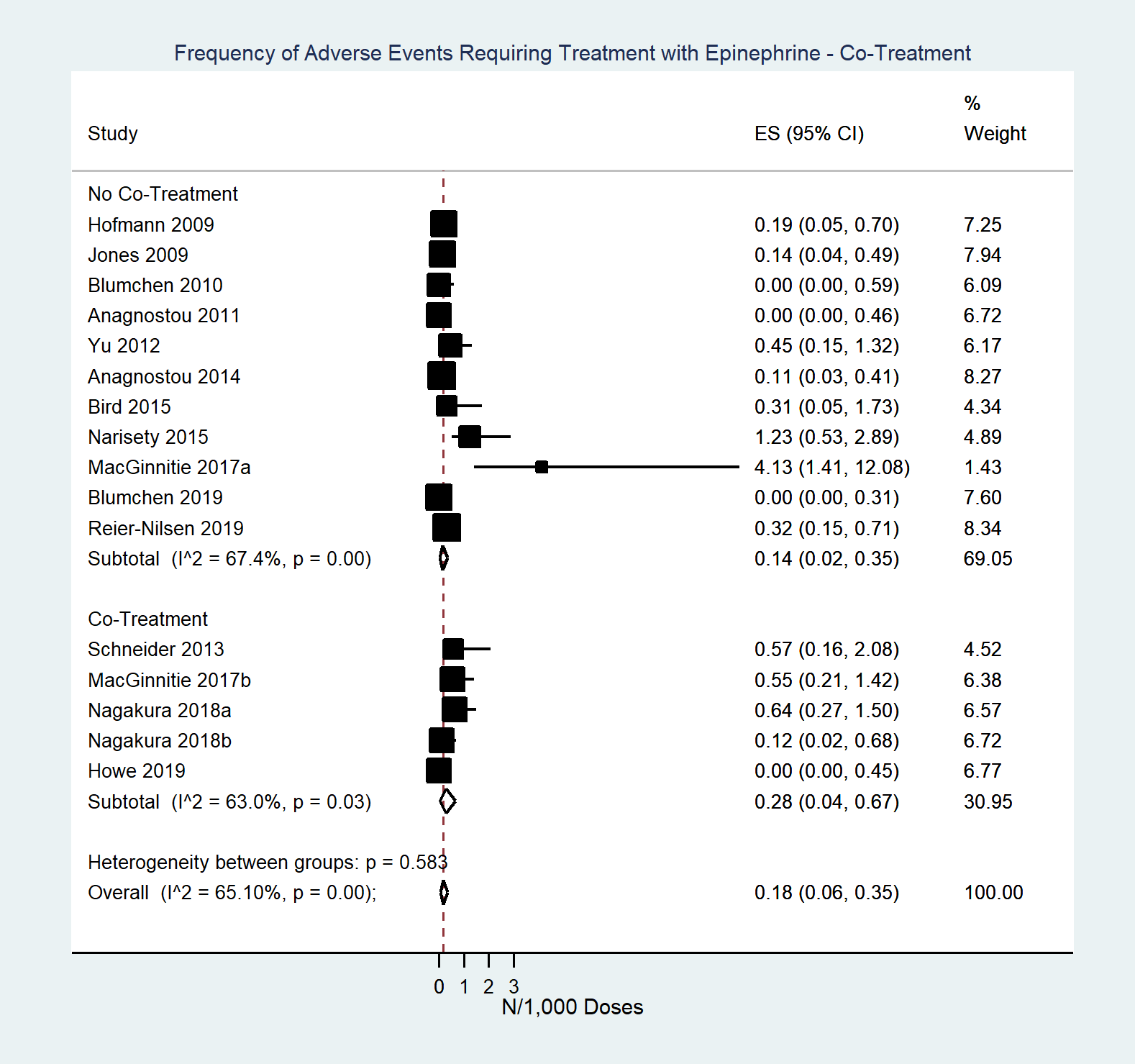
(B) Co-Treatment


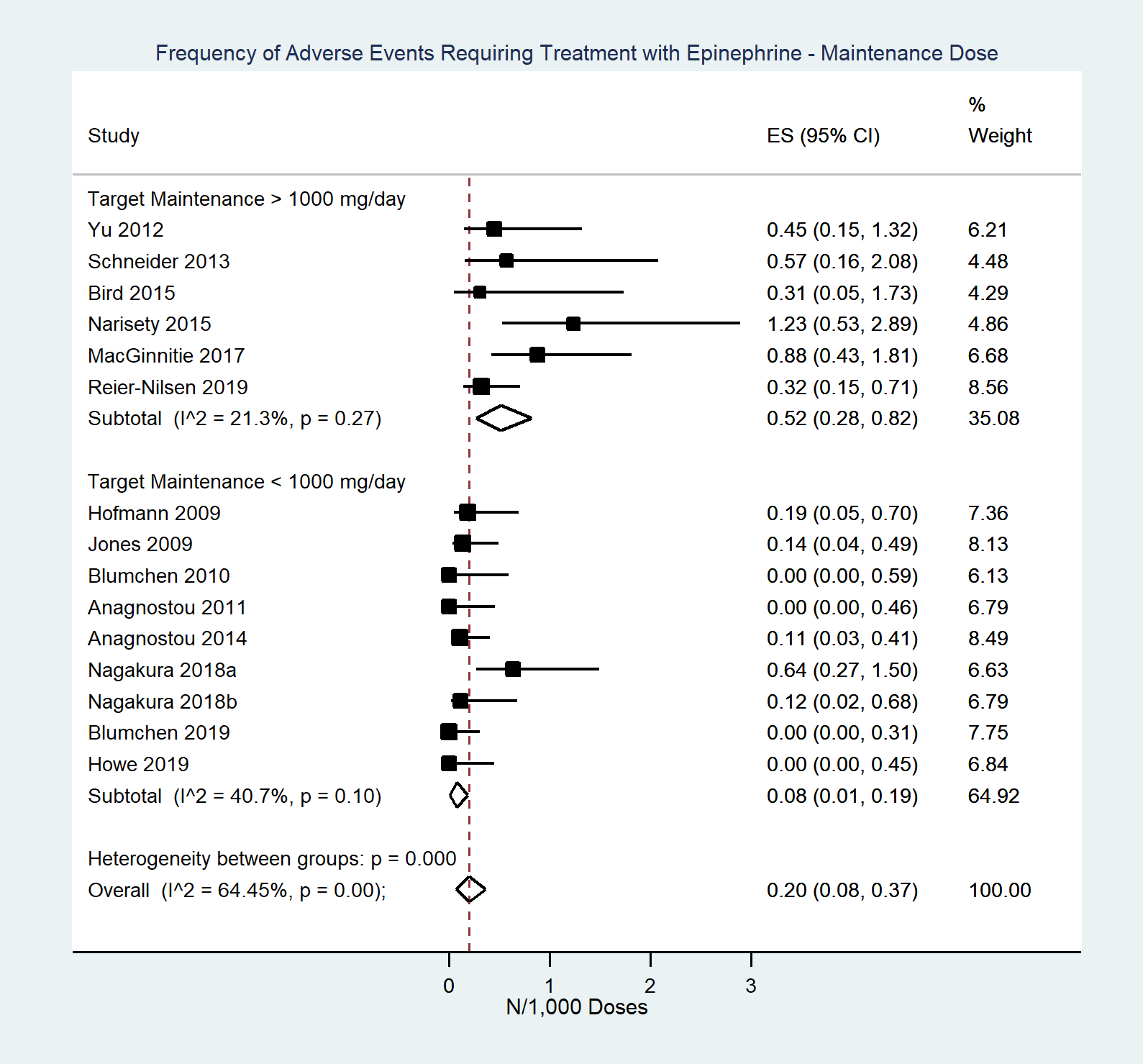
(C) Target Maintenance Dose


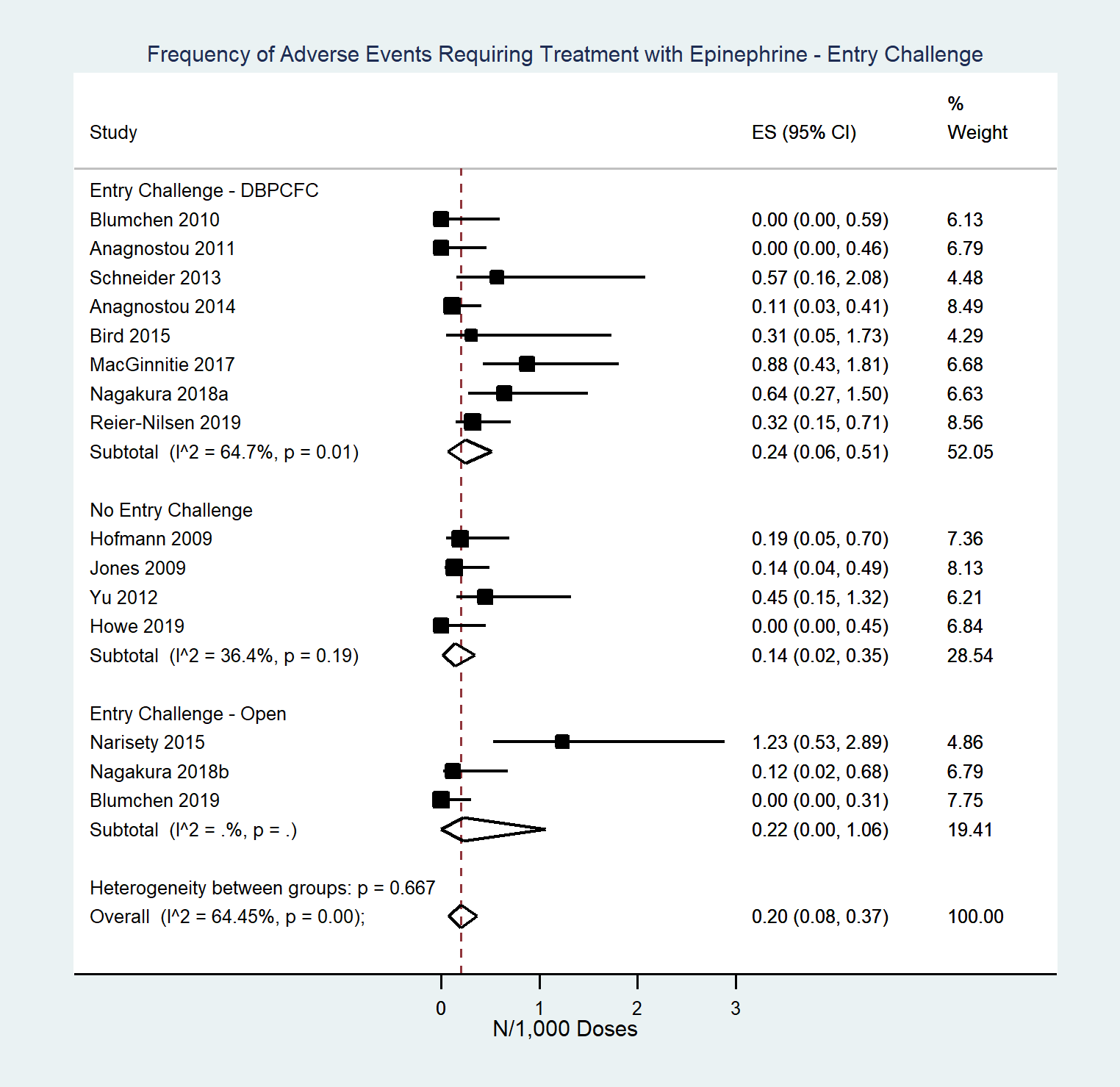
(D) Entry Oral Food Challenge

(E) Baseline Peanut Specific IgE (median)


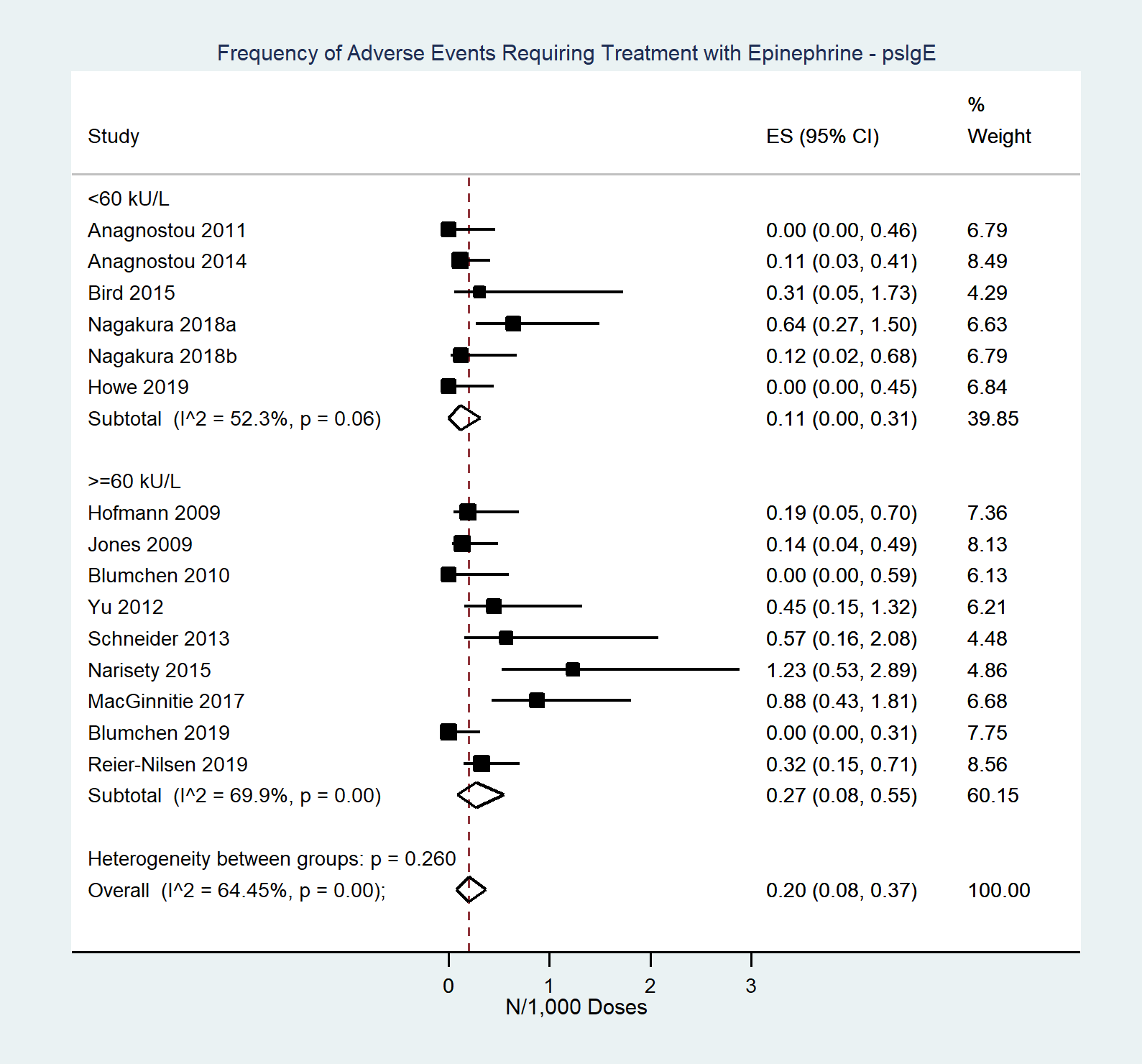


(F) Baseline SPT (median)


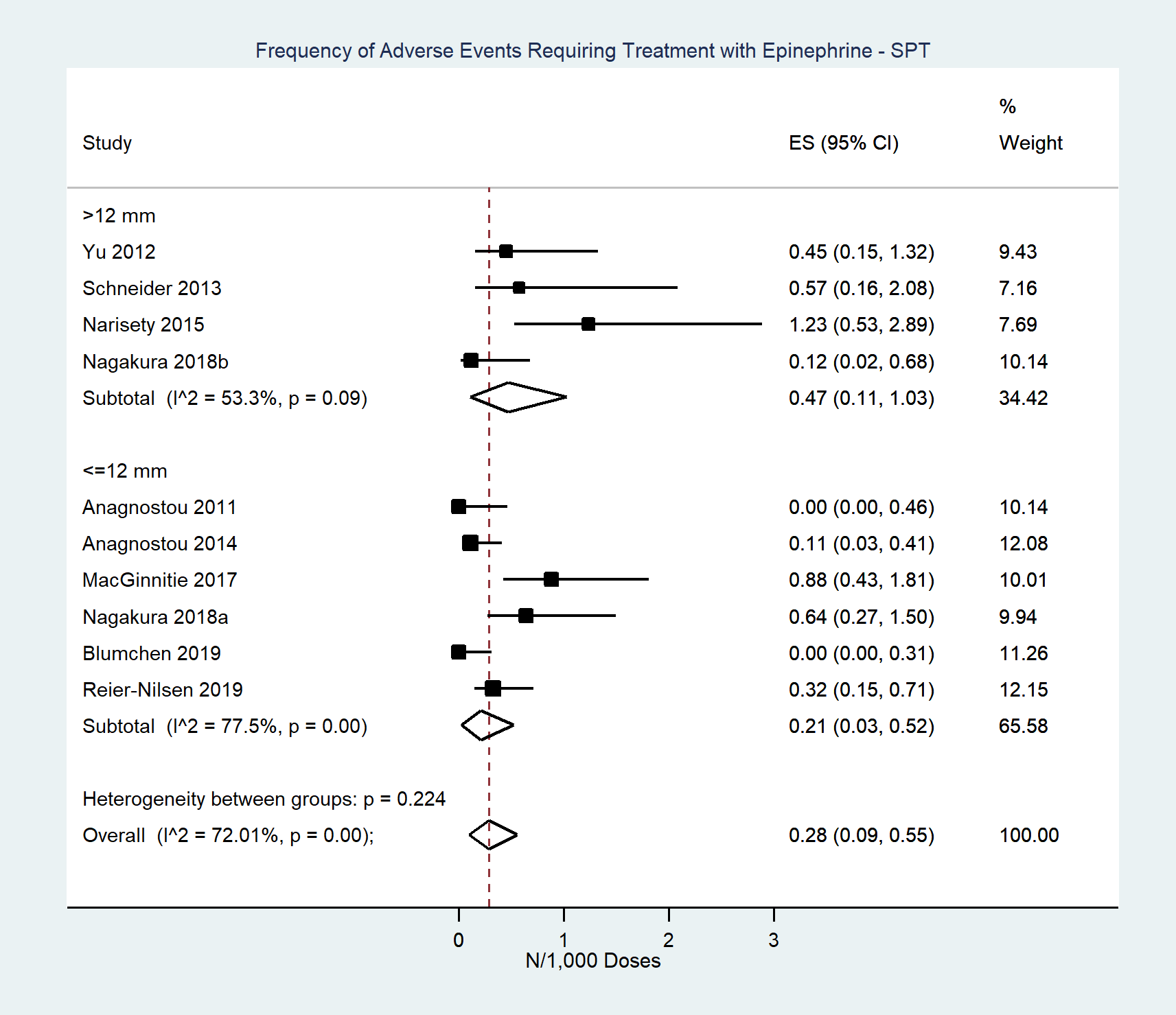


S13 Figure. Meta-regression bubble plots - Risk of Adverse Events Requiring Treatment with Epinephrine

p=0.6371

p=0.3208

p=0.4427

p=0.2439

p=0.0595

p=0.5483


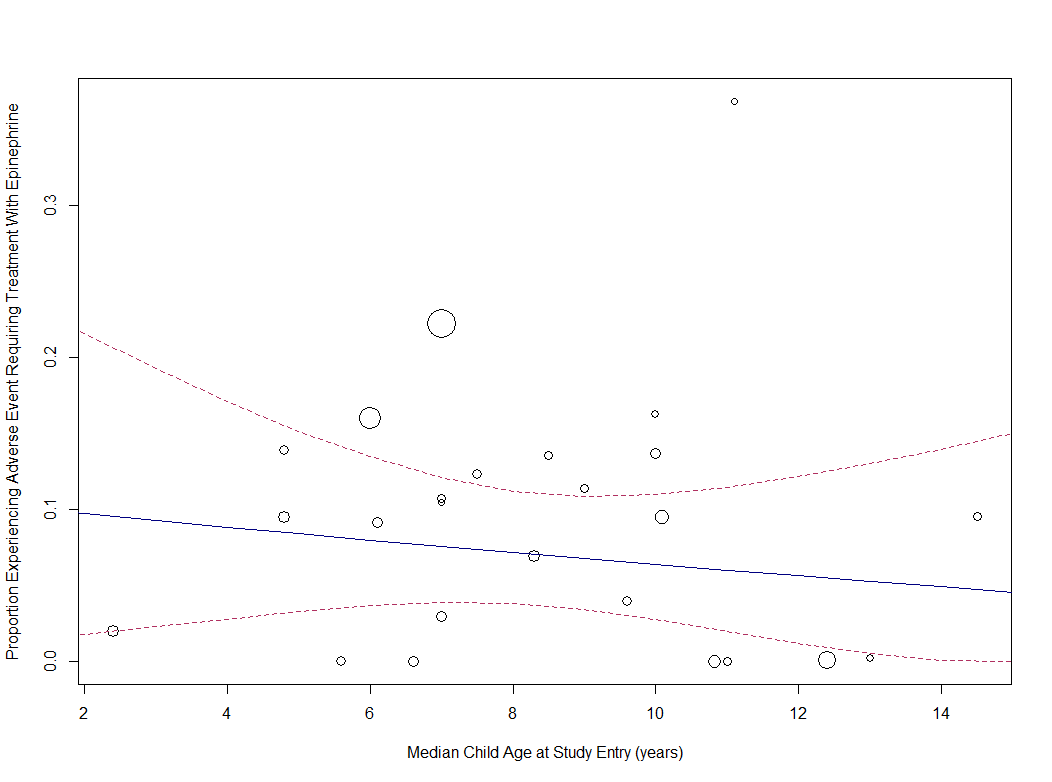

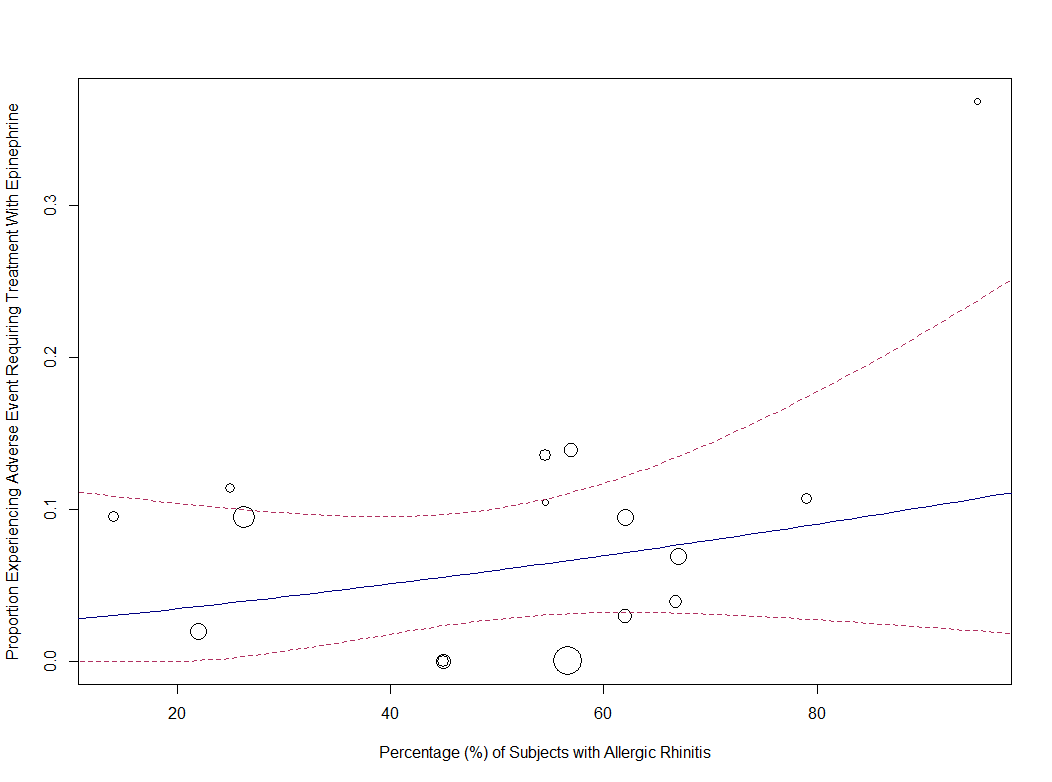

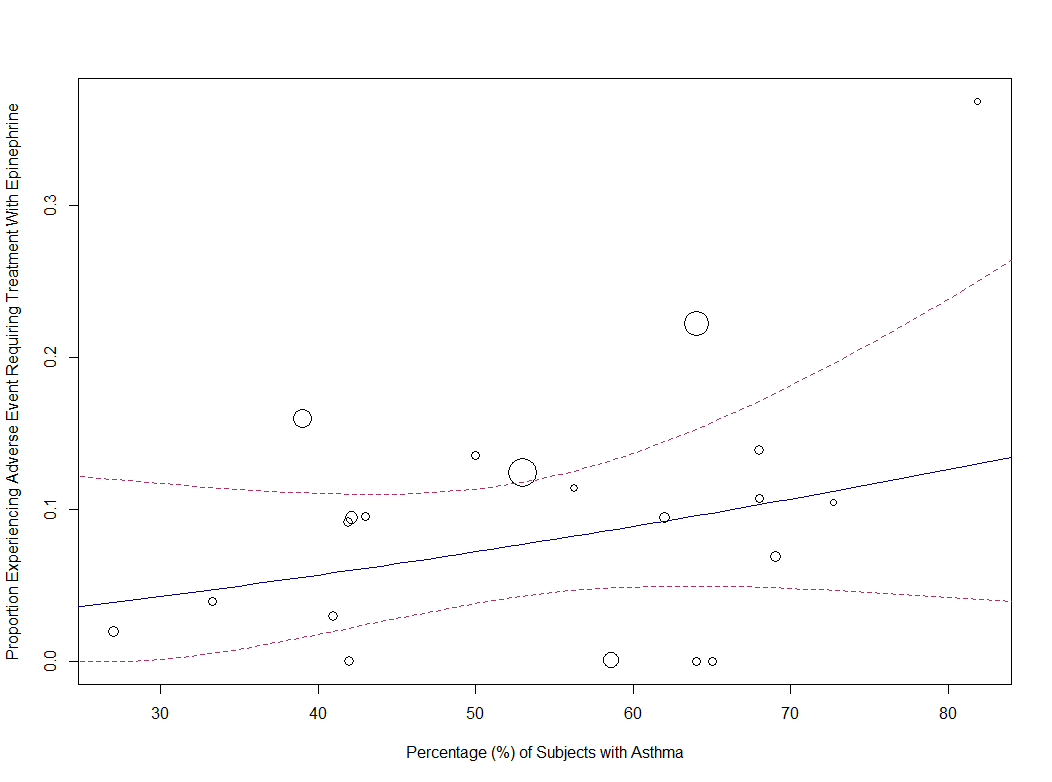

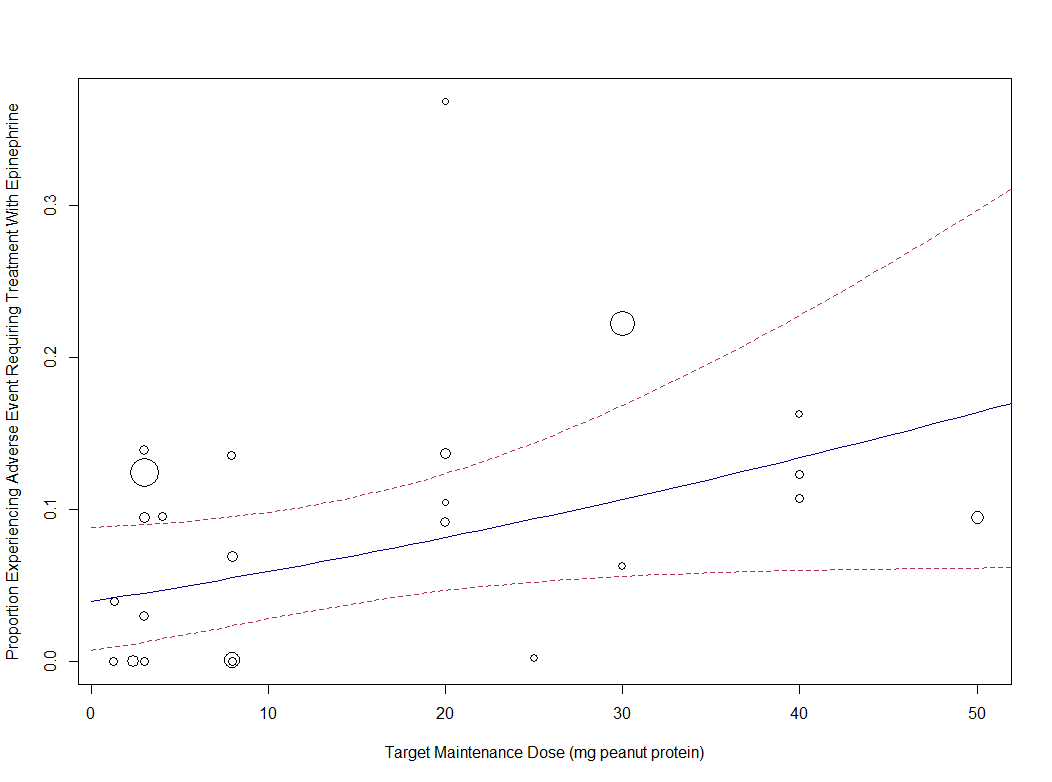

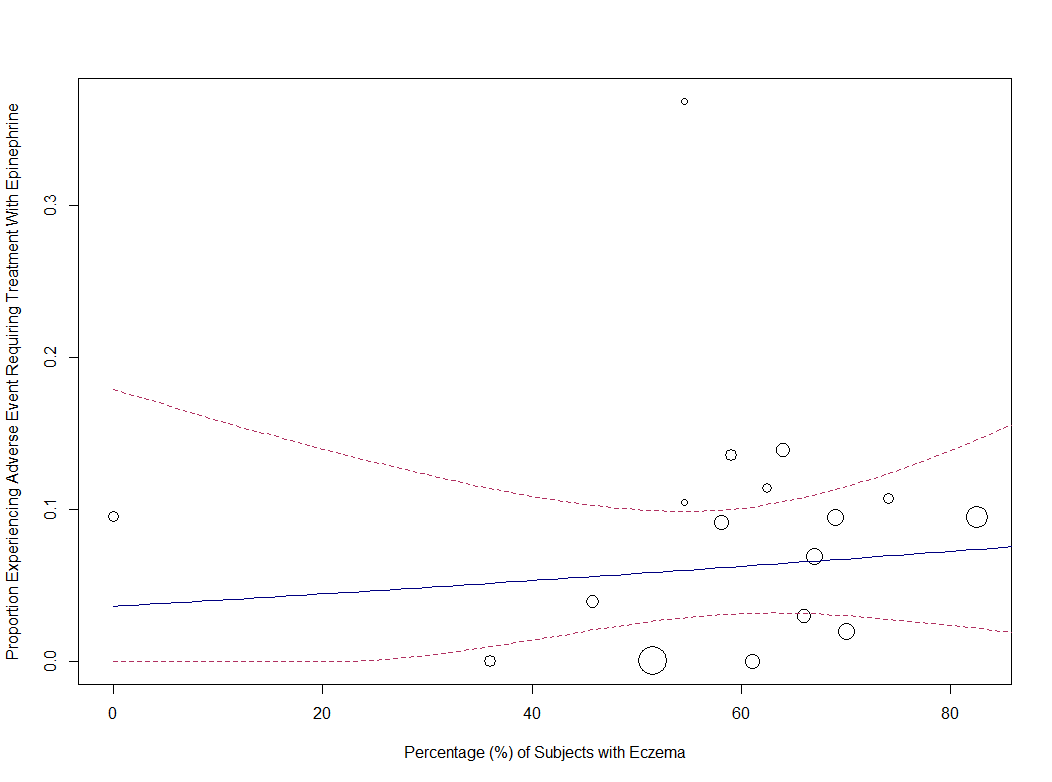

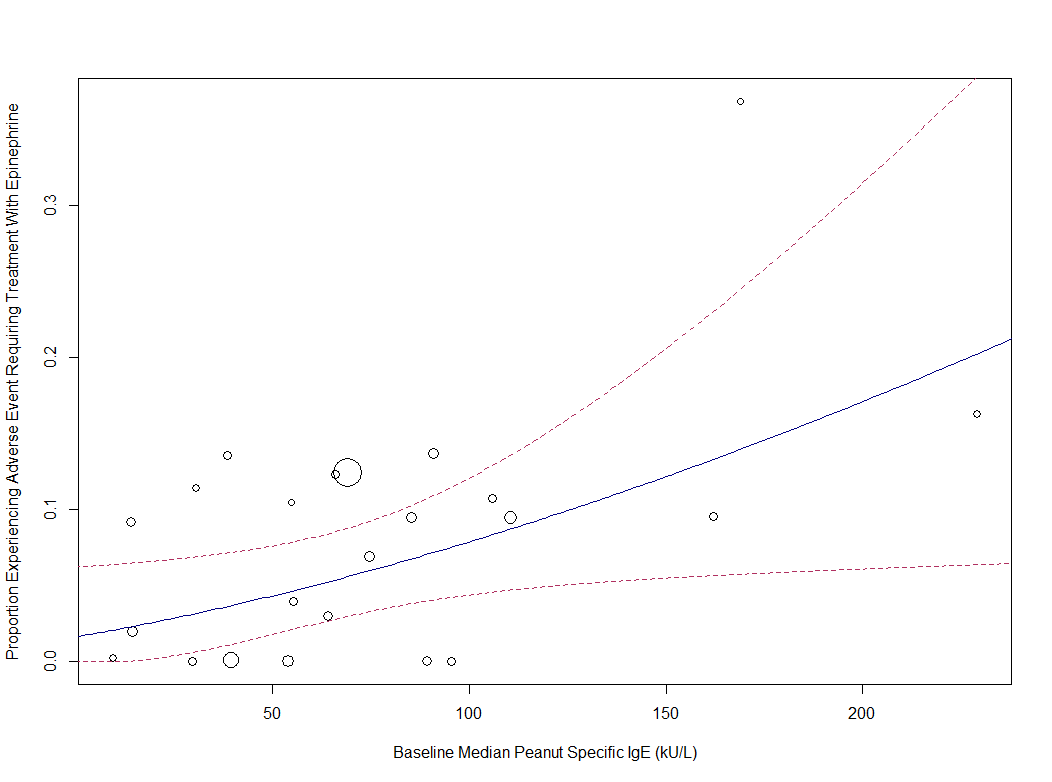

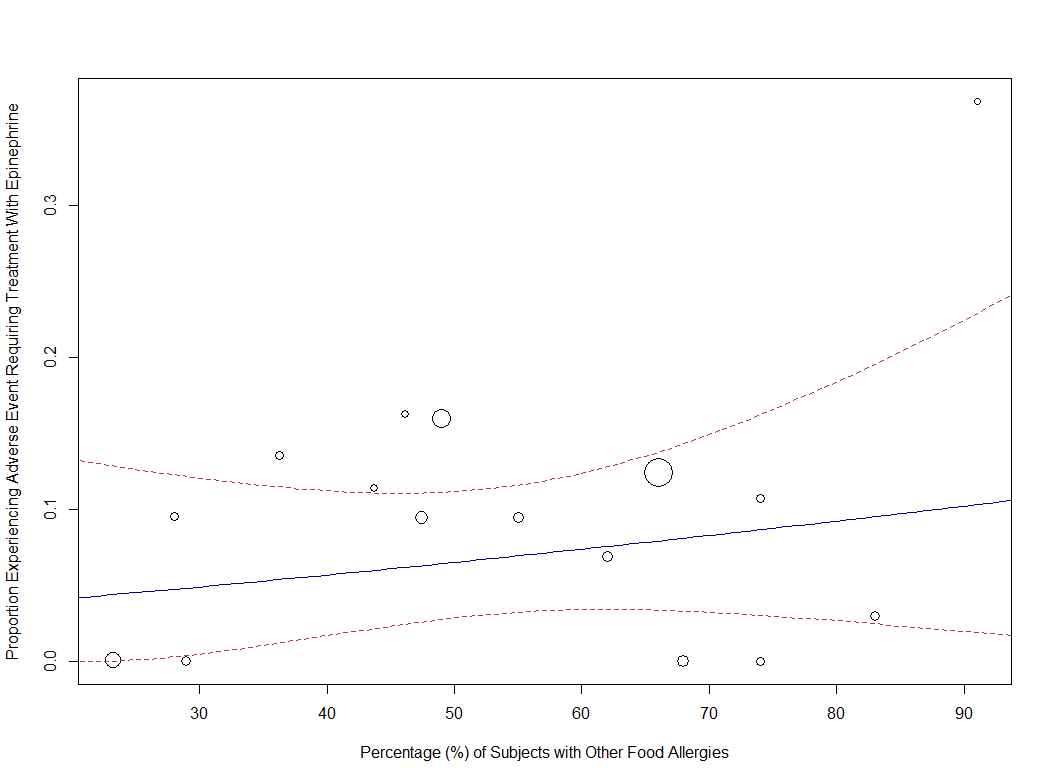

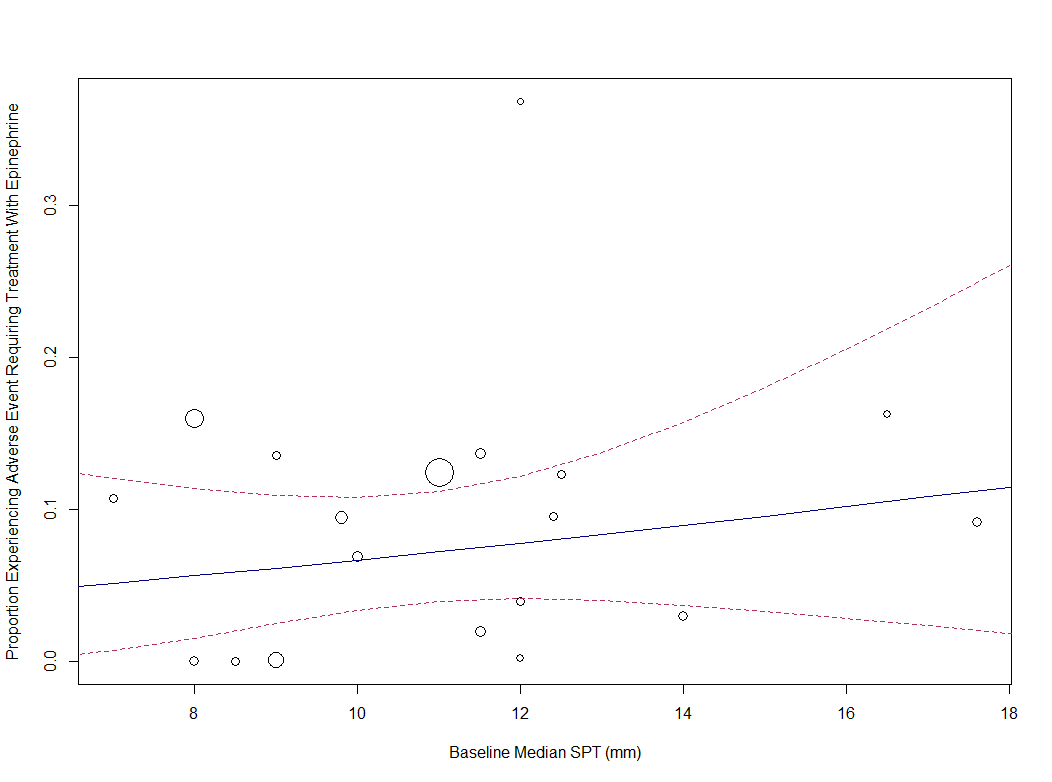


**p=0.0247**

p=0.4334

S14 Figure. Meta-regression bubble plots - Frequency of Adverse Events Requiring Treatment with Epinephrine

p=0.0636

p=0.3391

p=0.4524

p=0.5093

**p=0.0243**

**p=0.0430**


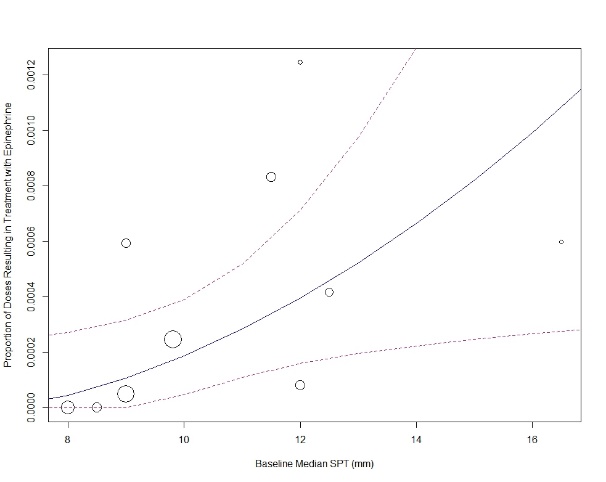

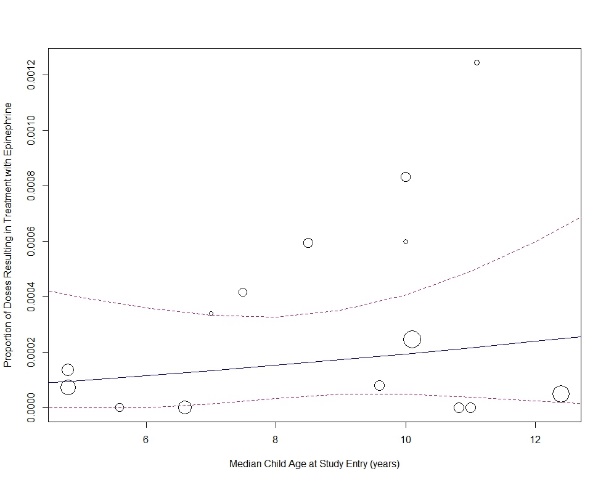

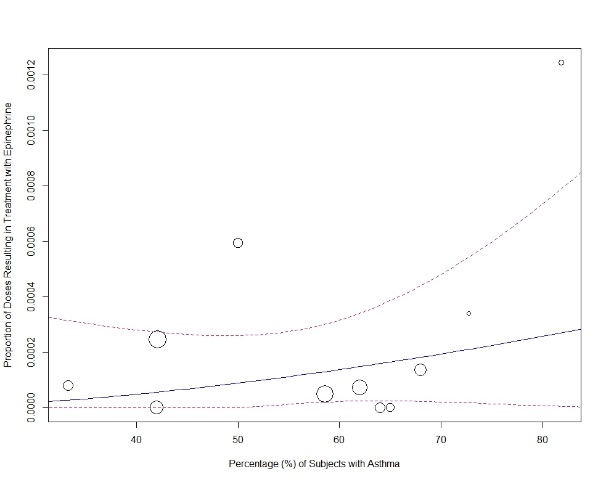

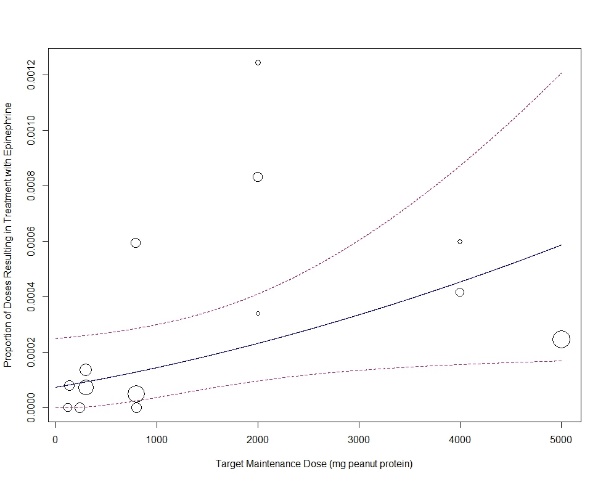

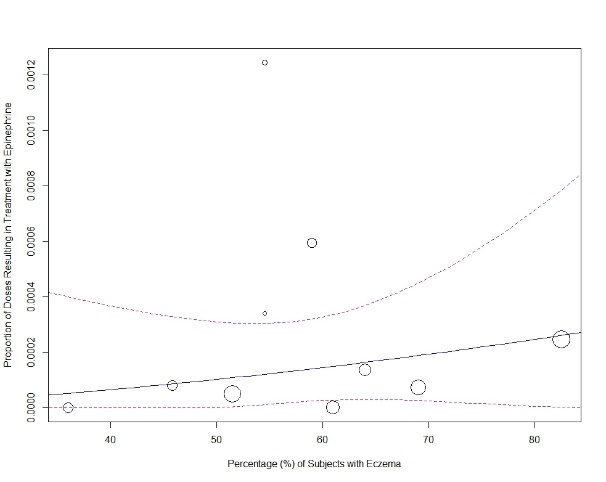

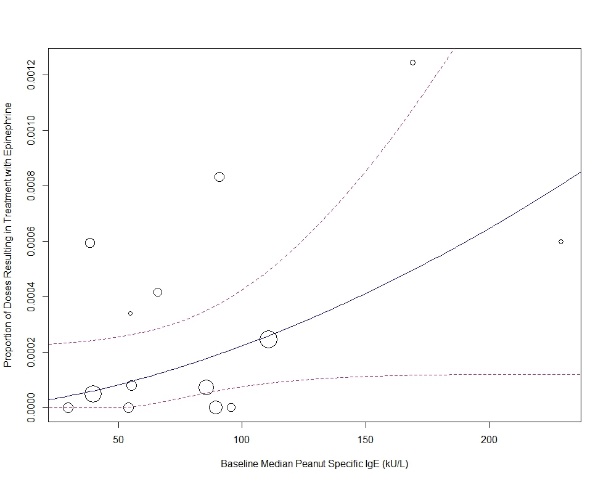

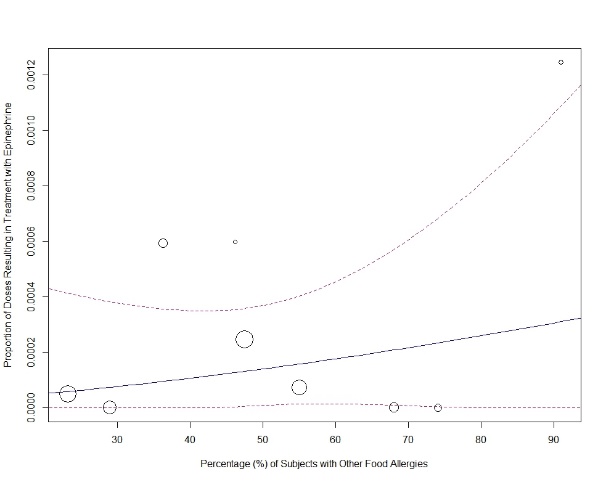

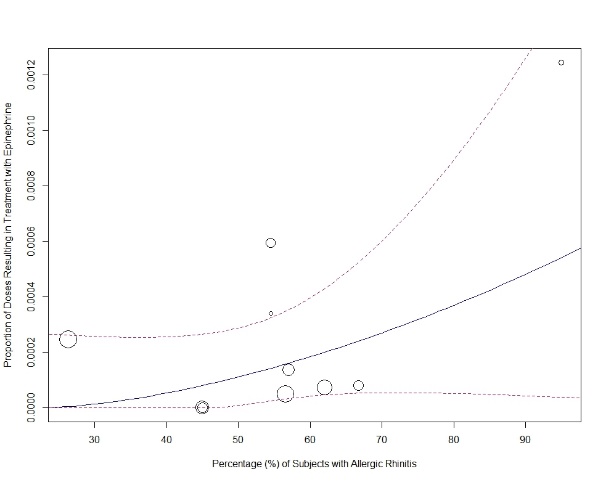


p=0.4550

p=0.1413

S15 Figure. Forest plots - Able to Reach Target Maintenance Dose

(A) Rush Phase


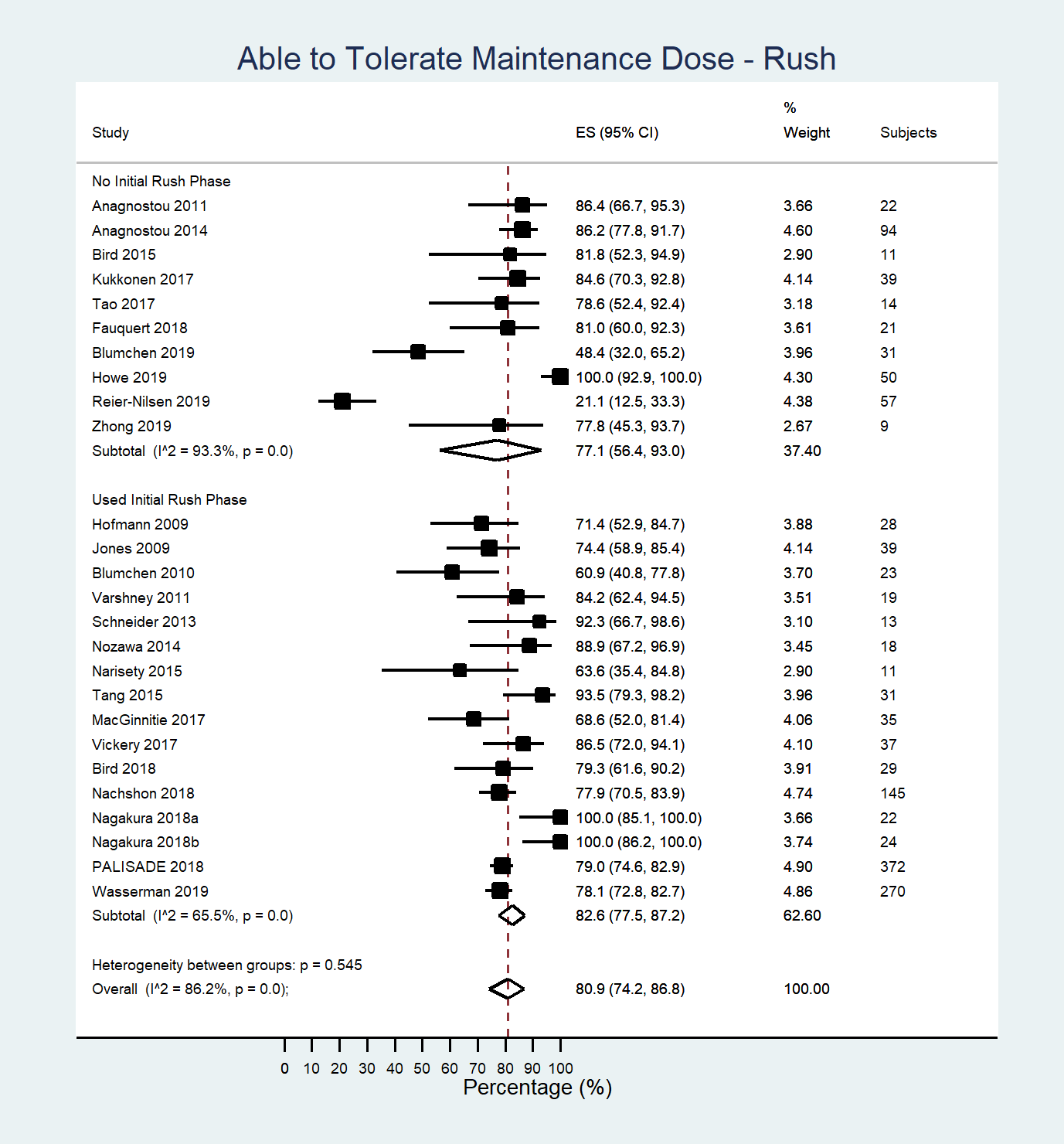


(B) Co-Treatment


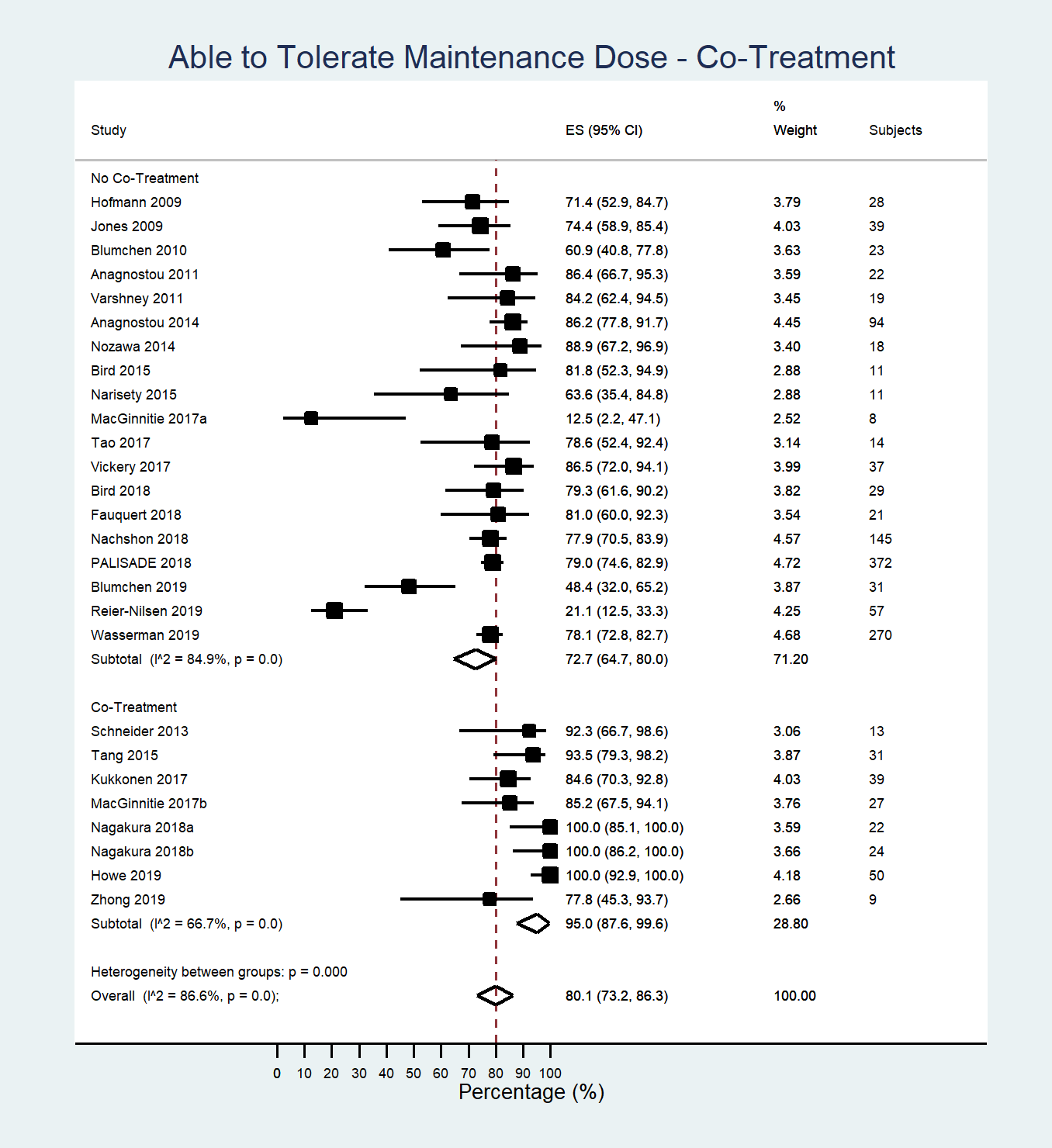


(C) Co-Treatment Type


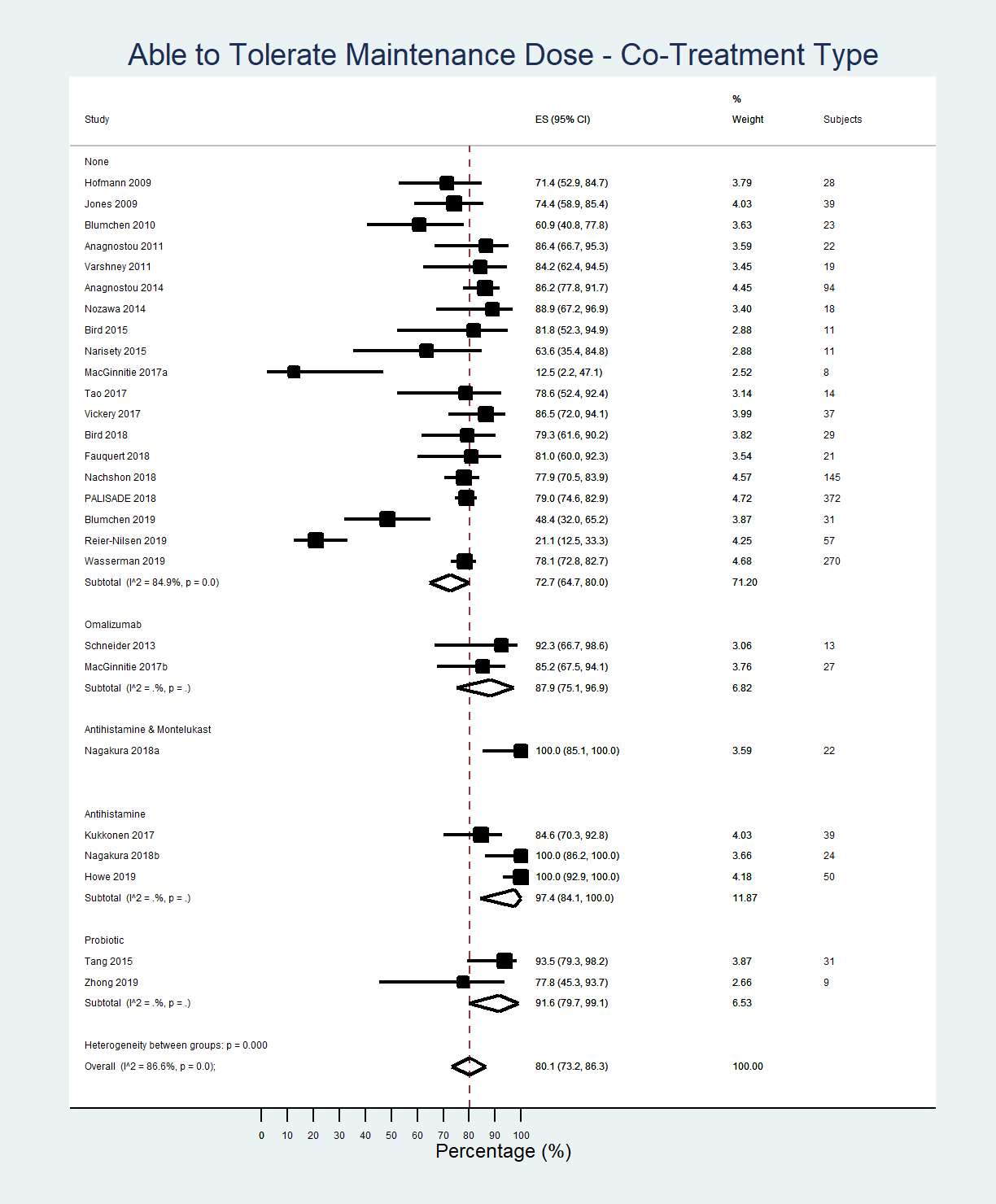


(D) Target Maintenance Dose


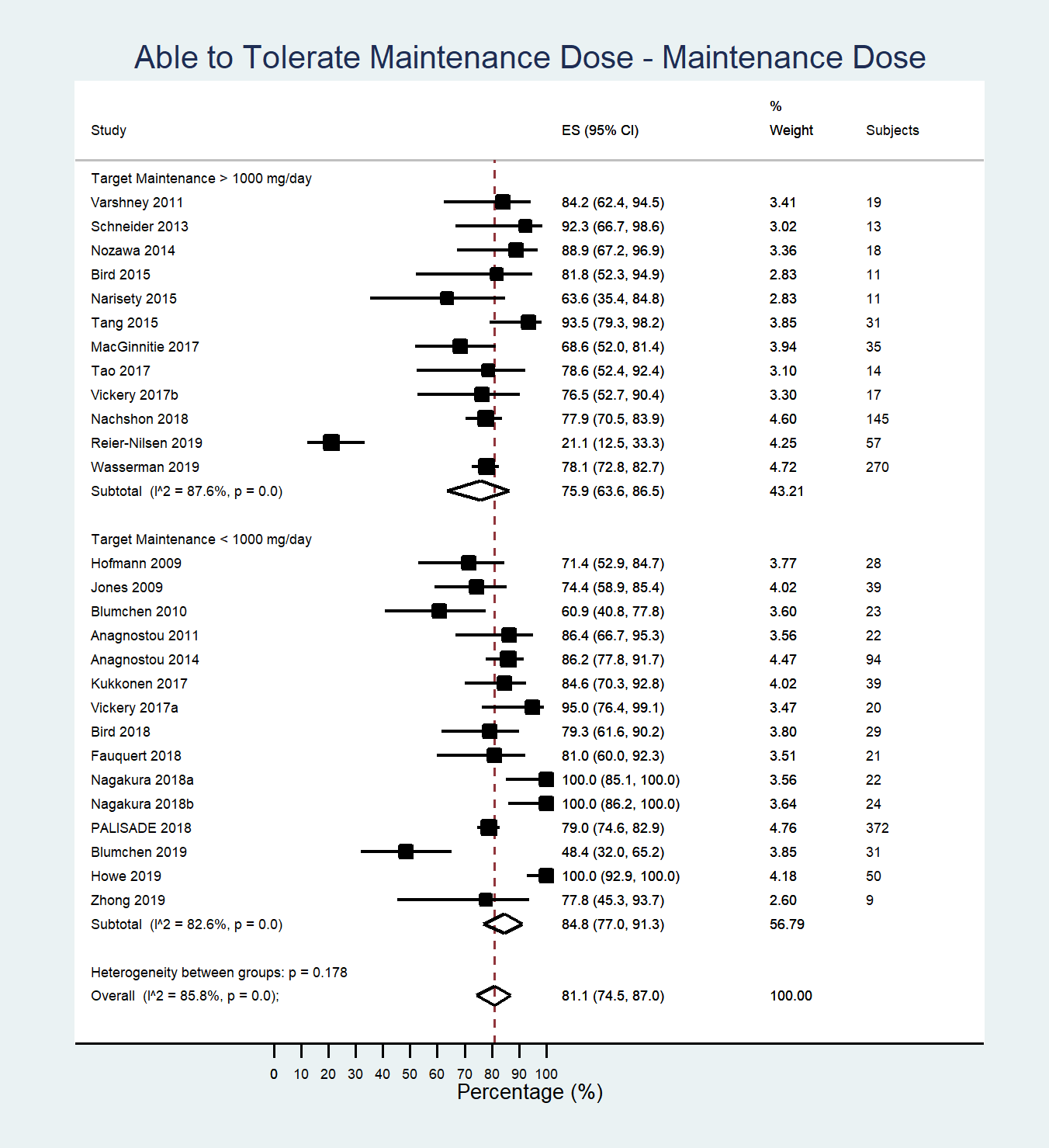


(E) Entry Oral Food Challenge


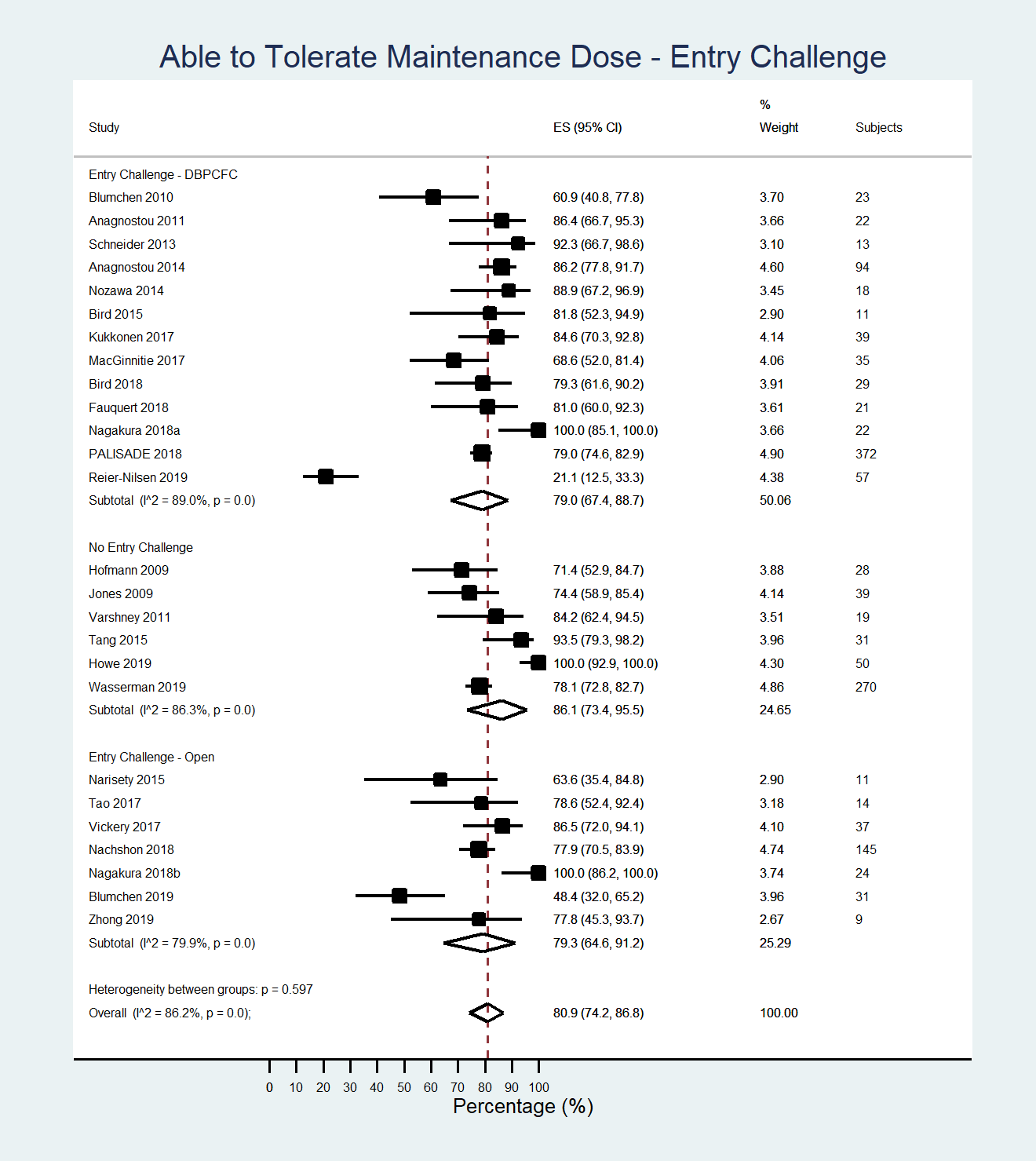


(F) Baseline Peanut Specific IgE (median)


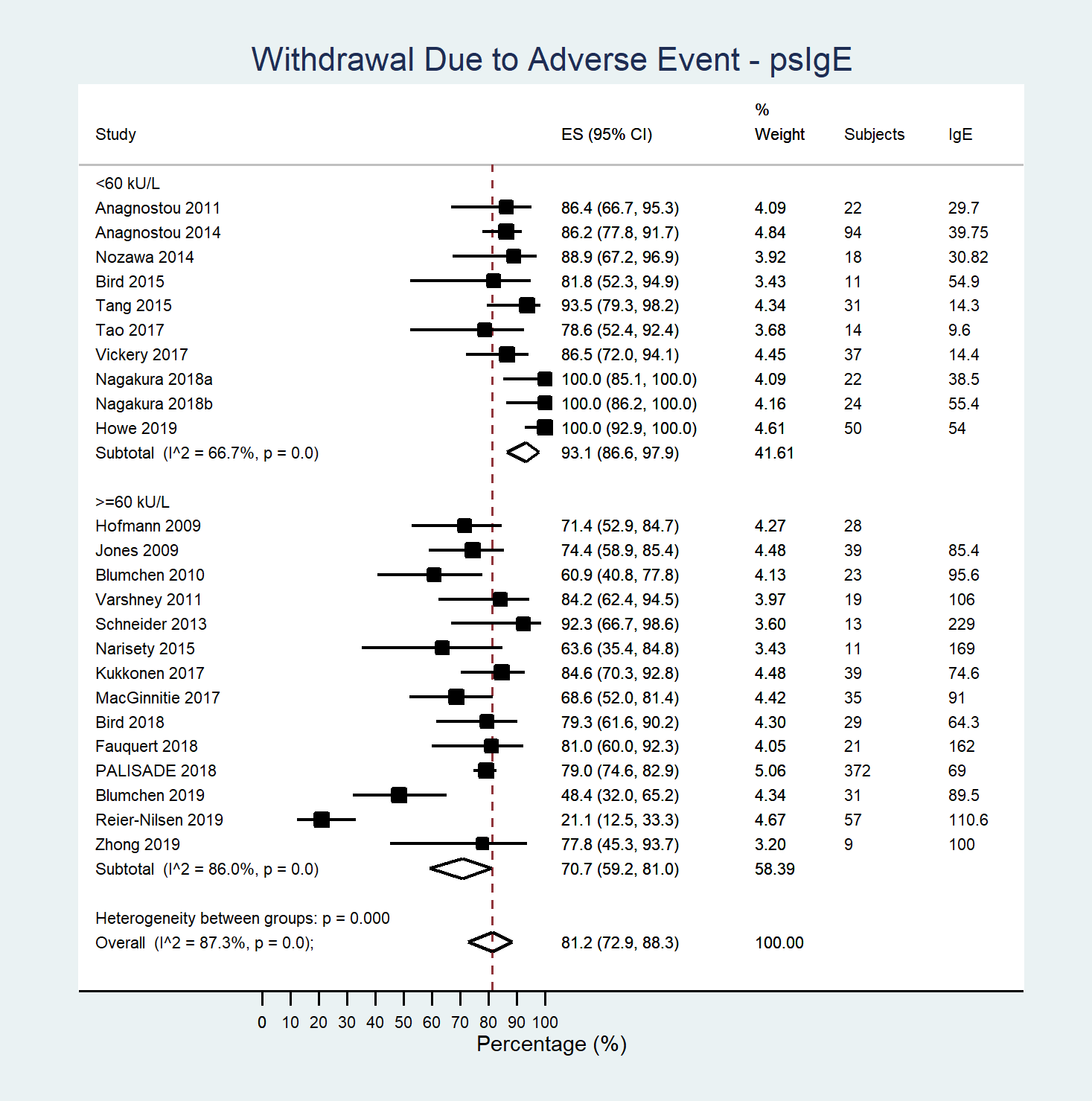


(G) Baseline SPT (median)


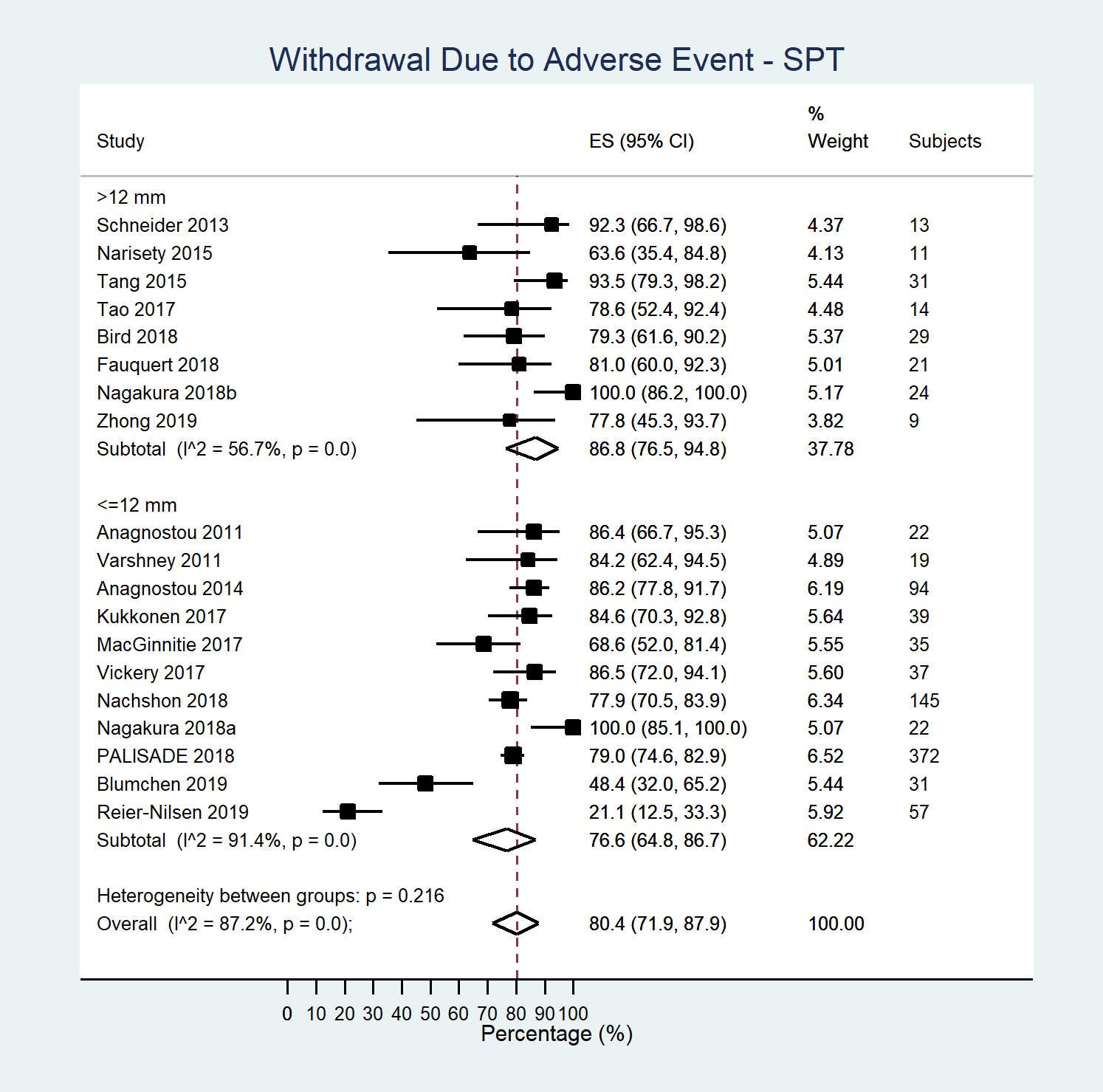


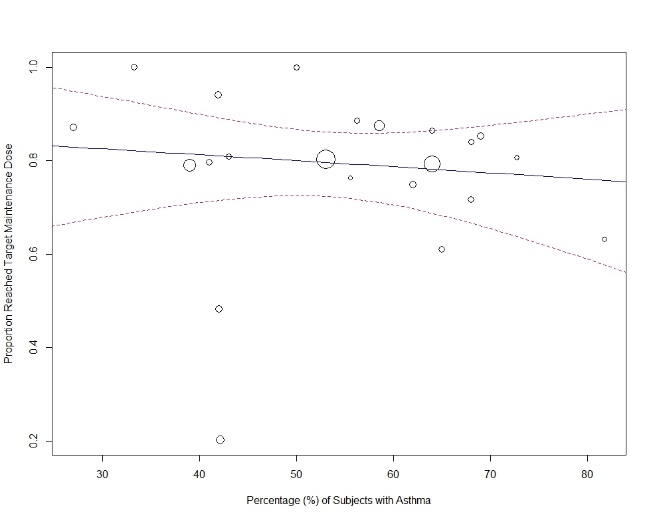

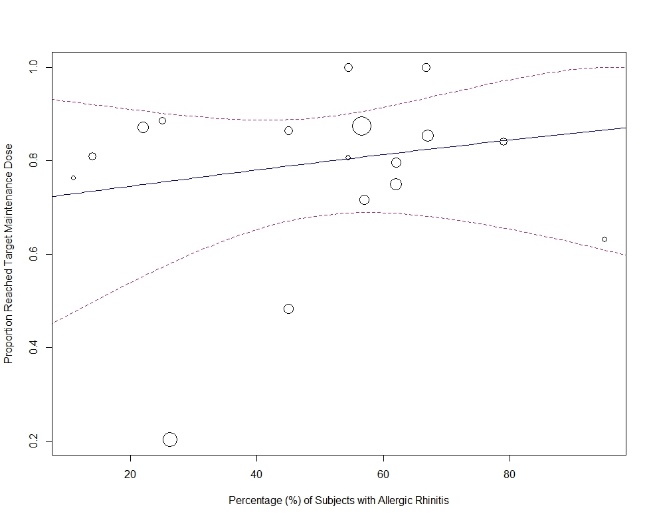

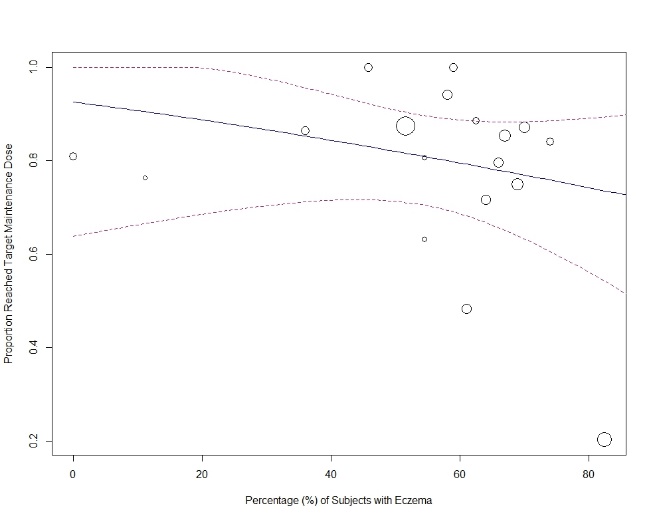

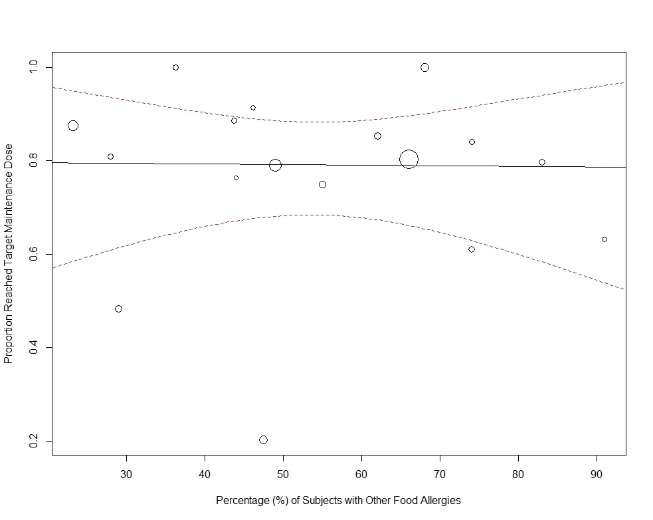

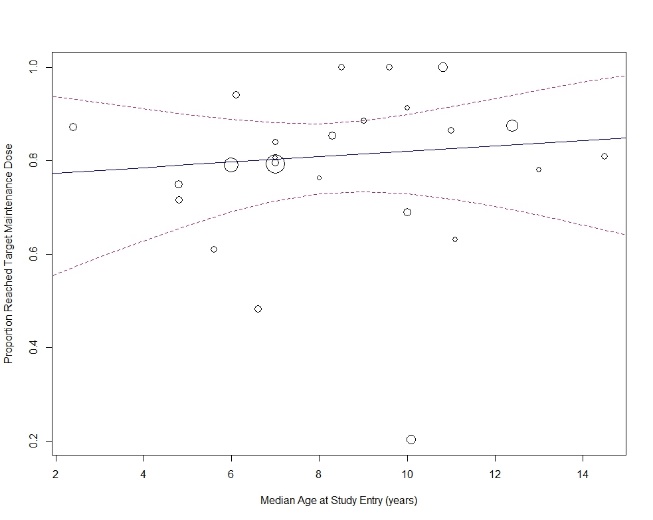

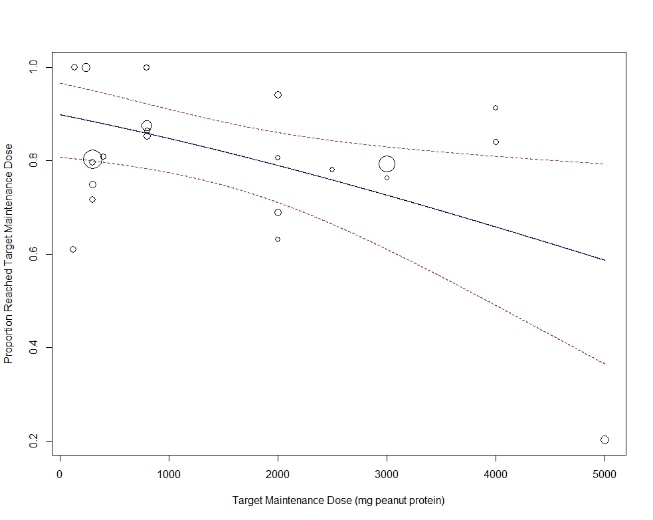

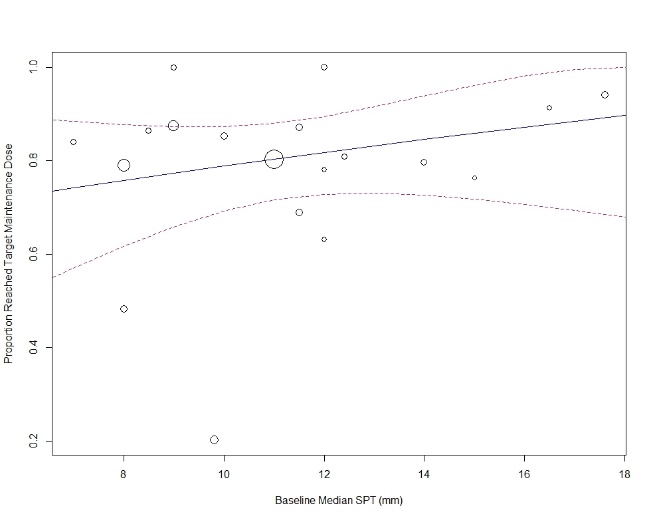

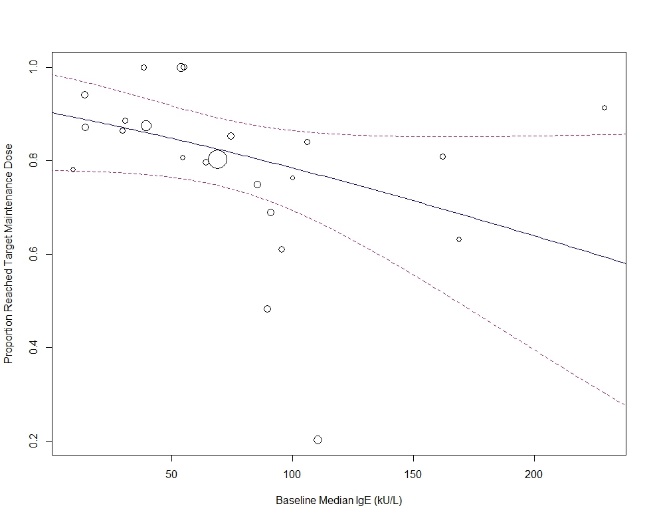
S16 Figure. Meta-regression bubble plots - Able to Reach Target Maintenance Dose

p=0.9616

p=0.6177

p=0.6693

p=0.3255

**p=0.0088**

p=0.0881

p=0.3196

p=0.5028

S17 Figure. Forest plots - Pass Supervised Exit Oral Food Challenge

(A) Rush Phase


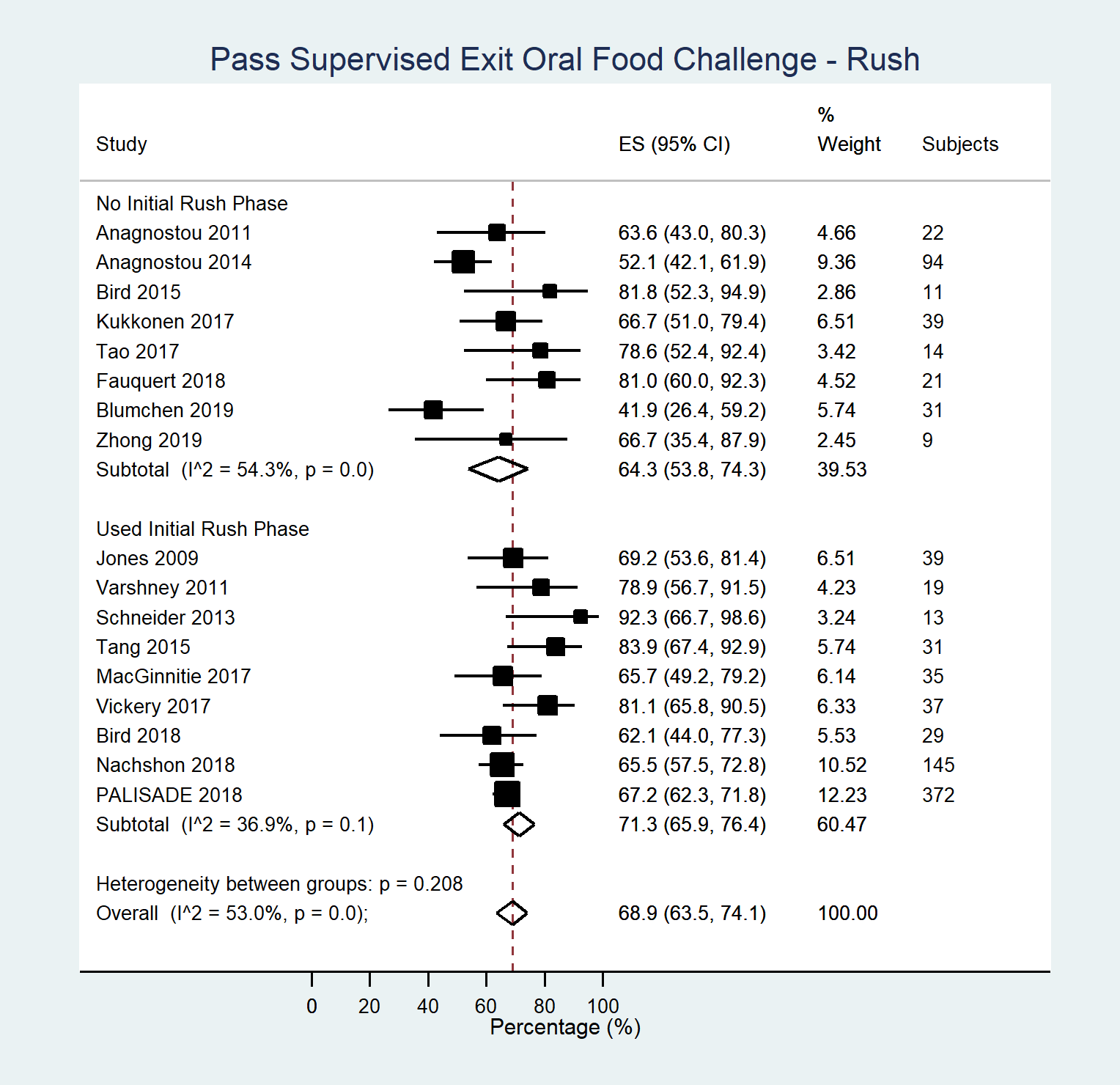


(B) Co-Treatment


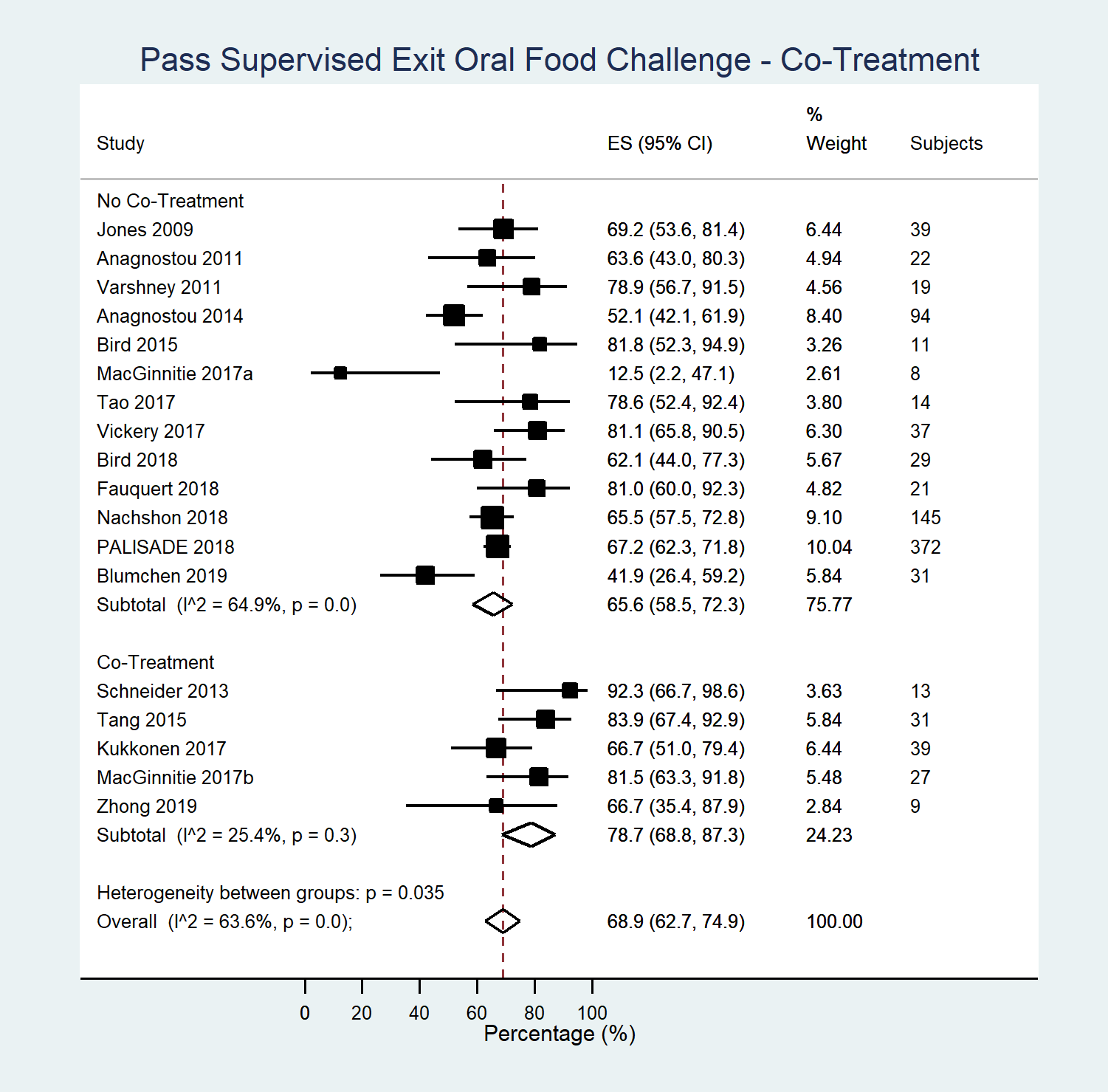


(C) Co-Treatment Type


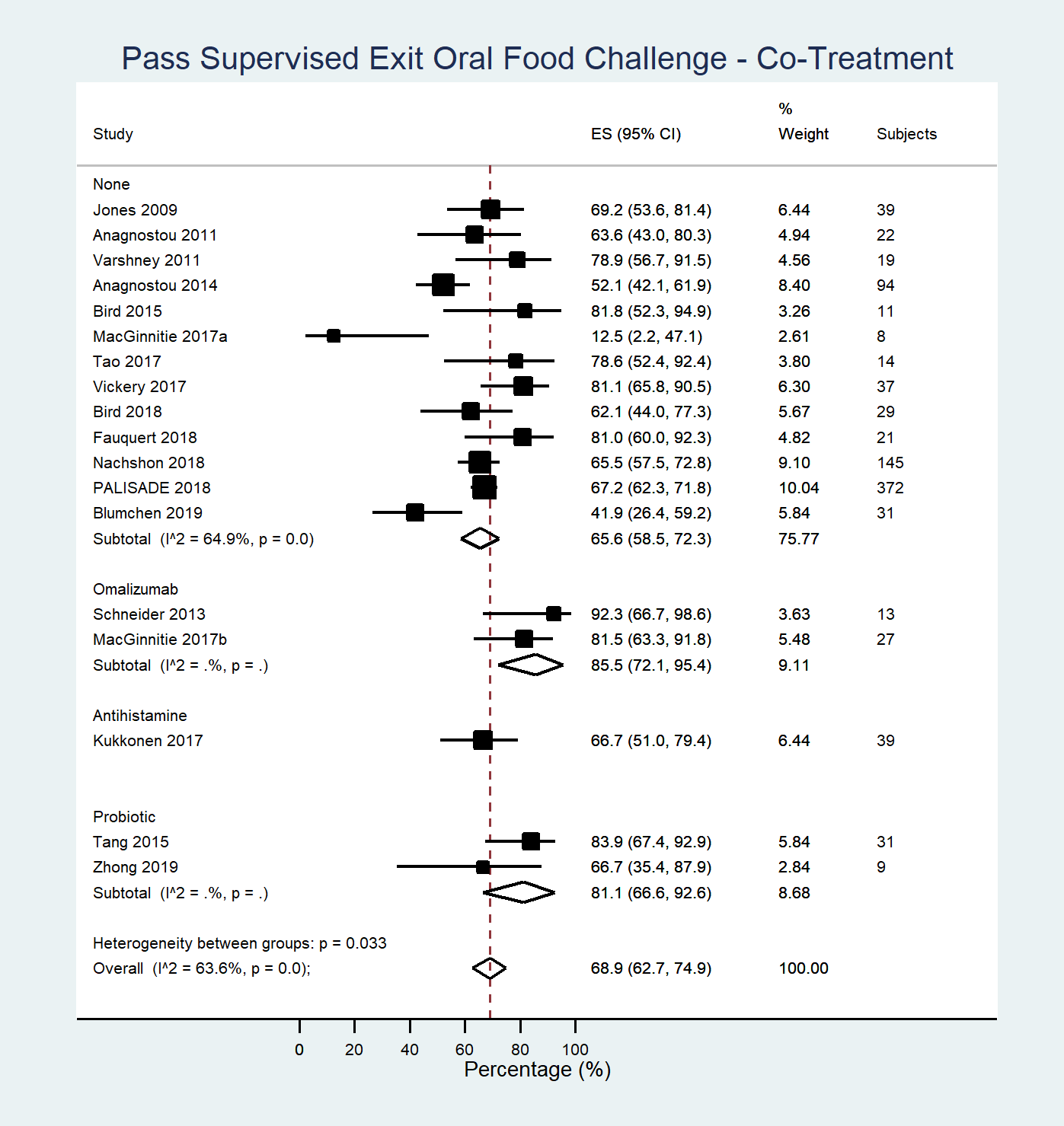


(D) Target Maintenance Dose


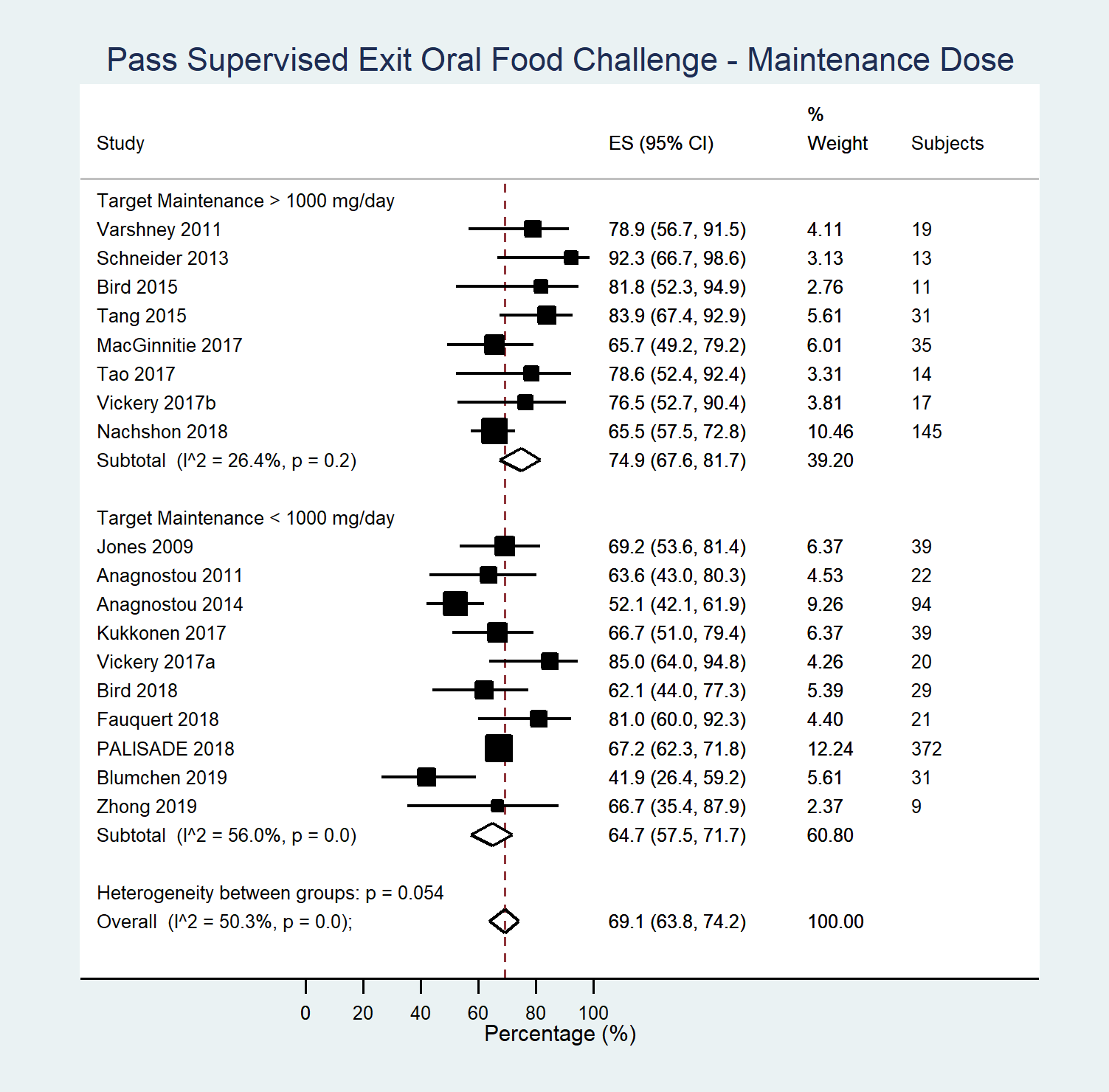


(E) Entry Oral Food Challenge


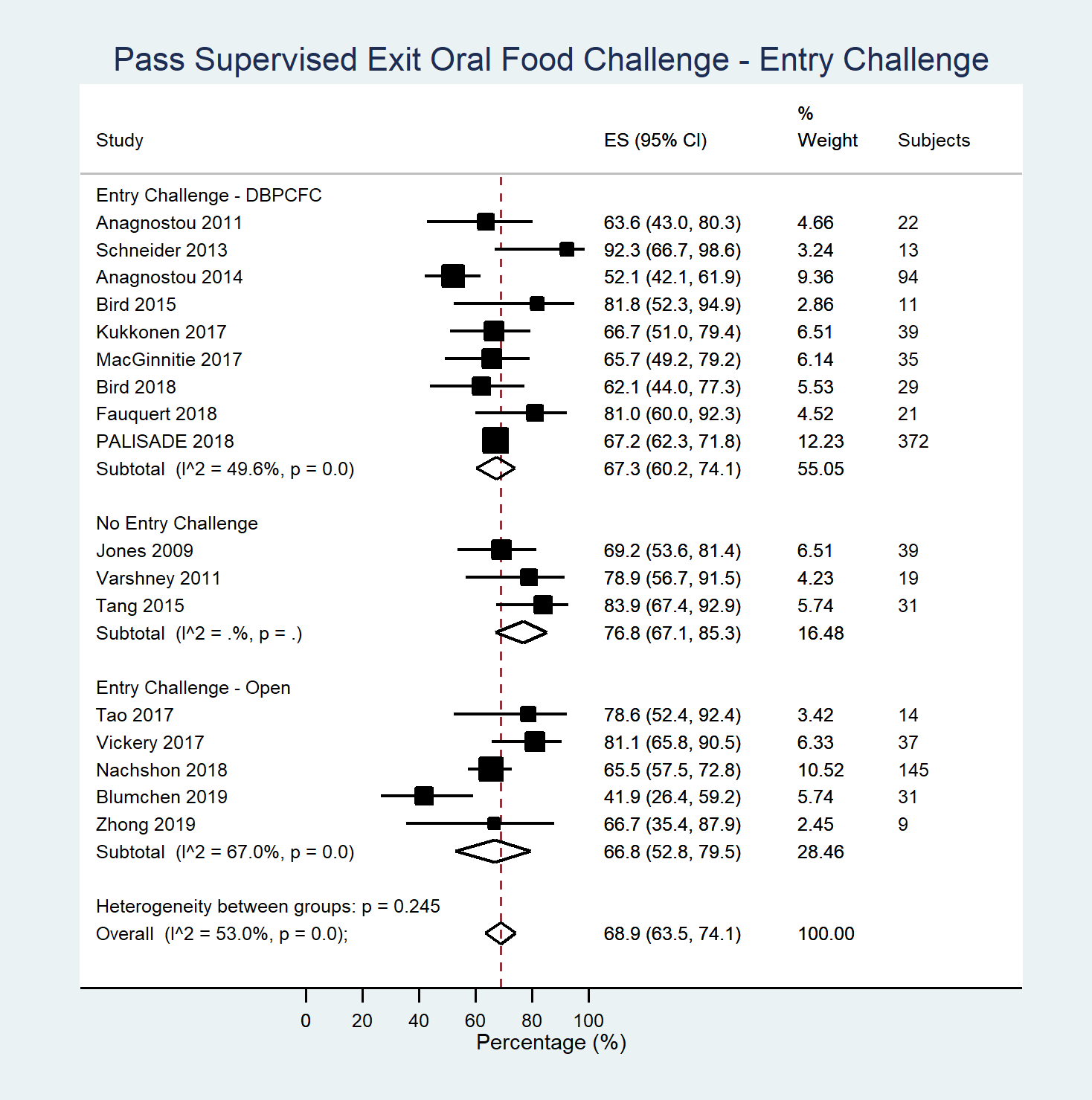


(F) Baseline Peanut Specific IgE (median)


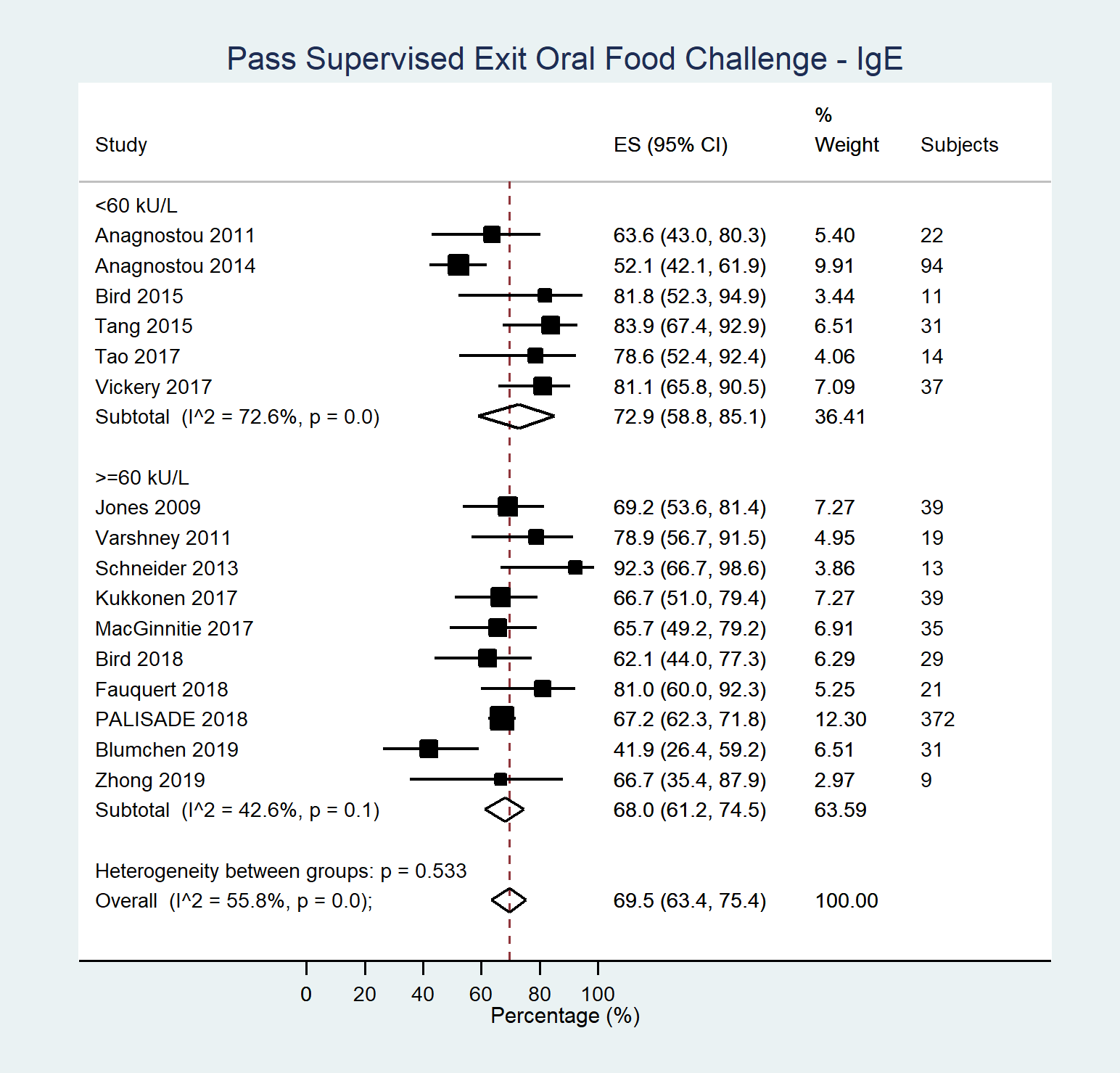


(G) Baseline SPT (median)


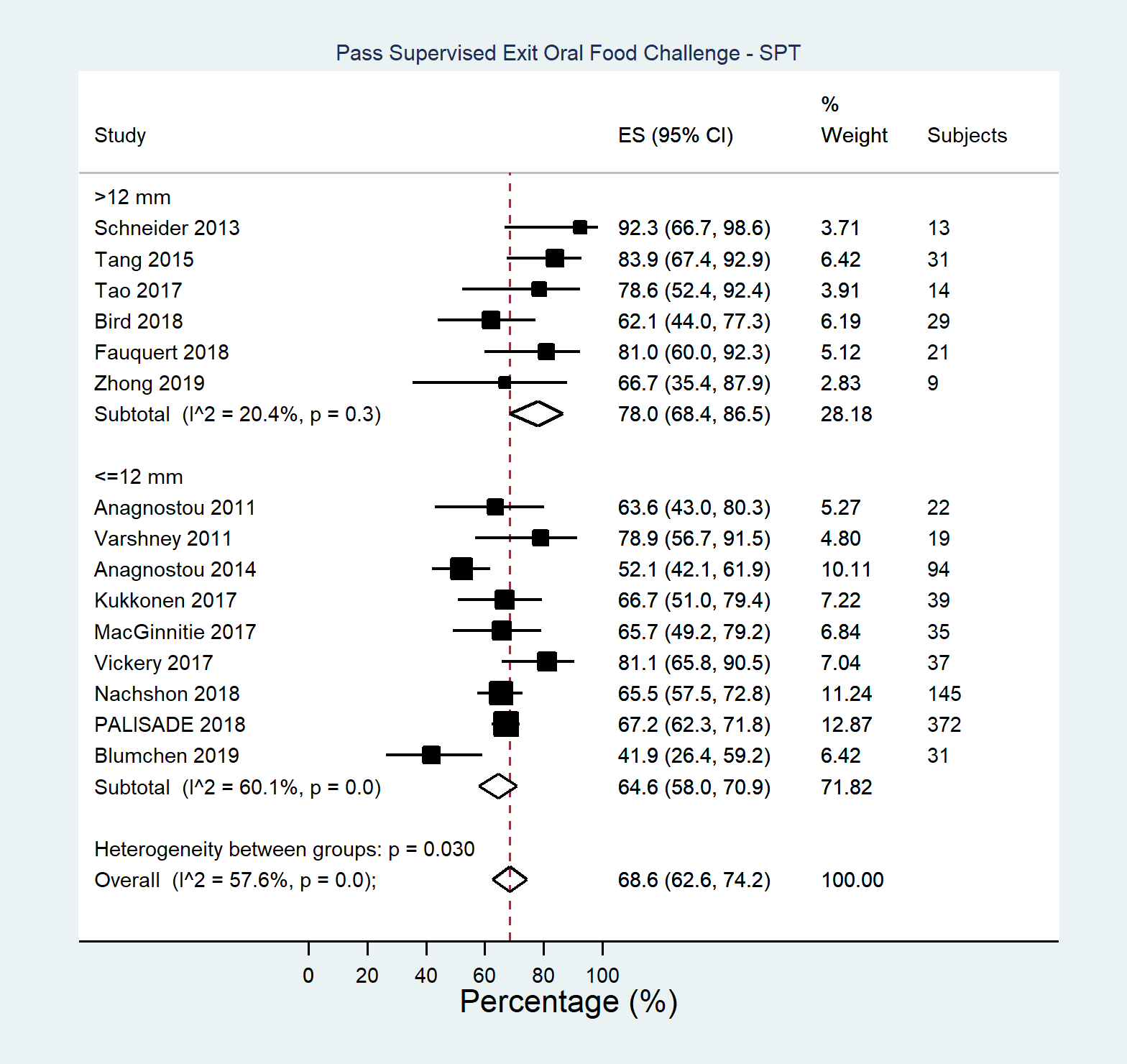


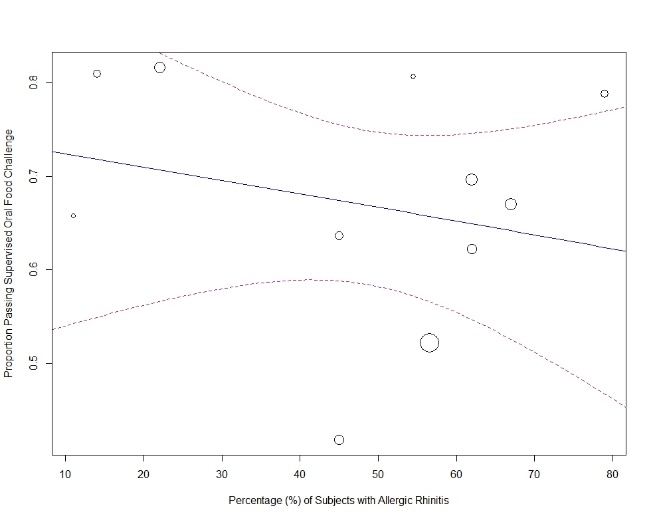

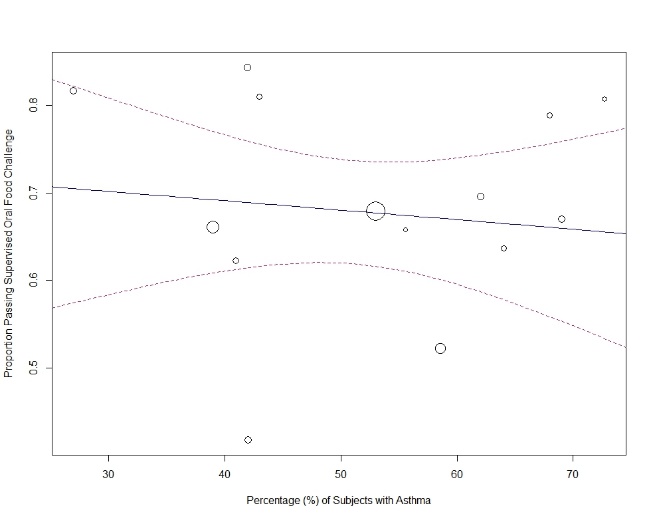

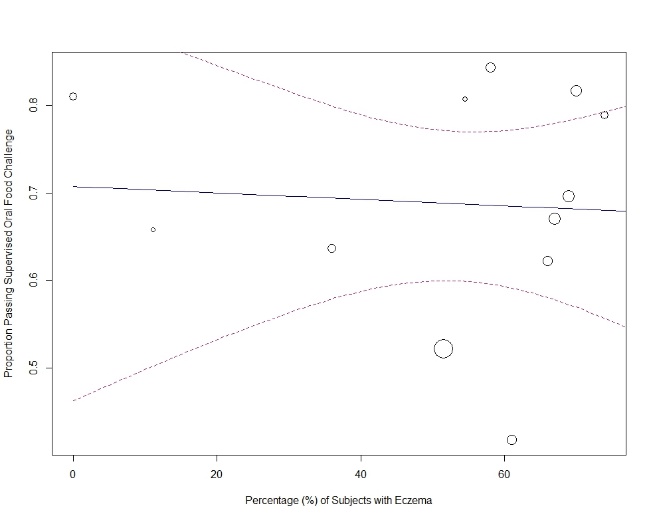
S18 Figure. Meta-regression bubble plots - Completion of Supervised Oral Food Challenge

**p=0.0058**

p=0.4273

p=0.0618

p=0.6500

p=0.8581

p=0.4893

p=0.6453

p=0.1657

S19 Figure. Forest plot for successful completion of supervised exit oral food challenge according to per-protocol analysis

| **S2 Table. Summary of characteristics of included studies** | | |
| --- | --- | --- |
| **Characteristic** | **Studies (n)** | **Patients (n)** |
| Total | 27 | 1488 |
| Rush Phase |  |  |
| Yes | 17 (63) | 1140 |
| No | 10 (37) | 348 |
| Co-Treatment^¥^ |  |  |
| Yes | 9 (32) | 215 |
| No | 19 (68) | 1273 |
| Peanut OIT Blinding |  |  |
| Yes | 6 (22) | 503 |
| No | 21 (78) | 985 |
| Maintenance Dose  (mg/day)^†^ |  |  |
| < 1000 | 15 (54) | 823 |
| ≥ 1000 | 13 (46) | 665 |
| Entry OFC |  |  |
| DBPCFC | 13 (48) | 756 |
| Open | 7 (26) | 271 |
| None | 7 (26) | 461 |
| Baseline psIgE (kU/L) |  |  |
| <60 | 10 (37) | 323 |
| ≥60 | 15 (56) | 750 |
| Not reported | 2 (7) | 415 |
| Baseline SPT (mm) |  |  |
| <12 | 11 (41) | 873 |
| ≥12 | 9 (33) | 176 |
| Not reported | 7 (26) | 439 |
| ^¥^ - One study randomised children to receive co-treatment with omalizumab and peanut OIT or peanut OIT alone and was therefore counted twice  ^†^ - One study randomised children to receive either peanut OIT at dose of 300 mg or 3000 mg peanut protein daily and was therefore counted twice | | |

| **S3 Table. Comparison of characteristics between controlled and non-controlled studies** | | | |
| --- | --- | --- | --- |
| **Characteristic** | **Controlled** | **Non-Controlled** | **P Value** |
| Number of Studies | 9 | 18 | - |
| Subjects, median (range) | 31 (19-372) | 24 (9-270) | 0.105 |
| Rush Phase, n (%) |  |  | 0.219 |
| Yes | 4 (44) | 13 (72) |  |
| No | 5 (56) | 5 (28) |  |
| Co-Treatment^¥^ |  |  | 1.000 |
| Yes | 2 (22) | 5 (29) |  |
| No | 7 (78) | 12 (71) |  |
| Asthma (%), median (range) | 43 (41-69) | 62 (27-82) | 0.423 |
| Eczema (%), median (range) | 64 (0-83) | 57 (11-70) | 0.374 |
| Allergic Rhinitis (%), median (range) | 57 (14-79) | 55 (11-95) | 0.696 |
| Other Food Allergies (%), median (range) | 55 (23-83) | 49 (36-91) | 0.665 |
| Age (years), median (range) | 8 (6-15) | 8 (2-13) | 0.657 |
| Maintenance Dose  (mg/day)^†^, median (range) | 800 (300-5000) | 2000 (125-4000) | 0.821 |
| Entry OFC, n (%) |  |  | 0.445 |
| DBPCFC | 6 (67) | 7 (39) |  |
| Open | 1 (11) | 6 (33) |  |
| None | 2 (22) | 5 (28) |  |
| Baseline psIgE (kU/L), median (range) | 74.6 (14.3-162) | 55.4 (9.6-229) | 0.421 |
| Baseline SPT (mm) , median (range) | 10 (7-17.6) | 12 (8-16.5) | 0.470 |
| *Abbreviations: OFC, oral food challenge; DBPCFC, double blind placebo controlled food challenge; psIgE, peanut specific IgE; SPT, skin prick test*  ^¥^ Excludes 1 study that randomised subjects to receive co-treatment or placebo  ^†^ Excludes 1 study that randomised subjects to one of two maintenance doses | | | |

| S4 Table. Additional characteristics of included peanut oral immunotherapy studies | | | | | | | | | | | | | | | |
| --- | --- | --- | --- | --- | --- | --- | --- | --- | --- | --- | --- | --- | --- | --- | --- |
| **Study** | **Median**  **Age**  **(years)** | **Median**  **psIgE**  **(median)** | **Median**  **SPT**  **(median)** | **Asthma**  **(%)** | **Eczema**  **(%)** | **Other**  **Food**  **Allergies**  **(%)** | **Allergic**  **Rhinitis**  **(%)** | **Subjects**  **Receiving**  **OIT, N** | **Discontinued**  **Due to AE, n** | **Reached**  **Target**  **Dose, n** | **Passed**  **Supervised**  **Exit OFC, n** | **Reported AE Data** | | | |
|  |  |  |  |  |  |  |  |  |  |  |  | **Frequency** | | **Rate** | |
|  |  |  |  |  |  |  |  |  |  |  |  | **Overall** | **Timing** | **Overall** | **Timing** |
| Anagnostou 2011 [UK] | 11 | 29.7 | 8.5 | 64 | 36 | - | 45 | 22 | 1 | 19 | 14 | Y | Y | Y | N |
| Anagnostou 2014 [UK] | 12.4 | 39.75 | 9 | 59 | 52 | 23 | 57 | 94 | 3 | 81 | 49 | Y | N | Y | N |
| Bird 2015 [USA] | 7 | 54.9 | - | 73 | 55 | - | 55 | 11 | 2 | 9 | 9 | Y | Y | Y | N |
| Bird 2018 [USA] | 7 | 64.3 | 14 | 41 | 66 | 83 | 62 | 29 | 4 | 23 | 18 | N | N | Y | N |
| Blumchen 2010 [Germany] | 5.6 | 95.6 | - | 65 | - | 74 | - | 23 | 4 | 14 | - | Y | Y | Y | N |
| Blumchen 2019 [Germany] | 6.6 | 89.5 | 8 | 42 | 61 | 29 | 45 | 31 | 2 | 15 | 13 | Y | N | Y | N |
| Fauquert 2018 [France] | 14.5 | 162 | 12.4 | 43 | 0 | 28 | 14 | 21 | 2 | 17 | 17 | N | N | Y | Y |
| Hofmann 2009 [USA] | 4.8 | - | - | 68 | 64 | - | 57 | 28 | 1 | 20 | - | Y | Y | Y | Y |
| Howe 2019 [USA] | 10.82 | 54 | - | - | - | 68 | - | 50 | 0 | 50 | - | Y | N | N | N |
| Jones 2009 [USA] | 4.8 | 85.4 | - | 62 | 69 | 55 | 62 | 39 | 4 | 29 | 27 | Y | Y | Y | Y |
| Kukkonen 2017 [Finland] | 8.3 | 74.6 | 10 | 69 | 67 | 62 | 67 | 39 | 4 | 33 | 26 | N | N | Y | Y |
| MacGinnitie 2017 [USA]; Oral OIT alone arm | 10 | 91 | 10 | - | - | - | - | 8 | 1 | 1 | 1 | Y | N | Y | N |
| MacGinnitie 2017 [USA]; Omalizumab arm | 10 | 88 | 11 | - | - | - | - | 27 | 2 | 23 | 22 | Y | N | Y | N |
| Nachshon 2018 [Israel] | 6 | - | 8 | 39 | - | 49 | - | 145 | 7 | 113 | 95 | N | N | Y | N |
| Nagakura 2018a [Japan] | 8.5 | 38.5 | 9 | 50 | 59 | 36 | 55 | 22 | 0 | 22 | - | Y | Y | Y | Y |
| Nagakura 2018b [Japan] | 9.6 | 55.4 | 12 | 33 | 46 | - | 67 | 24 | 0 | 24 | - | Y | Y | Y | Y |
| Narisety 2015 [USA] | 11.1 | 169 | 12 | 82 | 55 | 91 | 95 | 11 | 3 | 7 | - | Y | Y | Y | N |
| Nozawa 2014 [Japan] | 9 | 30.82 | - | 56 | 63 | 43.7 | 25 | 18 | 2 | 16 | - | N | Y | Y | Y |
| PALISADE 2018 [North America and Europe] | - | 69 | 11 | 53 | - | 66 | - | 372 | 43 | 294 | 250 | N | N | Y | Y |
| Reier-Nilsen 2019 [Norway] | 10.1 | 110.6 | 9.8 | 42 | 83 | 47 | 26 | 57 | 9 | 12 | - | Y | N | Y | N |
| Schneider 2013 [USA] | 10 | 229 | 16.5 | - | - | 46 | - | 13 | 1 | 12 | 12 | Y | N | Y | Y |
| Tang 2015 [Australia] | 6.1 | 14.3 | 17.6 | 42 | 58 | - | - | 31 | 0 | 29 | 26 | N | N | Y | Y |
| Tao 2017 [Australia] | 13 | 9.6 | 12 | - | - | - | - | 14 | 0 | 11 | 11 | N | N | Y | N |
| Varshney, 2011 [USA] | 7 | 106 | 7 | 68 | 74 | 74 | 79 | 19 | 3 | 16 | 15 | N | Y | Y | Y |
| Vickery 2017 [USA]; 300 mg dose arm | - | 22.4 | 10.5 | 20 | 60 | - | 20 | 20 | - | 19 | 17 | N | N | Y | Y |
| Vickery 2017 [USA]; 3000 mg dose arm | - | 12.3 | 12.5 | 35 | 82 | - | 24 | 17 | - | 13 | 13 | N | N | Y | Y |
| Wasserman 2019 [USA] | 7 | - | - | 64 | - | - | - | 270 | 31 | 211 | - | N | N | Y | Y |
| Yu 2012 [USA] | 7.5 | 66 | 12.5 |  |  |  |  | 24 | 1 |  |  | Y | Y | Y | Y |
| Zhong 2019 [Singapore] | 8 | 100 | 15 | 56 | 11 | 44 | 11 | 9 | 0 | 7 | 6 | N | N | N | N |

| S5 Table. Meta-regression of the risk of adverse event causing treatment discontinuation according to study level characteristics | | | | | |
| --- | --- | --- | --- | --- | --- |
|  | **Withdraw due to Adverse Event** | | | | |
| **Comparison** | **Studies** | **Coefficient^†^ (95% CI)** | **I^2^ (%)** | **Variance**  **explained**  **(R^2^, %)** | **P Value** |
| psIgE (median, per every 10 kU/L) | 24 | 0.014 (0.006 to 0.024) | 34.1 | 45.6 | **0.0059** |
| SPT (median, per every 1 mm) | 20 | -0.007 (-0.026 to 0.012) | 45.4 | 0.00 | 0.4557 |
| Target Maintenance Dose (per every 100 mg) | 23 | 0.002 (-0.001 to 0.005) | 52.6 | 0.00 | 0.2245 |
| Age (median, per every year) | 26 | -0.005 (-0.021 to 0.012) | 47.6 | 0.00 | 0.5544 |
| Asthma (%, per every 10%) | 22 | 0.032 (-0.001 to 0.063) | 34.1 | 28.9 | **0.0402** |
| Other Food Allergies (%, per every 10%) | 17 | 0.024 (-0.005 to 0.052) | 47.5 | 14.4 | 0.0989 |
| Allergic Rhinitis (%, per every 10%) | 17 | 0.004 (-0.023 to 0.030) | 29.9 | 0.00 | 0.7889 |
| Eczema (%, per every 10%) | 18 | 0.019 (-0.010 to 0.047) | 33.0 | 16.2 | 0.2044 |
| **^†^** *Univariable meta-regression performed using Freeman-Tukey double arcsine transformed proportions* | | | | | |

| S6 Table. Frequency of different types of adverse events according to study characteristics | | | | | | | | |
| --- | --- | --- | --- | --- | --- | --- | --- | --- |
|  | **Frequency of Adverse Events Requiring Treatment** | | | | **Frequency Requiring Epinephrine** | | | |
| **Comparison** | **Studies** | **n/1,000 doses**  **(95% CI)** | **I^2^** | **p value** | **Studies** | **n/1,000 doses**  **(95% CI)** | **I^2^** | **p value** |
| Overall | 8 | 11.3 (5.4-19.5) | 99.0 | NA | 15 | 0.20 (0.08-0.37) | 64.4 | NA |
| Rush Phase |  |  |  |  |  |  |  |  |
| Yes | 5 | 12.6 (7.3-19.3) | 97.2 | 0.723 | 8 | 0.34 (0.14-0.63) | 60.9 | **0.022** |
| No | 3 | 9.4 (0.3-31.1) | NA |  | 7 | 0.06 (0.00-0.21) | 49.9 |  |
| Co-Treatment |  |  |  |  |  |  |  |  |
| Yes | 2 | 19.4 (17.3-21.6) | NA | **0.027** | 5 | 0.28 (0.04-0.67) | 63.0 | 0.583 |
| No | 6 | 9.2 (3.3-17.9) | 99.0 |  | 11 | 0.14 (0.02-0.35) | 67.4 |  |
| Intervention Blinding |  |  |  |  |  |  |  |  |
| Yes | 1 | 1.9 (1.2-2.8) | NA | NA | 1 | 0.00 (0.00-0.31) | NA | NA |
| No | 7 | 13.4 (6.7-22.3) | 98.9 |  | 14 | 0.23 (0.10-0.40) | 60.6 |  |
| Maintenance Dose  (mg/day) |  |  |  |  |  |  |  |  |
| < 1000 | 8 | 11.3 (5.4-19.5) | 99.0 | NA | 9 | 0.08 (0.01-0.19) | 40.7 | **0.001** |
| ≥ 1000 | - | - | - |  | 6 | 0.52 (0.28-0.82) | 21.3 |  |
| Entry OFC |  |  |  |  |  |  |  |  |
| DBPCFC | 4 | 14.6 (4.0-31.6) | 99.2 | 0.361 | 8 | 0.24 (0.06-0.51) | 64.7 | 0.667 |
| Open | 2 | 7.9 (6.8-9.2) | NA |  | 3 | 0.22 (0.00-1.05) | NA |  |
| None | 2 | 7.2 (6.2-8.3) | NA |  | 4 | 0.14 (0.02-0.35) | 36.4 |  |
| Baseline psIgE (kU/L) |  |  |  |  |  |  |  |  |
| <60 | 4 | 17.2 (5.0-36.6) | 99.4 | 0.144 | 6 | 0.11 (0.00-0.31) | 82.3 | 0.260 |
| ≥60 | 4 | 6.7 (2.9-11.9) | 97 |  | 9 | 0.27 (0.08-0.55) | 69.9 |  |
| Baseline SPT (mm) |  |  |  |  |  |  |  |  |
| <12 | 4 | 10.6 (1.7-26.9) | 99.4 | NA | 6 | 0.21 (0.03-0.52) | 77.5 | 0.224 |
| ≥12 | 1 | 24.6 (21.5-28.2) | NA |  | 4 | 0.47 (0.11-1.03) | 53.3 |  |
| *Abbreviations: OFC, oral food challenge; DBPCFC, double blind placebo controlled food challenge; psIgE, peanut specific IgE; SPT, skin prick test* | | | | | | | | |

| S7 Table. Meta-regression of the risk of different types of adverse events according to study level characteristics | | | | | | | | | | |
| --- | --- | --- | --- | --- | --- | --- | --- | --- | --- | --- |
|  | **Adverse Event Requiring Treatment** | | | | | **Adverse Event Requiring Epinephrine** | | | | |
| **Comparison** | **Studies** | **Coefficient^†^**  **(95% CI)** | **I^2^**  **(%)** | **Variance**  **explained**  **(R^2^, %)** | **p value** | **Studies** | **Coefficient^†^**  **(95% CI)** | **I^2^**  **(%)** | **Variance**  **explained**  **(R^2^, %)** | **p value** |
| psIgE  (median, per every 10 kU/L) | 7 | 0.002  (-0.002 to 0.006) | 79.1 | 2.4 | 0.3172 | 23 | 0.013  (-0.001 to 0.025) | 59.2 | 20.6 | **0.0247** |
| SPT  (median, per every 1 mm) | 6 | -0.014  (-0.103 to 0.076) | 80.9 | 0 | 0.765 | 19 | 0.009  (-0.014 to 0.033) | 67.3 | 0.00 | 0.4334 |
| Target Maintenance Dose  (per every 100 mg) | 7 | -0.001  (-0.012 to 0.011) | 82.8 | 0 | 0.9172 | 24 | 0.004  (-0.001 to 0.008) | 70.5 | 10.3 | 0.0595 |
| Age  (median, per every year) | 7 | -0.042  (-0.058 to -0.026) | 0 | 100 | **<0.001** | 25 | -0.007  (-0.029 to 0.015) | 73.2 | 12.0 | 0.5483 |
| Asthma  (%, per every 10%) | 6 | -0.018  (-0.121 to 0.084) | 83.6 | 0 | 0.7275 | 21 | 0.003  (-0.002 to 0.007) | 75.1 | 0.0 | 0.2439 |
| Other Food Allergies  (%, per every 10%) | 5 | 0.055  (-0.001 to 0.112) | 57.1 | 65.3 | 0.0562 | 16 | 0.002  (-0.002 to 0.005) | 73.6 | 0.0 | 0.4427 |
| Allergic Rhinitis  (%, per every 10%) | 6 | -0.011  (-0.099 to 0.077) | 84.2 | 0 | 0.8085 | 16 | 0.002  (-0.002 to 0.005) | 53.2 | 0.00 | 0.3208 |
| Eczema  (%, per every 10%) | 6 | 0.179  (0.108 to 0.251) | 0 | 100 | **<0.001** | 17 | 0.001  (-0.003 to 0.004) | 49.7 | 0.00 | 0.6371 |
| *Abbreviations: psIgE, peanut specific IgE; SPT, skin prick test*  **^†^** *Univariable meta-regression performed using Freeman-Tukey double arcsine transformed proportions* | | | | | | | | | | |

| S8 Table. Meta-regression of the frequency of different types of adverse events according to study level characteristics | | | | | | | | | | |
| --- | --- | --- | --- | --- | --- | --- | --- | --- | --- | --- |
|  | **Frequency of Adverse Events Requiring Treatment** | | | | | **Frequency Requiring Epinephrine** | | | | |
| **Comparison** | **Studies** | **Coefficient^†^**  **(95% CI)** | **I^2^**  **(%)** | **Variance**  **explained**  **(R^2^, %)** | **p value** | **Studies** | **Coefficient^†^**  **(95% CI)** | **I^2^**  **(%)** | **Variance**  **explained**  **(R^2^, %)** | **p value** |
| psIgE  (median, per every 10 kU/L) | 7 | -0.009  (-0.024 to 0.006) | 99.1 | 0 | 0.2366 | 14 | 0.001  (0.000 to 0.002) | 63.0 | 16.8 | 0.0636 |
| SPT  (median, per every 1 mm) | 5 | 0.017  (-0.022 to 0.056) | 99.4 | 0.06 | 0.4021 | 10 | 0.003  (0.000 to 0.005) | 59.5 | 44.5 | **0.0243** |
| Target Maintenance Dose  (per every 100 mg) | 7 | 0.001  (-0.012 to 0.013) | 99 | 0 | 0.9250 | 14 | 0  (0.000 to 0.001) | 53.4 | 21.6 | **0.0430** |
| Age  (median, per every year) | 8 | 0.006  (-0.008 to 0.019) | 99.1 | 0 | 0.4096 | 15 | 0.001  (-0.001 to 0.003) | 66.5 | 0.00 | 0.5093 |
| Asthma  (%, per every 10%) | 8 | -0.001  (-0.032 to 0.03) | 99.1 | 0 | 0.9343 | 11 | 0.002  (-0.002 to 0.006) | 62.4 | 0.00 | 0.3391 |
| Other Food Allergies  (%, per every 10%) | 5 | 0.01  (-0.002 to 0.023) | 96.9 | 28.6 | 0.1042 | 9 | 0.001  (-0.002 to 0.004) | 71.95 | 0.00 | 0.4524 |
| Allergic Rhinitis  (%, per every 10%) | 7 | 0.004  (-0.05 to 0.057) | 99.3 | 0 | 0.896 | 10 | 0.002  (-0.001 to 0.006) | 62.71 | 0.00 | 0.1413 |
| Eczema  (%, per every 10%) | 7 | -0.034  (-0.064 to -0.004) | 98.8 | 28.2 | **0.0248** | 10 | 0.002  (-0.002 to 0.006) | 61.58 | 0.00 | 0.4550 |
| *Abbreviations: psIgE, peanut specific IgE; SPT, skin prick test*  **^†^** *Univariable meta-regression performed using Freeman-Tukey double arcsine transformed proportions* | | | | | | | | | | |

| S9 Table. Pooled proportion of participants able to reach the target maintenance dose or pass supervised exit oral food challenge according to study characteristics | | | | | | | | | | |
| --- | --- | --- | --- | --- | --- | --- | --- | --- | --- | --- |
|  | **Reach Target Maintenance Dose** | | | | **Pass Supervised Exit OFC** | | | | | |
| **Comparison** | **Studies** | **% (95% CI)** | **I^2^** | **p value** | **Studies** | **% (95% CI)** | | | **I^2^** | **p value** |
| Overall | 26 | 80.9 (74.2-86.8) | 86.2 | NA | 17 | 68.9 (63.5-74.1) | | | 53.0 | NA |
| Rush Phase |  |  |  |  |  |  | | |  |  |
| Yes | 16 | 82.6 (77.5-87.2) | 65.5 | 0.545 | 9 | 71.3 (65.9-76.4) | | | 36.9 | 0.208 |
| No | 10 | 77.1 (56.4-93) | 93.3 |  | 8 | 64.3 (53.8-74.3) | | | 54.3 |  |
| Co-Treatment |  |  |  |  |  |  | | |  |  |
| No | 19 | 72.7 (64.7-80) | 84.9 | **<0.001** | 13 | 65.6 (58.5-72.3) | | | 64.9 | **0.035** |
| Yes | 8 | 95.0 (87.6-99.6) | 66.7 |  | 5 | 78.7 (68.8-87.3) | | | 25.4 |  |
| *Antihistamine Alone* | *3* | *97.4 (84.1-100)* | *NA* |  | *1* | *66.7 (51.0-79.4)* | | | *NA* |  |
| *Antihistamine + Montelukast* | *1* | *100 (85.1-100)* | *NA* |  | *-* | *-* | | | *-* |  |
| *Omalizumab* | *2* | *87.9 (75.1-96.9)* | *NA* |  | *2* | *85.5 (72.1-95.4)* | | | *NA* |  |
| *Probiotic* | *2* | *91.6 (79.7-99.1)* | *NA* |  | *2* | *81.1 (66.6-92.6)* | | | *NA* |  |
| Intervention Blinding |  |  |  |  |  |  | | |  |  |
| Yes | 6 | 78.6 (67.7-87.9) | 72.4 | 0.687 | 6 | 69.1 (58.1-79.1) | | | 67.7 | 0.952 |
| No | 20 | 81.6 (72.7-89.2) | 88.3 |  | 11 | 68.9 (62.1-75.4) | | | 45.4 |  |
| Maintenance Dose  (mg/day) |  |  |  |  |  |  | | |  |  |
| < 1000 | 15 | 84.8 (77.0-91.3) | 82.6 | 0.178 | 10 | 64.7 (57.5-71.7) | | | 56.0 | 0.054 |
| ≥ 1000 | 12 | 75.9 (63.6-86.5) | 87.6 |  | 8 | 74.9 (67.6-81.7) | | | 26.4 |  |
| Entry OFC |  |  |  |  |  |  | | |  |  |
| DBPCFC | 13 | 79 (67.4-88.7) | 89.0 | 0.597 | 9 | 67.3 (60.2-74.1) | | | 49.6 | 0.245 |
| Open | 7 | 79.3 (64.6-91.2) | 79.9 |  | 5 | 66.8 (52.8-79.5) | | | 67 |  |
| None | 6 | 86.1 (73.4-95.5) | 86.35 |  | 3 | 76.8 (67.1-85.3) | | | NA |  |
| Baseline psIgE (kU/L) |  |  |  |  |  |  | | |  |  |
| <60 | 9 | 93.1 (86.6-97.9) | 66.7 | **<0.001** | 6 | 72.9 (58.8-85.1) | | | 72.6 | 0.533 |
| ≥60 | 14 | 70.7 (59.2-81) | 86.9 |  | 10 | 68 (61.2-74.5) | | | 42.6 |  |
| Baseline SPT (mm) |  |  |  |  |  |  |  |  |  |  |
| <12 | 11 | 76.6 (64.8-86.7) | 91.4 | 0.216 | 9 | 64.6 (58.0-70.9) | | | 60.1 | **0.030** |
| ≥12 | 8 | 86.8 (76.5-94.8) | 53.9 |  | 6 | 78.0 (68.4-86.5) | | | 20.4 |  |
| *Abbreviations: OFC, oral food challenge; DBPCFC, double blind placebo controlled food challenge; psIgE, peanut specific IgE; SPT, skin prick test* | | | | | | | | | | |

| S10 Table. Meta-regression of the pooled proportion of participants able to reach the target maintenance dose or pass supervised exit oral food challenge according to study level characteristics | | | | | | | | | | |
| --- | --- | --- | --- | --- | --- | --- | --- | --- | --- | --- |
|  | **Reach Maintenance Dose** | | | | | **Pass Supervised OFC** | | | | |
| **Comparison** | **Studies** | **Coefficient^†^**  **(95% CI)** | **I^2^**  **(%)** | **Variance**  **explained**  **(R^2^, %)** | **p value** | **Studies** | **Coefficient^†^**  **(95% CI)** | **I^2^**  **(%)** | **Variance**  **explained**  **(R^2^, %)** | **p value** |
| psIgE  (median, per every 10 kU/L) | 23 | -0.015  (-0.033 to 0.002) | 85.5 | 19.0 | 0.0881 | 16 | 0.0052  (-0.008 to 0.018) | 57.3 | 0 | 0.4273 |
| SPT  (median, per every 1 mm) | 19 | 0.017  (-0.017 to 0.052) | 87.3 | 0.00 | 0.3255 | 15 | 0.0257  (0.007 to 0.044) | 40.4 | 49.5 | **0.0058** |
| Target Maintenance Dose  (per every 100 mg) | 24 | -0.007  (-0.013 to -0.002) | 82.5 | 16.6 | **0.0088** | 16 | 0.0043  (0.000 to 0.009) | 33.3 | 26.4 | 0.0618 |
| Age  (median, per every year) | 25 | 0.007  (-0.025 to 0.039) | 87.1 | 0.00 | 0.6693 | 16 | -0.005  (-0.026 to 0.016) | 55.2 | 0 | 0.6453 |
| Asthma  (%, per every 10%) | 22 | -0.015  (-0.074 to 0.044) | 86.0 | 0.00 | 0.6177 | 14 | -0.011  (-0.059 to 0.037) | 57 | 0 | 0.6500 |
| Other Food Allergies  (%, per every 10%) | 17 | -0.001  (-0.062 to 0.059) | 90.4 | 0.00 | 0.9616 | 11 | 0.023  (-0.01 to 0.056) | 48 | 19.9 | 0.1657 |
| Allergic Rhinitis  (%, per every 10%) | 17 | 0.019  (-0.037 to 0.075) | 87.1 | 7.8 | 0.5028 | 11 | -0.015  (-0.057 to 0.027) | 56.9 | 1.4 | 0.4893 |
| Eczema  (%, per every 10%) | 18 | -0.029  (-0.087 to 0.029) | 86.0 | 14.5 | 0.3196 | 12 | -0.004  (-0.045 to 0.038) | 64.9 | 0 | 0.8581 |
| *Abbreviations: psIgE, peanut specific IgE; SPT, skin prick test*  **^†^** *Univariable meta-regression performed using Freeman-Tukey double arcsine transformed proportions* | | | | | | | | | | |
